# Ammonothermal Crystal Growth of Functional Nitrides for Semiconductor Devices: Status and Potential

**DOI:** 10.3390/ma17133104

**Published:** 2024-06-25

**Authors:** Thomas Wostatek, V. Y. M. Rajesh Chirala, Nathan Stoddard, Ege N. Civas, Siddha Pimputkar, Saskia Schimmel

**Affiliations:** 1 Friedrich-Alexander-Universität Erlangen-Nürnberg, Chair of Electron Devices (LEB), Cauerstraße 6, 91058 Erlangen, Germany; 2 Department of Materials Science and Engineering, Lehigh University, 5 E Packer Avenue, Bethlehem, PA 18015, USA

**Keywords:** ammonothermal, synthesis, nitrides, semiconductors, solubility, in situ monitoring, crystal, high-pressure technology, supercritical fluid, GaN

## Abstract

The state-of-the-art ammonothermal method for the growth of nitrides is reviewed here, with an emphasis on binary and ternary nitrides beyond GaN. A wide range of relevant aspects are covered, from fundamental autoclave technology, to reactivity and solubility of elements, to synthesized crystalline nitride materials and their properties. Initially, the potential of emerging and novel nitrides is discussed, motivating their synthesis in single crystal form. This is followed by a summary of our current understanding of the reactivity/solubility of species and the state-of-the-art single crystal synthesis for GaN, AlN, AlGaN, BN, InN, and, more generally, ternary and higher order nitrides. Investigation of the synthesized materials is presented, with a focus on point defects (impurities, native defects including hydrogenated vacancies) based on GaN and potential pathways for their mitigation or circumvention for achieving a wide range of controllable functional and structural material properties. Lastly, recent developments in autoclave technology are reviewed, based on GaN, with a focus on advances in development of in situ technologies, including in situ temperature measurements, optical absorption via UV/Vis spectroscopy, imaging of the solution and crystals via optical (visible, X-ray), along with use of X-ray computed tomography and diffraction. While time intensive to develop, these technologies are now capable of offering unprecedented insight into the autoclave and, hence, facilitating the rapid exploration of novel nitride synthesis using the ammonothermal method.

## 1. Introduction

Electronic devices play an important role in modern society, and so do processes for the synthesis of semiconductor materials needed for making electronic devices. Several trends further increase the demand for energy- and resource-efficient solutions for harvesting, converting, distributing, and utilizing electric energy. These trends include electrification, digitalization, and the increasing demand on computing power for applications of artificial intelligence, to name just a few. While silicon has been the backbone of electronics and will remain important in cost-driven applications, silicon-based devices are approaching their physical limits.

The global need to reduce CO_2_ emissions creates an increasing momentum to focus on the energy-efficiency of devices and systems. Besides environmental aspects, efforts to strengthen energy sovereignty are an additional driving force to aim for an efficient use of electrical energy, as well as to favor the use of earth-abundant elements, preferably with diverse options for supply chains.

To improve energy-efficiency, to widen the device design space by novel physical properties, and to meet the above-outlined additional criteria, new materials are increasingly being researched.

This review delivers an overview of the synthesis and solubility of various nitrides that are accessible via the ammonothermal method (Section 2, Section 3 and Section 4). In the case of GaN, as the most studied and most reviewed ammonothermally grown material, we give only a compact overview of the state-of-the-art uses and the available literature, except for aspects that have never, or not recently, been reviewed in detail. Accordingly, the sections on point defects (Section 5), reactor technology (Section 6), and in situ monitoring (Section 7) are based on GaN and may contain generalizable information for other nitrides. These sections are almost exclusively based on GaN-specific literature, due to the lack of literature on these aspects for most other ammonothermally synthesized materials.

### 1.1. Potential of Functional Nitrides for Semiconductor Devices

The III-nitrides have become one of the main and most developed material systems for power electronics based on being (ultra-)wide bandgap semiconductors and their subsequent potential to improve energy-efficiency beyond the capabilities of silicon-based devices [1]. To further widen the material parameter and design space, novel nitrides that are lattice matched to GaN or AlN represent a promising class of materials, as they could be integrated with the maturing material platforms of GaN and, prospectively, AlN. In some cases, their ternary and multinary alloys hold promise to surpass not only the functional properties of competing classes of materials, but also the binary III-nitrides. Examples of areas in which ternary and multinary nitrides could overcome existing limitations include insufficient dielectric strength and electrical conductivity (that is, p- and n-type dopability with sufficient charge carrier mobilities) for power electronic devices, preventing devices with higher energy-efficiency [2,3], insufficient piezoelectric coefficients for piezoacoustic devices [4]; lack of ferroelectrics with high performance and good technological compatibility with a reasonably established semiconductor technology for, e.g., memory and micro/nano-actuator applications [5], too low thermal conductivities and electro-optical coefficients for nonlinear optical materials [6], and too high coercive field strength for energy-efficient data storage and neuromorphic computing devices [7].

**Figure 1:**
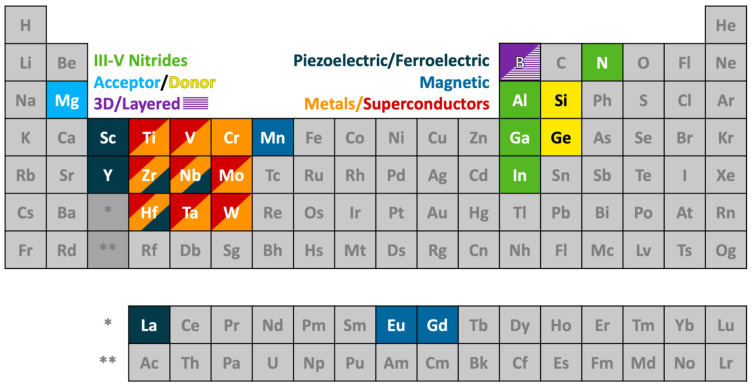
Property design space of “new” nitrides as of 2019 by D. Jena et al. [8]. Periodic table with constituents of “traditional” III-nitride semiconductors and associated dopants highlighted in green and bright blue/yellow, respectively. The other highlighting colors refer to physical properties and the usage of the elements in functional nitride materials. Reprinted from [8] under Creative Commons Attribution 4.0 license.

Multiple binary and ternary nitride materials that exhibit material properties of interest have emerged, which could be integrated into the GaN or AlN semiconductor platform. These are summarized in Figure 1. The wide material parameter design space facilitates the creation of various heterostructures of semiconducting (e.g., III-N [8], II-IV-N_2_ [9,10]), piezo- and ferroelectric (e.g., AlScN [4,5,8]), magnetic (e.g., Mn-IV-N_2_ [9]), and superconducting (e.g., NbN [8]) materials.

**Figure 2:**
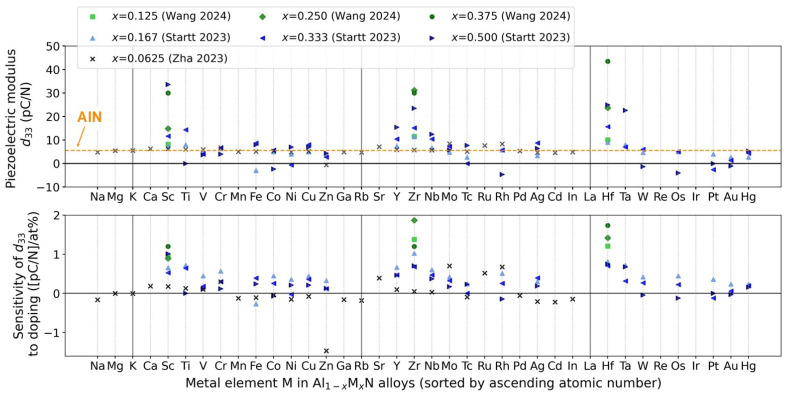
Sensitivity of the piezoelectric modulus *d_33_* to doping with transition metal elements according to theoretical studies by X.-H. Zha et al. [14], J. Startt et al. [13], and F. Wang et al. [15]. Note that the original data by X.-H. Zha et al. represent absolute values, whereas those by J. Startt et al. represent changes of *d_33_* with respect to pure AlN. This was considered when creating the graphic. The dashed orange line in the upper subplot indicates the piezoelectric modulus of pure wurtzite AlN.

Nitride semiconductors with enhanced piezoelectric and ferroelectric properties are a particularly recent and exciting emerging topic [11,12]. Important catalysts for this development were the discovery of enhanced piezoelectric properties of AlScN by M. Akiyama in 2009 [4] and S. Fichtner’s first report of AlScN being not only piezo- but also ferroelectric in 2019 [5]. Meanwhile, additional AlN-based alloys are being explored, with regard to enhanced piezoelectric properties. Examples are alloys (solid solutions) containing B [11] or certain transition metals [13,14]. Specifically, X.-H. Zha et al. have investigated alloys of the composition Al_0.9375_TM_0.0625_N via density functional theory (DFT) calculations (using a 2 × 2 × 2 supercell of 32 atoms) and identified Ca, Cr, Sr, Mo, Ru, and Rh as promising transition metals (TM) for enhancing the piezoelectric modulus *d_33_*, in relation to pure AlN, indicating a significant enhancement beyond that achievable by the same concentration of Sc in the cases of Mo, Rh, Ru, Sr, and Cr (with Mo causing the most pronounced effect) [14]. Similarly, J. Startt et al. studied Al_1−x_TM_x_N alloys, likewise by DFT calculations (using a 3 × 3 × 2 supercell of 72 atoms), with the transition metal content *x* being 0.166, 0.333, and 0.5, respectively, and TM representing different earth-abundant d-block elements [13]. J. Startt et al. concluded that the group 4 metals Ti, Zr, and Hf induce large piezoelectric enhancements comparable to those caused by Sc, while the group 5 elements Nb and Ta also provide enhancements, but are less effective [13]. Very recently, F. Wang et al. also published a related DFT study (using a 2 × 2 × 3 supercells with 48 atoms), investigating the three ternary materials, AlScN, AlHfN, and AlZrN, as well as co-alloying AlN with Sc and either Hf or Zr [15]. Wang et al. reported a piezoelectric coefficient *d_33_* as large as 49.18 pC/N and 47.00 pC/N for (HfSc)_0.375_Al_0.625_N and (ZrSc)_0.375_Al_0.625_N, respectively, which is 164% and 156% larger than that of Sc_0.375_Al_0.625_N, and 926% and 885% larger than that of pure wurtzite AlN [15]. The piezoelectric moduli from X.-H. Zha et al., J. Startt et al., and F. Wang et al. [15] are jointly presented in Figure 2.

Together, the aforementioned studies substantiate that alloying of III-nitrides with transition metals other than Sc is promising for enhancing *d_33_* further, or for using more accessible elements. However, the theoretical predictions show significant discrepancies and further studies including experimental results and more reliable calculations (larger supercells or more representative selections of inequivalent supercells, see e.g., D. F. Urban et al. [16] for a discussion of this aspect, with the example of AlScN) are necessary to establish confidence regarding the effective choice of alloying elements.

**Figure 3:**
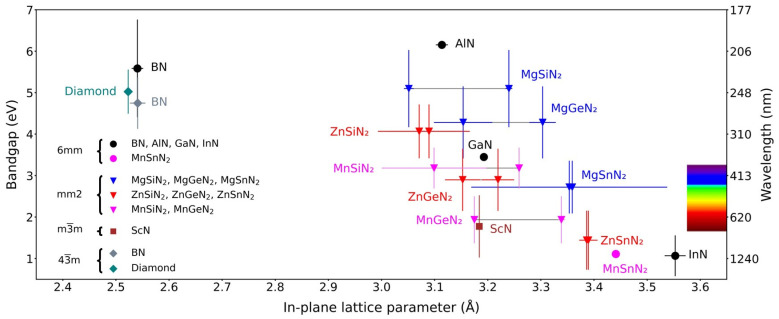
Bandgap and in-plane lattice constants for selected nitrides and lattice-matched materials (labeled with point groups (it is common in the literature to distinguish different polymorphs by an abbreviation of either the crystal system (such as h for hexagonal), or the crystal structure (such as w for wurtzite). We avoid distinguishing the different polymorphs by crystal system in order to avoid confusion, as both wurtzite and graphitic boron nitride would be referred to as h-BN in a consistent labeling by crystal system, while most readers would expect w-BN for BN in the wurtzite structure. The use of point groups allows consistent labeling of all discussed materials with a sufficient level of detail for our purposes.)). The two data points shown for the orthorhombic materials are the wurtzite-equivalent in-plane lattice parameters $a\sqrt{3}$ and *b*/2. Data were obtained from the following references: 6mm-BN [24,25,26], 6mm-AlN [18,27,28,29], 6mm-GaN [26,27,28,29], 6mm-InN [26,27,28,29], 6mm-MnSnN_2_ [30], mm2-MgSiN_2_ [31,32,33,34,35,36], mm2-MgGeN_2_ [31,33,34,35,36,37], mm2-MgSnN_2_ [17,33,34,38,39,40], mm2-ZnSiN_2_ [21,23,41,42,43,44], mm2-ZnGeN_2_ [21,23,41,42,44], mm2-ZnSnN_2_ [21,23,40,44,45,46,47], mm2-MnSiN_2_ [36,43,48,49,50], mm2-MnGeN_2_ [36,50,51], $\bar{4}3m$-BN [25,52,53], $m\bar{3}m$-ScN [8,18,54], and $\bar{4}3m$-diamond [3,55,56,57]. The error bars represent the standard deviation of values collected from the different references. Note that the lack of an error bar in the case of 6mm-MnSnN_2_ does not imply low uncertainty, but an insufficient amount of data for quantifying uncertainty in this way. Note as well that only the experimental bandgap data on MnSiN_2_ and MnGeN_2_ from [36] were used (as the spin-specific two bandgaps per material would have made the figure less readable), and that the calculated values for the bandgaps are considerably lower.

Motivated by the search for enhanced functional properties, novel heterostructures with III-nitrides, and earth-abundant semiconductors, heterovalent ternary nitrides of the stoichiometry II-IV-N_2_ [9,10] are increasingly being studied. They typically crystallize in orthorhombic, wurtzite-derived superstructures that feature in-plane lattice constants similar to those of GaN and/or AlN (Figure 3). More recently, additional polymorphs have been discovered for some of these materials (such as wurtzite and rocksalt MnSnN_2_ [17]), further widening the design space. In addition, this also opens up the possibility of heteroepitaxial integration of different phases of the same material with distinctly different properties, as O. Ambacher recently contemplated for AlScN [18]. Both the properties of ternary nitrides and their epitaxial relationships to different materials are of interest, as will be elaborated in the following paragraphs.

An important obstacle for realizing complementary field-effect devices in nitrides (implementation of CMOS logic directly in the wide bandgap nitride platform) is the low hole mobility in GaN [19]. In this context, II-IV-N_2_ compounds provide novel opportunities for strain engineering. For example, epitaxial growth of GaN on ZnGeN_2_ or MgSiN_2_ results in an inversion of the heavy hole band and split-off hole band, thereby lowering the effective hole mass in the compression direction and increasing the hole mobility in GaN by 50% and 260%, respectively [20]. In addition, electron and hole effective masses have been calculated for Zn-II-N_2_ (II = Si, Ge, Sn), indicating relatively low hole effective masses in the c-direction (Γ-Z, below 0.3 for ZnGeN_2_ [21], compared to 0.87 for GaN [22], both for the highest sub-band), but also a significant anisotropy with relatively high hole effective masses in the range of 2.5 to 6 in the Γ-X and Γ-Y directions. As an example of a heterostructure with a binary nitride in the wurtzite structure, ZnSiN_2_/AlN interfaces exhibit a larger conduction-band offset than AlGaN/GaN, in combination with a large polarization charge, and therefore hold promise to yield higher electron sheet densities [23].

According to calculations of elastic constants, both MgSiN_2_ and MgGeN_2_ are softer than their III-nitride counterparts AlN and GaN, which eases their heteroepitaxial growth on these binary nitrides [33]. In addition, MgSiN_2_ and Mg_2_PN_3_ (which likewise has already been synthesized via the ammonothermal method [58]) have recently been identified as promising multinary wurtzite-type ferroelectrics with comparatively low switching barriers, as needed for next-generation low-power computing [7].

The Mn-II-N_2_ alloy system is least explored, however it is known to exhibit spin polarization (resulting in spin-specific bandgaps according to DFT calculations), as Mn introduces a magnetic moment [36]. Both cation-ordered MnSiN_2_ [49] and MnGeN_2_ [30,59] maintain anti-ferromagnetic ordering above room temperature, whereas the cation-ordered MnSnN_2_ shows a magnetic transition at around 10 K [30]. The order or disorder in the cation sublattice appears to play an important role in the formation of polymorphs [30]. The ordering of the heterovalent cations has also been identified as a design parameter for electronic bandgaps, which was derived via a combined experimental and theoretical study on ZnSnN_2_ and MgSnN_2_ and should be applicable to a broad group of ternary heterovalent compounds [40].

Investigations on doping of the II-IV-N_2_ materials remain limited. Theoretical studies on ZnGeN_2_ suggest some similarities to the III-nitrides. Oxygen O_N_ is a shallow donor with a low formation energy and is thus likely to cause n-type conductivity in unintentionally doped (UID) samples, whereas the incorporation of Li on substitutional lattice sites is predicted to yield p-type doping [10].

**Figure 4:**
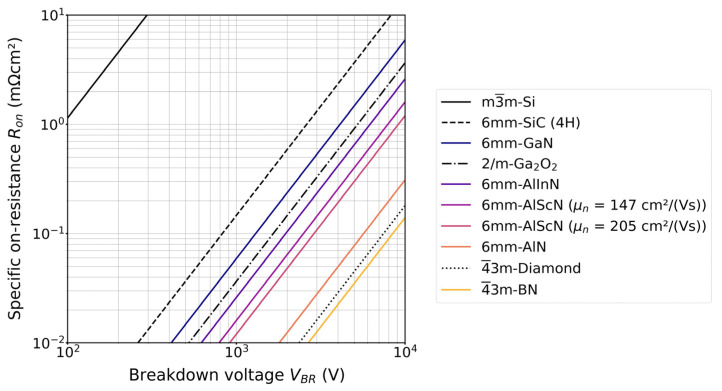
Contours of constant Baliga figure of merit (BFOM) for materials used or considered for power electronic applications. The lower right area represents higher BFOM (lower *R_on_* for a given *V_BR_*), hence better performance of low-frequency unipolar vertical power switches. Data by Tsao et al. ($\bar{4}3m$-diamond, $\bar{4}3m$-BN) [3] and Tansu et al. (6mm-Al_0.82_Sc_0.18_N, the composition lattice matched to 6mm-GaN, and Al_0.82_In_0.18_N) [60], respectively. For 6mm-SiC (specifically, 4H-SiC), 6mm-GaN, 2/m-Ga_2_O_3_ (β-Ga_2_O_3_), and 6mm-AlN, average values of both references are given.

A current frontier, specifically in energy-efficient power electronics, is ultra-wide bandgap (UWBG) semiconductors, adding to the interest in this subgroup of materials. The motivation for transitioning to materials with an even wider bandgap (*E_g_*) than GaN or SiC is that the critical electric field scales approximately as $E_{g}^{2}$ [3]. The Baliga figure of merit (BFOM) used to assess potential performance for low frequency, for example, unipolar power devices, therefore scales approximately as $E_{g}^{6}$ [3]. Materials for next generation power electronics are evaluated using the BFOM in Figure 4 by comparing the specific on-resistance, *R_on_*, with the breakdown voltage, *V_BR_*. The bottom right corner is most desired, i.e., lowest *R_on_* at the highest *V_BR_*. The two contours of constant BFOM shown for Al_0.82_Sc_0.18_N [60] differ by dislocation density (thus, different electron mobilities *μ_n_*). This highlights the importance of low dislocation densities for utilizing the full potential of a material. Currently, all UWBG materials are still under development and are actively being researched.

For UWBG Al*_x_*Ga_1−*x*_N alloys, the cohesive energy and subsequent bond strength increase rapidly with increasing Al-content. This complicates doping, due to the formation of deep levels and compensation. As a result, control of the electronic properties is more challenging, causing both n-type and p-type doping to remain active areas of research for Al-contents exceeding 80% [61]. Challenges for n-type doping appear to be related to the formation of DX centers (DX centers were initially thought of as a defect complex involving a substitutional donor and an anion vacancy [62], but are meanwhile thought to be due to a large lattice relaxation and the trapping of two electrons, causing the dopant to transition from a single donor to a single acceptor [63,64]) [65], whereas issues with p-type doping with Mg are caused by a significant deepening of the acceptor level, as well as the decreasing solubility of Mg with increasing Al-content [61]. Recently, p-type doping of AlN using Be at the Al-site has re-gained momentum due to experimental reports [66,67,68] of p-type conductivity. For both Si doping of Al-rich AlGaN in metal organic chemical vapor deposition (MOCVD) [69] and Be doping of AlN in metal-modulated epitaxy (MME) [66], improvements in doping efficiency appear to be closely linked to low growth temperatures (1050 to 1115 °C in MOCVD [69], 600 to 700 °C in MME [66]). The underlying mechanism is likely related to the dopant-induced strain and appears to favor substitutional incorporation on Al lattice sites (Be_Al_), as opposed to interstitial sites [66]. An analogous mechanism is likely responsible for the doping efficiency improvement of AlGaN:Si at low growth temperatures [69]. The effect of high temperatures driving light elements into interstitial sites is thought to be universal for light dopants in compound semiconductors [70].

### 1.2. Brief Overview of Methods for Nitride Synthesis and Crystal Growth

To realize the potential provided by the physical properties of nitride materials in practice, the choice and advancement of suitable methods for their synthesis and crystal growth plays a pivotal role. In particular, the availability of affordable large-area and high-quality native GaN substrates with controlled electrical properties remains a major roadblock [71].

For the synthesis of thin films, vapor-phase approaches, such as (plasma-assisted, ammonia-based, or metal-modulated) molecular beam epitaxy (MBE) [2,66,72] and metal organic vapor phase epitaxy (MOVPE) [69,70] are the primarily used techniques yielding high crystalline quality III-N with controllable layer thicknesses and dopant/alloying control, while reports on MBE [73,74] or MOVPE growth of other nitrides also exist [75,76]. Generally, these techniques are capable of yielding high quality material at temperatures below their decomposition temperature. This is particularly advantageous for nitrides that are prone to phase separation, such as III alloys with high indium content [77,78,79].

For the growth of thick films or relatively thin bulk crystals, sodium flux [80] and hydride vapor phase epitaxy (HVPE) are the most established techniques, which have been investigated with a strong focus on GaN thus far. Specifically for the bulk growth of GaN via HVPE, sodium flux, and ammonothermal methods, a review of the state-of-the-art aforementioned techniques was provided by R. Kucharski et al. in 2020 [81].

For the growth of thick bulk GaN crystals, the ammonothermal method is thus far the only technique having successfully demonstrated boules with diameters up to nearly 4 inches [82]. In the case of AlN, physical vapor transport (PVT) is, thus far, the main technique explored for bulk growth, that is, a technique based on the sublimation of the material. An important frontier in the PVT growth of AlN has been the expansion of crystal diameters, and remarkable progress has been made in this direction during the last few years [83].

Compared to PVT, the ammonothermal technique is performed at significantly lower temperatures (~600 °C vs. ~>2000 °C [84,85]) and in a different growth environment, offers opportunities to modify the incorporation of species into the growing boule (such as dopants), or leverage kinetic growth processes to modify native defect production or phase selection. One important drawback of the ammonothermal method is the observed lower growth rates (compared to PVT) [85,86]. However, this can be overcome by the simultaneous growth of a large number of crystals in one autoclave (more than 50 has been reported). A more detailed discussion of the ammonothermal method is provided in the following section.

### 1.3. Potential of the Ammonothermal Method for the Synthesis of Functional Nitrides Materials

**Figure 5:**
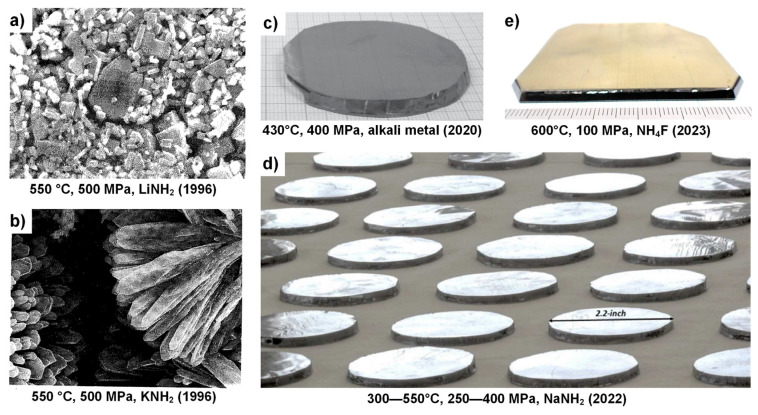
Development of ammonothermal crystal growth of GaN, starting from the initial synthesis experiments by Dwiliński et al. to recent achievements. (**a**) GaN crystallites a few micrometers in size, obtained with LiNH_2_ mineralizer (SEM image) [96]; (**b**) GaN crystallites of up to 25 µm length, obtained with KNH_2_ mineralizer (SEM image) [96]; (**c**) state-of-the-art GaN crystal grown using alkali metal mineralizers [97], (**d**) batch thereof to visualize the method’s capability for the simultaneous growth of many crystals [98], and (**e**) state-of-the-art GaN grown with NH_4_F mineralizer at comparatively low pressure [84]. All images reproduced from the respective references with permission; (**a**,**b**,**d**) under Creative Commons Attribution Non-Commercial 4.0 International License CC BY-NC 4.0, (**c**) © 2020 Elsevier B.V, (**e**) © 2022 The Japan Society of Applied Physics.

The ammonothermal method is a promising yet underexplored synthesis method for nitrides at low to moderate temperatures of about 400 to 900 °C. Its development has primarily been driven by the realization of GaN substrates with superior structural quality [81,87,88,89,90,91] and for exploratory nitride synthesis [92,93,94]. Despite the associated technical and scientific challenges, the method has seen a remarkable development since the initial reports on ammonothermal GaN growth by R. Dwiliński, H. Jacobs, and coworkers in 1995 [95], as illustrated in Figure 5.

From a crystal properties point of view, the key advantage of the ammonothermal crystal growth method is the exceptionally high structural quality that can be obtained (low amount of threading dislocations, large radius of curvature), whereas the main disadvantage is the difficulty of achieving high purity and low point defect concentrations [99,100,101]. Importantly, the ammonothermal method as an equilibrium process is suitable for obtaining seeds of excellent structural quality by self-nucleation, in both low-pressure [102,103] and high-pressure [104] variants of the method. Contrary to HVPE as a non-equilibrium process [71], the size of seeds can be increased by consecutive growth runs [102] by taking advantage of lateral growth in non-polar or semi-polar directions [105,106], which allows for simultaneous improvements in structural quality, particularly in the regions that extend laterally beyond the seed [81,86]. This can be exploited by starting from a slender seed obtained by a different growth technique (if available, such as HVPE in case of GaN), which can later be removed from the newly grown ammonothermal crystal, in order to use only the newly grown regions of improved structural quality in subsequent ammonothermal growth runs [81]. It is also possible to combine seeds via so-called tiling technology [107], developed earlier for HVPE [108]. Once a sufficient diameter with appropriate structural quality has been obtained, growth in a polar direction allows for the multiplication of seeds [105], which is important given the comparatively slow growth rates [86], motivating the simultaneous growth of many crystals to achieve cost-competitiveness [88]. The simultaneous growth of large numbers of crystals has proven effective for the well-established yet similar hydrothermal growth of quartz [109]. It is also feasible to use HVPE to speed up the multiplication process [110]. A relevant technological aspect is the importance of surface preparation for avoiding the formation of defect clusters, such as networks of high-density threading dislocations induced by subsurface damage in the seed crystal [107].

While initially developed for bulk GaN, the method is also increasingly being investigated for bulk growth of further binary nitrides with application potential as substrates for electronic devices (such as cubic BN [111]), as well as for the exploratory synthesis of ternary and multinary nitrides [92]. It is also worth noting that a deliberate incorporation of transition metals into nitride crystals during ammonothermal growth has already been achieved, which is of increasing interest given the recent developments in piezo- and ferroelectric nitrides, as described in Section 1.1. The first such report dates back to a study from 2001 which targeted GaN-based dilute magnetic semiconductors (DMS) such as Ga_1-*x*_Mn*_x_*N [112].

Besides the ability to use low growth temperatures, the synthesis conditions in the ammonothermal method (mineralizer, temperature, pressure) can be tailored to favor crystallization of the same material in a specific crystal structure, such as wurtzite or zincblende in the case of GaN [113]. While most ammonothermal research has so far focused on wurtzite GaN, both wurtzite and zincblende crystal structures emerge as interesting, from an application point of view, for some of the novel nitrides [18,114].

It should be noted that a synthesis method for self-nucleated bulk material with high structural quality, as demonstrated for GaN [102], is important even for materials that are more likely to be applied as thin films than as substrates. For many of the novel nitrides, small crystals of high quality with low defect concentrations and minimal internal stress are currently lacking, while relevant functional properties tend to be influenced by structural quality. The ferroelectric properties of AlScN are an example of a property of high current interest that was shown to be impacted by structural quality [115]. Even for InN, a bulk material of sufficient quality is not yet available [116]. High quality bulk samples are essential for an accurate experimental determination of fundamental properties, as needed for investigating prospective device applications.

In this review, we focus on the ammonothermal method as a technology for the synthesis and crystal growth of various nitrides with a variety of functional properties, while building on the much further developed example of GaN.

## 2. Ammonothermal Method

Having motivated the interest of the crystal growth community in the types and attributes of crystals that have been demonstrated using ammonothermal technology, let us take a step back and describe the operating principle and associated equipment of the technique. The tendency of nitrides to decompose into their base metals and triple-bonded nitrogen gas molecules limits the abilities of more traditional melt-growth techniques for bulk crystallization [28,117]. Only in extreme pressure, oxygen-free conditions can the formation of materials like GaN be achieved directly [118]. Therefore, a solution-based growth principle is much more appealing, and the ammonothermal method employs ammonia (NH_3_) as a nitrogen analog to water (H_2_O) [96,119], aimed at bypassing some of the difficulties of direct nitride formation.

In solution growth methods, the material constituents are typically dissolved using an appropriate solvent. The solution is then brought into a saturated or supersaturated condition by the manipulation of variables like temperature or pressure, or the selective removal of the pure solvent by methods such as evaporation or reverse osmosis [120]. Crystallization may take place spontaneously through nucleation, or may proceed on a seed crystal.

The hydrothermal method, used for making oxide crystals such as quartz, employs subcritical water as the solvent [121,122] and has long served as an enormously successful example of the synthesis of large numbers of bulk single crystals from a solvothermal solution, motivating the development of the ammonothermal method for nitride synthesis in an analogous way [123]. For nitrides and the ammonothermal method, ammonia is the equivalent option. In both hydrothermal and ammonothermal boule growth, temperature-dependent solubility is the key to success, and needs to enable the dissolution of suitable quantities of the material and the control of its transport via the thermal field in the reactor. In order to attain sufficient solubility of the desired solutes, the methods are operated in the sub- or supercritical fluid regime. Near their critical point, fluids generally have liquid-like dissolving power, in conjunction with transport properties that are in-between those of a gas and a liquid [124]. In supercritical fluids, the relatively high, liquid-like density enhances solubility [125], while temperature and pressure are sufficient to create an extremely mobile (i.e., diffusive [126] and convective [127]) and highly reactive fluid [126]. Most supercritical fluid applications function best in the region of high isothermal compressibility, not too far above the critical point [126]. The ammonothermal method, however, uses a parameter space far above the critical point [128]. As can be seen from the fluid properties and pressure/temperature ranges in Table 1, this differentiates the ammonothermal method from the hydrothermal method, which utilizes even slightly sub-critical conditions (*T/T_c_* < 1.0 and *p/p_c_* < 1.0). The reasons for this unusual process window have not yet been fully clarified. In this context, the recently increasing interest in the potential of supercritical geothermal resources [129] might lead to potentially transferable results. The conditions in supercritical geothermal reservoirs were estimated to be in the temperature range of 400 to 600 °C (*T/T_c_* = 1.07 to 1.60) and the pressure range of 20 to 30 MPa (*p/p_c_* = 0.91 to 1.36) [129,130], about the same absolute temperature but not pressure range. The interest in supercritical geothermal resources has already led to recent thorough investigations on solubilities of quartz under those conditions [129].

However, even the supercritical ammonia atmosphere is often not reactive enough to cause sufficient dissolution of the nitride source material for effective crystal growth. To further increase solubility, so-called “mineralizers” are added to the solution as a co-solvent to form soluble intermediates [133,134]. Mineralizers are termed acidic if they increase the concentration of [NH_4_]^+^ ions (this is the case for ammonium halides [133]). Accordingly, mineralizers that increase the concentration of [NH_2_]^-^ ions are called basic mineralizers (alkali and alkaline earth metals are typical basic mineralizers) [134]. The addition of mineralizers can dramatically increase the solubility through the formation of complex ions, due to the greater availability of [NH_2_]^−^ and [NH_4_]^+^ ions under ammonobasic and ammonoacidic conditions, respectively [119]. Regarding the second ion introduced with the mineralizer, the columns IA, IIA, or VIIA of the periodic table are convenient both for their reactivity with many elements, and for their lack of reactivity with nitrogen to form nitrides [135].

**Figure 6:**
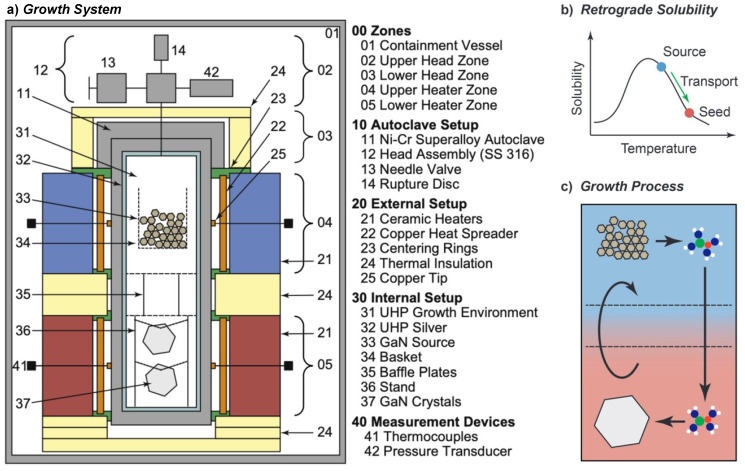
Representative diagram of an ammonothermal growth apparatus and mechanisms. (**a**) The growth system situated inside a containment vessel, wherein the high pressures of the process are monitored and managed safely. Two independent heat zones, 04 and 05, create the conditions necessary for dissolution, transport, and crystal growth. An ammonothermal system with retrograde solubility is depicted accordingly, with the seeds located in the hotter region. (**b**) A graph of solubility illustrating the availability and use of a retrograde temperature dependence, as is available with most basic mineralizers. (**c**) The cycle of the growth process in the supercritical fluid. Applied heat causes the hotter fluid (red) to rise on the perimeter. In the cooler upper zone, source material is dissolved at the higher solubility level. The cooler fluid (blue) then falls through the central region and delivers solute to the seed crystals, where the now supersaturated solution deposits new material. Note that, in the case of normal solubility, as is available with most acidic mineralizers at moderate temperatures, the hot zone remains at the bottom while the seeds are moved to the upper, cooler zone. Reproduced from [140], copyright (2014), with permission from Elsevier (left subfigure) and [87], © 2017 WILEY-VCH Verlag GmbH & Co. KGaA, Weinheim, Germany (right subfigure).

With enough solubility of the starting material achieved in the supercritical ammonia environment, and with mass transport facilitated by buoyancy, the last key element for successful ammonothermal growth is the availability of a solubility gradient as the driving force for crystallization. This solubility gradient, which needs to be established between the region containing the source material and the region containing the seed crystals, is usually created via a temperature gradient. A saturated solution is formed in the zone of the autoclave that contains the source material. When the convective flow transports the saturated fluid to another zone with lower solubility, typically provisioned with seed crystals, the solution becomes supersaturated. This supersaturation acts as the driving force for deposition on available surfaces, moving the solution back towards equilibrium. A schematic of the process and a representative experimental setup are shown in Figure 6, using the example of ammonothermal growth of bulk GaN, as this is the most developed ammonothermal bulk nitride growth process to date.

Since the ammonothermal method relies on the use of ammonia as the solvent, elevated pressures are required to prevent excessive decomposition of ammonia into hydrogen and nitrogen gases [136]. Higher pressures have generally yielded higher crystal growth rates for gallium nitride, at least with several acidic mineralizers [103,137]. Apart from preventing ammonia decomposition, the generally observed increase of solubilities in supercritical fluids with increasing solvent density or pressure [124,129] may also play a role.

A challenge of ammonothermal growth is the currently limited crystal growth rates. In the worst cases, some processes seem incapable of exceeding 30 µm/day on certain stable crystallographic facets. The fastest substantiated steady-state growth rates of GaN single crystal growth advertise 150–200 μm/day [84], although some higher claims exist. The aforementioned challenges can be compensated by growing many crystals in parallel, as demonstrated in industrial-scale hydrothermal processes, as well as ammonothermal processes approaching industrial scales [84,88,138,139].

Active observation of the ammonothermal process is also limited by the thick autoclave walls. In order to gain a more detailed understanding of the inner workings of the process, two approaches have been developed. The first is computational simulations, wherein the temperature distributions and convective flow patterns in the fluid can be predicted. For this approach, a review from 2021 is available [128]. The second approach is direct in situ observation of the hot, pressurized autoclave, which will be reviewed in Section 6.2.

As with any crystal growth process, limitations emerge for the design of systems and crystal grow processes based on the chemical reactivity of species to the nitriding environment. This is no different for the ammonothermal system, and knowledge of what materials can and will go into a solution offers opportunities for nitride crystal growth containing these elements, while limiting opportunities for use of these materials in their soluble forms (elemental form or as part of a specific compound) as functional elements in the system design. The next section discusses the current state of knowledge pertaining to the reactivity and solubility of elements under ammonothermal conditions.

## 3. Reactivity and Solubility of Elements under Ammonothermal Conditions

As already delineated in general, the solubility of substances is markedly enhanced under ammonothermal conditions, owing to the elevated pressure and temperature coupled with the unique solvent characteristics of supercritical ammonia and the use of mineralizers for solubility enhancement. This environment facilitates the dissolution of a wide range of materials, albeit with a broad variability across different elements and strongly depending on the mineralizer used. Ammonia and the mineralizer are a versatile solvent due to the formation of diverse complex species (so called intermediates) with dissolved elements, thereby modulating their solubility and chemical reactivity within the solution. Precise control of the element solubility is significant for controlling crystal growth rates, composition, and impurity incorporation, as well as for ensuring the longevity of the autoclaves.

Notably, solubility in ammonothermal solutions demonstrates a significant dependency on temperature and pressure, with alterations in these parameters exerting substantial effects on element solubility and chemical equilibria within the solution medium [132]. From the viewpoint of bulk crystal growth, the temperature dependency of solubility is particularly relevant, however, quantitative data are available only for GaN and even for GaN, those data are incomplete and partially contradictory [128]. Many studies from the past and recent times dealt with either crystal growth [88,102,105,140,141,142], synthesis of compounds [9,92,143] via ammonothermal method and their intermediates [119,133,144], dopant incorporation [87,145,146], corrosion resistance [147,148], or dissolution kinetics and solubility studies of GaN [149,150,151,152] or BN [111]. The references mentioned in this paragraph are not intended as a complete list, but rather as examples of the different types of information that are available in the literature, while a full list of references used in this section can be found in the Appendix A, Appendix B and Appendix C.

Before going further in this section, we describe our approach to data visualization using a periodic table, which will then be used to indicate whether a given element was used in any ammonothermal or closely related environment, and which qualitative information on the reactivity and solubility of the element can be derived from the available literature.

The available types of information were sorted into categories, with negative values representing the availability of data that discourage reactivity and solubility (high corrosion resistance), zero in case of no information being available, and positive values for literature indicating some level of reactivity or solubility. This numerical value is assigned for plotting purposes. It was defined so that it increases with increasing confidence in solubility and will be termed the reactivity and solubility value (R and S value).

Considering that the same element can have rather different reactivity (and, as a consequence, solubility) depending on its chemical environment in a solid, we distinguish between data obtained using elemental samples and those obtained using a compound containing the element. For this differentiation, we define subcategories for data on elemental and compound samples, respectively. The following five distinct categories will be used:

- Category C1 denotes elements or compounds exhibiting exceptional resistance to corrosion within the ammonothermal environment, either in their elemental or compound forms. Negative R and S values are assigned, as the available information suggests a lack of reactivity and solubility;
- Category C2 represents elements or compounds for which no data regarding their reactivity or solubility under ammonothermal conditions are available;
- Category C3 represents compounds or elementals that do undergo reactions under ammonothermal conditions, but no clear indication of significant transport was found. Typical examples are the corrosion of elements or compounds, or the formation of compounds without indications of transport, as is often the case for the synthesis of compounds in nano- or microcrystalline form;
- Category C4 indicates the formation of compounds that are soluble in liquid ammonia. Typical examples are the compounds that were synthesized under ammonothermal conditions and were reported to likely represent soluble intermediates during ammonothermal synthesis and crystal growth, but at times room temperature or even lower temperatures, but on the whole, ranging from 197–873 K [119];
- Category C5 represents elements for which a clear indication of transport under ammonothermal conditions was found. Examples are mineralizer cation in the case of ammonobasic growth, mineralizer anion in the case of ammonoacidic growth, impurities incorporated in grown crystals (mostly GaN, as there are hardly any investigations on impurities in other materials grown via solute transport), elements for which dedicated solubility studies are available, as well as nitrogen and hydrogen as ubiquitous elements in ammonothermal fluids and ammonothermally grown crystals.

**Figure 7:**
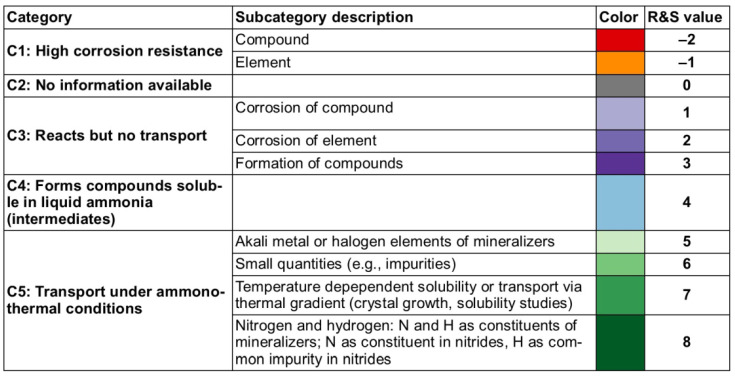
Data visualization via color-coding legend describing the qualitative reactivity and solubility information on the periodic table. The first column describes the main categories, while the second column defines subcategories where necessary. The third column indicates the color that will be assigned in the periodic table, while the last column lists the reactivity and solubility (R and S) values assigned for plotting and listing purposes.

For a clear visual representation of the data, a color-coded legend will be utilized. In the periodic tables, each subcategory will be represented by a color, which is assigned according to the legend shown in Figure 7.

**Figure 8:**
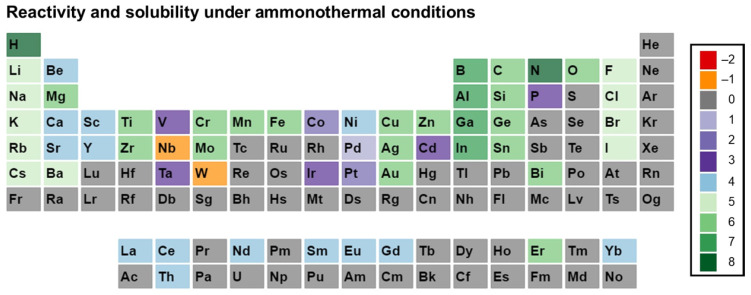
Color-coded periodic table illustrating the reactivity and solubility of elements split into different categories based on their interaction, regardless of the type of ammonothermal environment. For a definition of the categories listed in the legend, see Figure 7.

**Figure 9:**
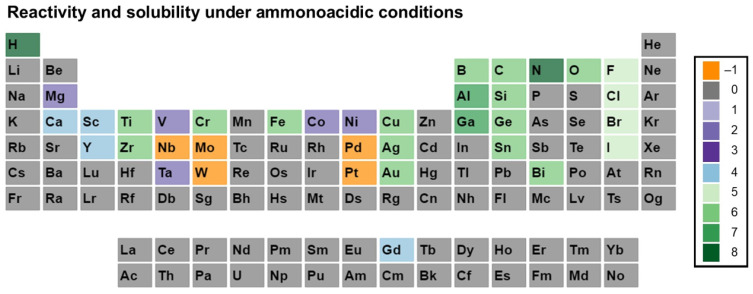
Color-coded periodic table illustrating the reactivity and solubility of known elements split into different categories based on their interaction specifically in ammonoacidic conditions. For a definition of the categories listed in the legend, see Figure 7.

**Figure 10:**
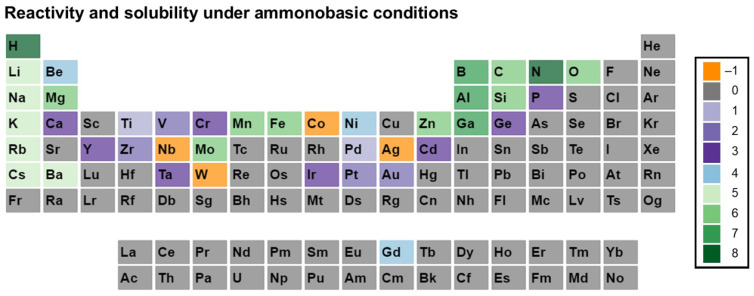
Color-coded periodic table illustrating the reactivity and solubility of known elements split into different categories based on their interaction specifically in ammonobasic conditions. For a definition of the categories listed in the legend, see Figure 7.

**Figure 11:**
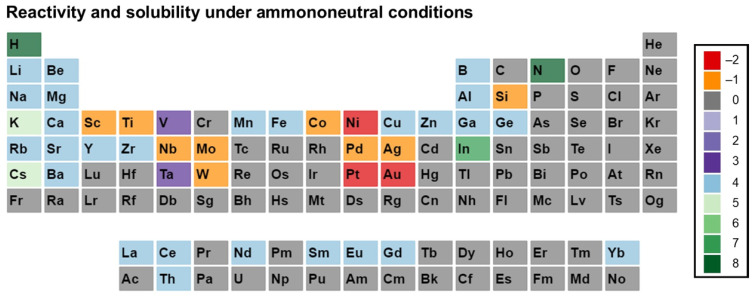
Color-coded periodic table illustrating the reactivity and solubility of known elements split into different categories based on their interaction specifically in ammononeutral conditions. For a definition of the categories listed in the legend, see Figure 7.

Color-coded periodic tables were devised to visually represent the reactivity and solubility of known elements within the ammonothermal environment in general (Figure 8), as well as under ammonoacidic (Figure 9), ammonobasic (Figure 10), and ammononeutral (Figure 11) conditions, respectively. In case of multiple values per element (commonly due to the availability of data for the use of different ammonoacidic/ammonobasic mineralizers, data for both elemental and compound samples, or deviating results), the highest value is displayed for that element. The complete set of data and references can be found in Appendix A (ammonoacidic), Appendix B (ammonobasic), and Appendix C (ammononeutral).

This systematic approach provides a consistent overview of both data availability and the feasibility of achieving reactions and/or solubility under the respective ammonothermal conditions. To indicate clearly for which elements there is a complete lack of data, the first periodic table (Figure 8) summarizes the data from acidic, basic, and neutral milieus.

Quite a few elements belonging to actinide and lanthanide series, and also to the last periods of the table, were not employed in the ammonothermal environment (no data available, grey). Considering the recent studies on the enhancement of the piezoelectric modulus of AlN by alloying with certain transition metals, knowledge on further transition metals, particularly Zr and Hf, would be of interest (based on Figure 2 and the references therein). In addition, for elements with data only on reactivity, but not on transport, knowledge of solubility in the sense of proven transport via an ammonothermal solution would be valuable, particularly for those for which applications are on the horizon. This applies to Be, Mg, and Si as dopants for III-nitrides, to the transition metals Sc, Ti, Y, Zr, Nb, Hf, and Ta for piezoelectric Al_1−*x*_TM*_x_*N (TM = transition metal), to Mg, Si, Mn, Zn as constituents of II-IV-N_2_ semiconductors (based on Figure 3 and the references therein), and to Ti, V, Zr, Nb, Mo, Hf, Ta, and W, as they are constituents of nitride superconductors (based on Figure 1 and the references therein). It should be noted that reactivity and solubility vary greatly with the used temperature and pressure conditions and mineralizers. Therefore, the observation of corrosion resistance under a limited set of ammonothermal conditions does not necessarily imply that reactivity and solubility cannot be achieved under different ammonothermal conditions and used mineralizers.

In the following, environment-specific information will be given on the basis of the following types of environments:

- Ammonoacidic conditions: acidic mineralizers such as the ammonium halides NH_4_X (X = F, Cl, Br, I) are present, enhancing the concentration of NH_4_^+^ ions;
- Ammonobasic conditions: basic mineralizers such as alkali and alkaline earth metals or amides or azides thereof are present, enhancing the concentration of NH_2_^−^ ions;
- Ammononeutral conditions: the concentration of NH_4_^+^ ions and NH_2_^−^ ions is balanced, either in supercritical ammonia without mineralizers, or due to the balanced presence of different compounds increasing the concentrations of the NH_4_^+^ ions, as well as that of the NH_2_^−^ ions, resulting in an ammononeutral milieu.

### 3.1. Ammonoacidic Conditions

As ammonoacidic conditions mean having mineralizers with halogens, these elements, alongside nitrogen and hydrogen, get transported as they are constituents of the solvent i.e., supercritical NH_3_ [103,153,154,155].

Ag showed varied responses in ammonoacidic conditions based on the mineralizer used. It is corrosion resistant with NH_4_F [156], whereas it gets corroded with NH_4_Cl [147]. It is reported to be transported in small quantities with NH_4_Br [157]. Ca was reported to form soluble intermediates in the ammonoacidic system with NH_4_F as the mineralizer [158].

Al gets dissolved and transported and crystallizes as AlN in the case of NH_4_Cl and NH_4_I [159,160], however, soluble intermediates were formed only when NH_4_F [161] and NH_4_I [162] were present as educts. Other group III elements like Ga form crystals in the presence of all mineralizers [150,154] and B was transported in the presence of NH_4_F, NH_4_Cl and NH_4_Br [163], at a near-ammonoacidic process. These nitrides will be discussed in the further sections. The group III-nitrides will be discussed in more detail in Section 4.1. Fe [87,164] and Cr [164] were reported to be transported as impurities into the system with NH_4_Cl as the mineralizer.

Au was seen to be resistant against corrosion with NH_4_Cl [147], but gets transported in the presence of NH_4_Br [157]. Bi and Cu both get transported as impurities when reacted in the presence of NH_4_Br [157]. Co was reported to be corroded in its elemental form, but remained stable as an alloy, Co_80_W_10_Al_9.4_ with NH_4_Cl [147]. C was found to be transported as an impurity with NH_4_Cl as the mineralizer [87,164], similar to that of O [87,103] with all the possible acidic mineralizers.

Gd was observed to be forming soluble intermediates, whilst Ge got transported in little quantities [157]. Mg seemed to be corroded in compound/elemental form [147], but also reacts to form compounds [119].

Mo, Nb, Pd, and Pt all were reported to be corrosion resistant (with Mo and Pt even in alloy forms) when NH_4_Cl was the mineralizer [147], with Nb losing its mechanical stability; on the other hand, Ni was observed to be corroded without getting transported [165,166], while maintaining its chemical passivity as an alloy [147].

For both Sc [167] and Y [168], soluble intermediates with NH_4_I were reported, while Y also gets corroded like Ta in the presence of NH_4_Cl [147]. Ti and V were seen to be corroded superficially, forming respective nitrides flaking off the surface [147]. Si, Zr, and W (in alloy form also) were described as showing high corrosion resistance with NH_4_Cl, but in similar conditions, Zr was observed to be corroded [147]. Si was also observed to be transported in the ammonoacidic system during experimentation with all the acidic mineralizers [103]. Sn was reported to be transported in smaller quantities with NH_4_Br as the mineralizer [157]. Also, Ti and Zr both were found as impurities when NH_4_Cl was used as the mineralizer [164].

### 3.2. Ammonobasic Conditions

For ammonobasic conditions, elements like Li [169], Na [102], K [170], Rb, and Cs, and [171] also occasionally Ba [172], apart from N and H, get transported into the ammonothermal crystal.

Ag was found to be showing corrosion resistance with NaNH_2_, while the Au sample was seen to be corroded, turning black [147]. Al was reportedly dissolved and transported and formed an AlN crystal with pure Li and K, LiNH_2_, KNH_2_, and KN_3_ [169,173,174]. B also gets transported via the system to form BN [111].

Be was reported form soluble intermediates [175] with NaN_3_ as the mineralizer, while C was known to be transported as an impurity with both NaNH_2_ [145] and KNH_2_ [176]. Ca formed compounds which were not reported to be transported in cases of NaN_3_ [177] and LiNH_2_ [178], whereas Cd [119] and Cr react similarly [179] in a KNH_2_ environment. Co shows higher corrosion resistance in a NaNH_2_ environment in both elemental and alloy forms [147]. Mg and Si were both observed to be transported through the system when reacted with metallic Na [87,140].

Ga formed bulk ammonothermal crystals when synthesized in ammonobasic conditions with NaNH_2_ [149] and KNH_2_ [109] serving as mineralizers. Gd was reported to form soluble intermediates [167] with NaNH_2_, whereas Ge formed compounds with no traceable transport with LiNH_2_, NaNH_2_, and KNH_2_ [42], apart from being corroded [147]. Fe was also observed as an impurity in the ammonobasic environment [87,140].

Ir was also reported to be corroded [147] in the presence of NaNH_2_. Mg was not only found to be forming soluble intermediates in the solution with NaNH_2_ [180], NaN_3_, and KNH_2_ [181], but corrosion studies were also performed in ammonobasic conditions with both compound and elemental forms [147].

Mn was reported to form soluble intermediates in supercritical ammonia with NaNH_2_ [182], apart from being incorporated into the crystal [183]. Mo (also as an alloy) showed strong corrosion resistance in a similar environment [147], but was found to be transported into the system upon reacting with metallic Na [87,140]. Ni and Nb were both observed to be strong against corrosion, with the latter losing its mechanical stability in the process, besides being inert as an alloy [147]. Ni was also reported to form a nitride in the presence of NaNH_2_ [184]. O was found to be transported as an impurity in both cases involving NaNH_2_ and KNH_2_ [145,176]. P was reported to form compounds in the presence of NaN_3_, without being transported across the system [185].

Pd and Pt were found to become corroded in the ammonobasic system, with the former in elemental form and the latter as an alloy [147]. Si was observed to form soluble compounds in the presence of KNH_2_ [119], while being vulnerable to corrosion with NaNH_2_ [147]. Ta was found to form compounds with no known transport process with Na, KN_3_, and RbNH_2_ [186].

Ti and V were both seen to be ineffective against corrosion, with a crack found in the V sample [147]. W was observed as having resistance to corrosion, even in its alloy form, while Y and Zr became heavily corroded [147]. Y was also reported to form compounds in the presence of KNH_2_ [187], while Zn was reported to form only soluble intermediates [188], but it is also as used as a dopant [189].

### 3.3. Ammononeutral Conditions

Al [190], Be [191], and Ce [192] were all reported to form soluble intermediates in ammononeutral conditions (liq. NH_3_), whereas Au and Co were chemically stable in their alloy forms, with the latter being stable in elemental form, too [147]. Cu forms similar compounds, but with CuCl and KNH_2_ in a 1:3 ratio, making it an ammonobasic milieu [193]. Ba was found to form soluble compounds with the presence of a secondary cation like Cs [194], K, and Rb [195].

Similarly, Ca also forms similar compounds with Na [196], K [197], Rb [198], and Cs [199] as secondary cations. Cs and K both act as mineralizers in the case of InN crystal growth, with InCl_3_ and KNH_2_ in a 1:3 ratio [141] or InI_3_ and CsNH_2_ in a 1:3 ratio [200], respectively.

Eu [201], Fe [202], Ga [133], Ge [147], and Gd [203] all form soluble intermediates with supercritical ammonia without any mineralizer present. La [204], Mg [205], and Nd [192] were found to form compounds which were soluble in ammononeutral conditions. Elements like Mo, Ni, Pd, Pt, Si, and W showed inertness to the ammononeutral environment in their respective alloy forms (with Mo, Pd, Si, and W being passive even in elemental form, too) [147]. Li forms soluble intermediates with K [206], whereas Mg forms with Cs [207], K, and Rb [208] as secondary cations in the ammononeutral environment.

Nb and Sc are corrosion resistant [147] in the ammononeutral environment, whereas elements like Sm [209], Th [181], Y [168], Yb [210], Zn [211], and Zr [212] formed soluble compounds. Sr was reported to form soluble intermediates with Rb [195], Cs [199], Na, and K [196] as secondary cations in respective compounds. Ta and V were found to form their respective nitrides, with no information being available regarding their transport [147].

In summary, many elements have been identified to react with the ammonothermal environment, leading to their dissolution and compound formation in basic and acidic environments. Of the two, the acidic environment is more reactive, offering more opportunities for crystal growth at the expense of added complexity to the growth system. The basic environment is less reactive, facilitating the design of these growth systems, though it may in turn limit the type of materials that can be grown from these solutions. Knowledge of the solubility of species in solution and the resulting dependence on temperature (and pressure) are important, as this enables the targeted pursuit of crystal growth using these elements as constituents of the growing nitride crystal. Depending on the degree of solubility, it is possible to dope a material at low concentrations to modify its electrical conductivity, or to form alloys/compounds at higher concentrations. The following section explores the current state of ammonothermal synthesis and crystal growth for various compounds.

## 4. Synthesis and Crystal Growth of Nitrides

In the following sections, the application of the ammonothermal method for the growth of nitride crystals will be reviewed. The first subsection will focus on the binary and ternary III-nitrides, whereas the second subsection is dedicated to other nitrides obtained by ammonothermal crystallization until now.

### 4.1. III-Nitrides

This section covers the binary III-nitrides BN, AlN, GaN, and InN, as well as the ternary alloy AlGaN. All subsections give a review of the current state of ammonothermal growth of these nitrides, except for the GaN section. The ammonothermal field of crystal growth is mainly investigated for GaN, contrary to other nitrides. Since there are already several review articles on ammonothermal GaN, this review provides a very compact overview and guides the reader towards the other reviews. For the same reason, we start with GaN before moving on to materials for which ammonothermal growth is less well-investigated.

#### 4.1.1. GaN (Overview of Available Literature)

Many reviews and perspectives have targeted the ammonothermal growth of bulk GaN single crystals [81,86,87,98,99,100,105,128,142,213,214,215,216]. Therefore, this section provides an overview of these reviews and perspectives from 2015–2023. However, a very brief summary of the state of the art will be given beforehand.

State-of-the-art ammonothermal GaN crystals (boules) have reached the following characteristic values (reported from 2018–2023): a boule thickness of 1–4 mm [86], a bowing radius of 20–1460 m [86], a threading dislocation density of 10^3–5^ × 10^4^ cm^2^ [86], a rocking curve width of up to 11.9–19.2 arcsec (reflection: (0002)/slit size: 2 mm × 1 mm) [84], and a wafer size of up to 100 mm [86]. Information on the process conditions of ammonothermal GaN growth is referred to in Section 2.

The previously published reviews and perspective articles on the ammonothermal growth of bulk GaN are listed in Table 2, alongside their focus areas. In Table 2, the term “ammonothermal scope of the article” shows the number of pages in the publication (reference pages are excluded) that cover the ammonothermal growth of GaN. This value serves as an indicator for the depth of discussion. The topics in Table 2 are grouped into four blocks: miscellaneous, crystal properties, growth conditions, and growth technology. All of the articles with a lower ammonothermal scope value (less than eight pages) have their focus on the general description of the process, or the general part of the growth technology. These articles help to quickly get an overview, whereas the others provide more detailed information.

It is necessary to point out that the focus of the publications did not change for the blocks “crystal properties” and “miscellaneous” from 2015–2023. On the contrary, the focus changed for the blocks “growth conditions” and “growth technology”. Over the last eight years, the focus of “growth technology” increased, and the focus of “growth conditions” decreased. This change indicates the shift of the development progress from basic research towards a more commercially usable process, where more focus is put on growth technologies. This observation fits to the assessment of the near-equilibrium ammonothermal (NEAT) method of SiX-Point to a technology readiness level (TRL) of six, which stands for a technology which is near to industrial deployment [217]. Although the process shows a TRL of six, recent ongoing research shows a lot of unknowns in the material system and basic research is still needed, for example, the micro and macro defects in ammonothermal GaN newly identified by L. Kirste et al. [107,218].

Besides that, in 2021 a book was published by R. Niewa and M. Meißner which covers the ammonothermal growth of GaN and further nitride materials, autoclave technology for crystal growth and in-situ monitoring, and the chemistry of ammonothermal growth of GaN and related materials. So far, it is the only book that exclusively looks at the ammonothermal process [219].

#### 4.1.2. AlN

In comparison to the ammonothermal growth of GaN, the ammonothermal growth of AlN has not been heavily reported. The primary method for growing bulk AlN single crystals is the PVT method, which has demonstrated to yield high-quality, free-standing AlN wafers [85]. This section reviews the ammonothermal method for AlN growth by offering a historical view and a detailed discussion of the field.

The first ammonothermal synthesis of AlN was reported in 1988 by D. Peters [38]. The last scientific publication about ammonothermal synthesis of AlN was reported by B. T. Adekore et al. in 2006 [174]. To evaluate to which extent the ammonothermal growth of AlN has been explored, all authors, publications, countries, or institutes that published experimental results on ammonothermal AlN growth (original research papers) are listed chronologically:

- D. Peters, 1988 [160]/D. Peters, 1990 [173], Germany, FuE Chemikalien, Hoechst AG, Werk Knapsack, Hürth;
- R. Dwiliński et al. 1998 [169]/R. Dwiliński et al. 1998 [220], Poland, Warsaw University, Institute of Experimental Physics;
- Y. C. Lan et al. 1999 [159]/Y. G. Cao et al. 2000 [221], Y. G. Cao et. al. 2001 [222], China, Institute of Physics, Centre for Condensed Matter Physics, Chinese Academy of Sciences, Beijing;
- A. I. Motchanyy et al. 2005 [223], Russia, Russian Research Institute for the Synthesis of Materials (VNIISIMS);
- B. T. Adekore et al. 2006 [174], USA, NCSU, Department of Materials Science and Engineering.

In addition to these, there exists a patent from Dwiliński et al. from 2008 [224]. There is one perspective paper which briefly touches on the ammonothermal synthesis of AlN [225] and one review paper that focuses on intermediates in the ammonothermal system in general and touches AlN slightly [119].

The list indicates that a global interest in ammonothermal growth of AlN existed from 1988 until 2008. However, nothing has been published within the last 16 years. Obviously, the global interest in growing ammonothermal AlN has leveled down, likely due to the faster success of PVT growth in the case of AlN, including recent breakthroughs in diameter enlargement [83].

All publications about the ammonothermal synthesis/crystal growth of AlN primarily report on the solubility of Al and characterization of synthesized AlN crystalline powder/grains, which will be discussed below. For the following discussion, the ratio [X:X:X] always stands for the ratio [nutrient:mineralizer:ammonia] and all mole ratios of D. Peters [173] were numerically solved from his experimental data.

##### Solubility Results Will Be Discussed in the Following

There are two types of nutrients mentioned in the literature, Al and AlN.

**Al:** D. Peters [173] demonstrated that it is possible to dissolve Al in pure ammonia, using Al-powder as the nutrient. An Al to AlN conversion of 32% could be reached, using Al-powder, the ratio [1:0:7.9], at 500 °C and 200 MPa, though it is likely insufficient to pursue crystal growth. As such, a mineralizer is required to dissolve a significant amount of Al, similarly to Ga or GaN solubility. A summary of conditions under which Al is dissolved in ammonothermal environments is provided in Table 3, together with a description of the crystalline products obtained. Unlike GaN [70], irrespective of growth chemistry, only retrograde solubility has thus far been observed for AlN [160,173,174].

D. Peters [173] investigated the conversion of Al to AlN (Al–AlN conversion) in ammonia + mineralizer solutions, in relation to ammonia decomposition, ammonia to mineralizer ratio, and temperature.

He observed an ammonia decomposition dependent on the Al–AlN conversion. At an ammonia decomposition ≥ 40%, a complete Al–AlN conversion was possible, using an Al:mineralizer ratio of 1:0.09–0.13 at 600 °C and 200 MPa.

The ammonia decomposition depends mainly on the pressure/amount of ammonia. The higher the pressure/amount of ammonia, the lower the decomposition rate. However, the ammonia decomposition also depends on the deployed amount of mineralizer (KNH_2_). According to the reaction of KNH_2_ + 3NH_3_ + Al to KAl(NH_2_)_4_ + 1.5H_2_, a higher amount of KNH_2_ leads to a higher decomposition of ammonia. D. Peters [173] based his statements on the following amount of ammonia and mineralizer dependent experiments. Firstly, he increased the Al to ammonia ratio from 1:1.85 to 1:4.4 (at a constant Al to mineralizer ratio of 1:0.09 at 600 °C), which resulted in an increase of the Al–AlN conversion from 40% to 100%. The ammonia filling levels caused a pressure increase from ca. 1 MPa to 2 MPa. Secondly, he increased the Al to mineralizer ratio from 1:0.09 to 1:0.1 (at a constant Al to ammonia ratio of 1:7.9 at 600 °C), which resulted in a decrease of the Al–AlN conversion from 100% to 94%. Accordingly, a peak Al–AlN conversion dependent on the mole ratio of mineralizer (KNH_2_) was observed by D. Peters [173], contrary to Ga or GaN. The change in the Al–AlN conversion can be explained by an increase in the solubility, but also by a change in the crystal growth (transport, formation, or dissolution of the intermediate and growth kinetics).

In addition, D. Peters [173] measured an increase in the Al–AlN conversion with a temperature increase from 500 °C to 600 °C, at 200 MPa.

**AlN:** The solubility of AlN is less investigated than that of Al. B. T. Adekore et al. [174] tried unsuccessfully to dissolve AlN source material, using KN_3_ as a mineralizer with the ratio [1:n/a:19.6] at 550 °C and 246–286 MPa. Y. G. Cao et al. [222] successfully dissolved AlN source material using NH_4_Cl as mineralizer at 580 °C and probably at above 253 MPa (no AlN:mineralizer:ammonia ratios were given). This could point towards a solubility difference between the acidic and basic system, but more data are needed.

##### Crystallization Results Will Be Discussed in the Following

Every publication in which ammonothermal dissolution of Al or AlN was reported also reported the crystallization of AlN from the respective solution. The crystalline products obtained are listed in Table 3. Within all the process conditions listed in Table 3, only B. T. Adekore et al. [174] approached a seeded crystal growth. The other authors reported the crystallization of layers, grains, or powders. In the following, we discuss the crystallization of AlN with regards to the obtained largest crystalline products, growth environment, fluid flow, and the influence of the solubility on the obtained crystalline products.

**Largest crystalline products:** To evaluate the current state of crystalline ammonothermal AlN, a comparison of the crystallization results from D. Peters [173] and B. T. Adekore et al. [174] is useful, as both crystallized a layer in the mm-range and reported it in detail. B. T. Adekore et al. [174] demonstrated an ammonothermal growth of a 100-µm-thick, dense, contiguous polycrystalline AlN-layer on the Ga-polar side of a GaN seed (HVPE) and a less dense, 1.5 mm thick, polycrystalline AlN-layer on the N-polar side, grown within 21 days. D. Peters [173] achieved an AlN-layer up to some millimeters in thickness (probably on the inner autoclave wall), which consisted of closely packed, completely parallel columns grown in the <0001> direction (Al-polar side), within a duration of app. 2.7–4.6 days.

Both D. Peters [173] and B. T. Adekore et al. [174] observed the growth of a lower structural quality layer prior to the establishment of stable growth conditions. This initially grown layer could explain why the seeded growth attempt of B. T. Adekore et al. did not lead to a single crystalline layer.

**Growth environment:** The growth zone temperatures that yielded crystallization in the basic and acidic system are stated in Table 3.

In the acidic system, Y. C. Lan et al. [159] produced their powder at 450 °C with an NH_4_Cl mineralizer. A. I. Motchanyy et al. [223] reported that the nucleation results below 450 °C were the worst with the NH_4_Cl mineralizer. Correspondingly, 450 °C is the lowest temperature at which noticeable nucleation of AlN has been observed in the acidic environment. The highest reported crystallization temperatures in the acidic environment were reported by D. Peters [160] at 600 °C with a NH_4_I mineralizer. Accordingly, the temperature range where crystallization occurred in the acidic system can be delineated between 450 °C and 600 °C.

In the basic system, D. Peters [173] quoted that the temperature in the growth region has to exceed 400 °C with KNH_2_ as mineralizer. R. Dwiliński et al. [220] produced their powder at T < 500 °C with Li, K, and LiNH_2_ as mineralizers. Correspondingly, the T-range 400–500 °C is the lower T-range in which the nucleation of AlN can be expected. The highest reported temperature for the precipitation in the basic environment is 600 °C with KNH_2_ as mineralizer [160,173]. Accordingly, the temperature range where crystallization can be expected in the basic system can be delineated between 400 °C and 600 °C.

The crystallization temperature range in the acidic and basic environment does not seem to differ significantly. The crystallization temperature range of ammonothermal AlN seems comparable to the crystallization temperature range of GaN, though according to Y. G. Cao et al. [222], the growth temperature for AlN is 80 °C higher than GaN.

**Fluid flow:** Besides temperature, pressure, and mineralizer, the flow velocities of the supercritical fluid are also thought to be of importance. D. Peters [173] suggested and B. T. Adekore et al. [174] simulated that lower flow velocities of the supercritical fluid would be beneficial for crystal growth. This is in accordance with recent investigations on the effects of fluid flow velocity on the quality of ammonothermally grown GaN bulk crystals [138].

**Influence of the solubility to the obtained crystalline products:** Subsequent studies after that of D. Peters [173] had lower crystallization rates in their growth experiments. A possible explanation for this, is that all groups completely dissolved their Al nutrient. Accordingly, these groups used too low Al nutrient amounts, or too high ammonia or mineralizer amounts. From the results of D. Peters [173] a border ratio can be derived at which the Al to AlN conversion starts to decrease below 100% at 600 °C. This ratio is 1:0.09:4.4. Below this ratio, aluminum does not completely react to AlN. With this growth condition, the growth of ammonothermal AlN single crystals should be more feasible. However, there is a tradeoff, as a decreasing amount of mineralizer also increases the reaction time and decreases the crystallization rate [173].

Accordingly, it seems that ammonothermal AlN growth requires a lower amount of mineralizer dissolved in NH_3_, in relation to ammonothermal GaN growth. In comparison, the Na mineralizer: NH_3_ ratio which is used for GaN growth is 0.05 at 575 °C [140].

#### 4.1.3. AlGaN

The ammonothermal growth of bulk Al*_x_*Ga_1-*x*_N is less explored, with only two literature reports [222,226]. Currently, no primary method for growing bulk Al_x_Ga_1−x_N exists, though approaches to grow bulk Al*_x_*Ga_1−*x*_N with other methods like HVPE exist [227].

Via the acidic ammonothermal method, Y. G. Cao et al. [222] grew polycrystalline Al*_x_*Ga_1−*x*_N fragments with an average thickness of 600 µm. The effective growth time was 5 days, using NH_4_Cl as mineralizer, holding at a pressure of above 152–253 MPa and a temperature of 500-580°C. In their experiments, Y.G. Cao et al. [222] observed that the solubility of Al*_x_*Ga_1−*x*_N depends both on the mineralizer concentration and the Al content. Additionally, Y.G. Cao et al. identified the need to use an Al*_x_*Ga_1−*x*_ alloy as feedstock (as opposed to pure Ga and Al metals) to successfully synthesize polycrystalline Al*_x_*Ga_1−*x*_N over the entire Al content range (Al: 0%/15%/30%/50%/60%/70%/80%/90%/100%). Regardless of the process conditions used, Y.G. Cao et al. did not succeed in growing Al*_x_*Ga_1−*x*_N if Ga and Al were used as feedstock.

Via the basic ammonothermal method, Dwiliński et al. [226] grew a 10 µm thick single crystalline layer of Al_0.2_Ga_0.8_N on single crystalline GaN. The effective growth time was 2 days, using NaN_3_ as a mineralizer, holding at a pressure of 360 MPa and a temperature of 550 °C. Contrary to Y.G. Cao et al. [222], R. Dwiliński et al. [226] used Ga and AlN as feedstock materials.

Overall, these results clearly demonstrate that ammonothermal growth of crystalline Al*_x_*Ga_1−*x*_N is feasible for the entire range of stoichiometries. However, the feasibility of bulk single crystal growth of Al*_x_*Ga_1−*x*_N via the ammonothermal method is yet to be proven.

#### 4.1.4. InN

Synthesis of indium nitride (InN) in an ammonothermal environment was first reported in 2018 by J. Hertrampf et al. [141]. The material is of interest as a fast switching, relatively narrow bandgap semiconductor. Given its low decomposition temperature, synthesis has been a major challenge with any method. One notable aspect of the experimental procedure is the use of both acidic and basic mineralizers to produce a compensated ammonia environment closer to neutral in pH. A mixture of InCl_3_ and KNH_2_ at ratios between 0.4:1 and 0.25:1 (or between 1.2:1 and 1:0.75 in terms of Cl:K) was used at temperatures ranging from 663–723 K. Liners of either silicon nitride or boron nitride were also included inside the autoclave to help protect the walls from infiltration by indium. Pressures of operation went as high as 300 MPa, while reaction times ranged from 10 to 120 h. At 773 K, aggregates of faceted ball-shaped crystallites were found at a ~400 nm length scale after 24 h in a BN liner, while rods up to 5 microns long were produced from a Si_3_N_4_ liner within the same time. At 663K, well-formed platelets reached over 2 microns in length after 10 h in a silicon nitride (Si_3_N_4_) liner, while chunky cylinder agglomerates of about the same size were observed after 100 h. Longer periods of time only resulted in decomposition of the InN into metallic indium spheres. As a result of these demonstrations, a path to larger crystals based on these conditions is not evident, however the first demonstration of growth was a milestone. It was found that excess quantities of either mineralizer species resulted in at least some metallic indium being formed.

A follow-up contribution from the Stuttgart group [144] investigated the intermediate molecules involved in dissolution, transport, and crystal growth of ammonothermal InN. With a combination of powder diffraction and vibrational spectroscopy, they describe the properties of [In(NH_3_)_5_Cl]Cl_2_ and InF_2_(NH_2_). Both reaction products were found in the bottom of the autoclave (the hottest part), from which the authors infer a retrograde solubility in the system when using operating temperatures of 663–773 K.

In 2022, P. Becker and R. Niewa continue to focus on the ammononeutral regime but move to a different combination of mineralizers: InI_3_ and CsNH_2_ [200]. The researchers also transition to a different pressure and temperature regime with 34 MPa and only 423 K, presumably to avoid decomposition of newly grown InN. Cs is referenced to be about twice as soluble in liquid ammonia, and heavier halides are also generally observed to be more soluble in ammonia [228]. The products of the ammonothermal reaction were InN and CsI, but the particle sizes were smaller and less faceted than those obtained in the syntheses at higher temperatures of base and acid ions, but move to a different combination of mineralizers: InI_3_ and CsNH_2_ [200]. Although the morphology is disappointing, the ability to synthesize materials ammonothermally at such low temperatures and pressures is encouraging for the growth of new nitrides.

The second half of this paper looks to investigate the separate contributions of KNH_2_ and ICl_3_, returning to the pressure and temperature space of the original paper (250 MPa, 663 K). By spatially separating the two mineralizer compounds, placing the one in the crucible and the other below the crucible at the bottom of the autoclave, they were able to demonstrate that the KNH_2_ is completely dissolved in the ammonia while synthesis happens close to the ICl_3_. This remains true when the locations of the two materials are swapped. When the indium chloride was at the bottom of the autoclave, InN and KCl were found, but when it was in the crucible, only the intermediate [In(NH_3_)_5_Cl]Cl_2_ was found. It would seem, then, that the mobility of InCl_3_ and the intermediate in the autoclave is relatively small.

#### 4.1.5. BN

Both cubic and hexagonal boron nitride have become materials of great interest in the device research community [229]. Cubic boron nitride (in zincblende structure, termed $\bar{4}3m$-BN in this review for consistency but often termed c-BN) has been demonstrated as mm-scale single crystals, derived from the high pressure high temperature (HPHT) synthesis method at temperatures from 1200–1800 K and pressures ranging from 2–7 GPa [230]. It is an UWBG semiconductor with attractive predicted properties for high power switching [231]. Hexagonal boron nitride (6/mmm-BN, usually termed h-BN) is formed of 2D graphene-structured layers held together by van der Waals forces [232]. While both graphene and 6/mmm-BN have high thermal conductivity, unlike graphene, 6/mmm-BN is electrically insulating, making it a useful complement for potential integrated 2D devices [233]. Neither material can currently be synthesized as single crystals in the bulk at centimeter or greater length scales. Because of the success of ammonothermal growth for other nitrides, BN is thought to be a candidate for ammonothermal synthesis, although relatively little published work is available in this direction.

The earliest reported successful attempt is a 2018 paper by Maruyama et al. [234]. Using both Ba_3_N_2_ and MgB_2_, along with hexagonal boron nitride as educts, the authors demonstrate synthesis of both 6/mmm-BN and rhombohedral boron nitride ($\bar{3}$-BN, known as r-BN). With an autoclave filled with supercritical ammonia at 750 °C and 120 MPa, two synthesis conditions were tested, one with the Mg- and Ba-containing additives mixed with 6/mmm-BN and one with 6/mmm-BN alone. In the absence of the alkaline earth additives, no change to the 6/mmm-BN morphology was observed. With the additives, however, triangular features on the order of 100 nm were found and X-ray diffraction (XRD) was able to detect peaks unique to $\bar{3}$-BN. No estimate was made for the solubility of the materials in ammonia, nor was any mass transport necessarily involved, since products and reactants were in the same location.

In a 2023 paper, J. Dooley et al. report on the solubility of boron nitride in ammonia using sodium as a mineralizer [111]. The experiments involved $\bar{4}3m$-BN feedstock placed in a crucible inside an autoclave. The reactor conditions varied from 450–600 °C and 150–190 MPa with 0.2 mol of $\bar{4}3m$-BN, 0.00 to 0.53 mol/L of sodium, and 17.7 to 24.6 mol/L of ammonia. Holding times varied from 24 to 96 h. The data presented support a normal solubility behavior relative to temperature peaking at 0.05 mol_BN_/mol_NH3_ at a temperature of 600 °C. Full saturation was apparently achieved in less than 24 h, and solubility as a function of sodium content was essentially flat for sodium density higher than 0.08 mol_Na_/L. The solubility was calculated by comparing $\bar{4}3m$-BN mass in the crucible before and after the ammonothermal experiment, so it is evident that the dissolved BN species was mobile enough to move through the mesh cap of the crucible and deposit itself elsewhere in the autoclave. With both an exploitable solubility curve and nutrient species mobility established, some kind of BN growth is expected to be feasible. To date, however, the nature of such growth has yet to be reported.

Besides that, there exists a paper from 1979, which reports on a process near to the ammonoacidic process, the HPHT synthesis [163]. T. Kobayashi [163] converted 6/mmm-BN powder (1–5 µm in size) to $\bar{4}3m$-BN powder (0.2–1 µm in size) at 5.6 GPa, 1500 °C, in 30 min (process time), with four different mineralizers as solvents: NH_4_F, NH_4_Cl, NH_4_Br, and NH_4_I. But, contrary to the ammonothermal process, no ammonia was filled in. He observed that the gained conversion rate was dependent on the mineralizer used. The gained conversation rate decreased with an increase of the melting point of the mineralizer. Accordingly, the highest conversation rate has been reached with NH_4_F. That points towards ammonothermal comparable conditions and a transport of B in the fluid phase, because the conversion rate was dependent on the mineralizer.

### 4.2. Novel Nitrides and Related Materials beyond Group III-Nitrides

A variety of ternary and multinary nitrides have been obtained via variants of ammonothermal synthesis. Those materials are listed in Table 4, together with the main synthesis parameters used. From a crystal growth point of view, it is important to note that in most cases, the synthesis and transport mechanisms are not well understood, and the synthesis of microcrystalline powders does not necessarily imply an appreciable solubility of all constituents in supercritical ammonia with the mineralizer and process conditions applied. In the case of ZnGeN_2_, the presence of X-ray absorbing solutes likely representing Zn- and Ge-transporting intermediates has been observed via in situ X-ray imaging [42].

In summary, the crystal growth of a few binary nitrides, including GaN, AlN, InN, and BN and a few ternary nitrides has been demonstrated. Of these, GaN is most matured, while AlN, InN, and BN and all of the ternary nitrides are in their early stages of investigation and development. Given the current state of the art, opportunities remain for the exploration of novel nitride compounds of binary, ternary, or higher order. Additionally, looking beyond crystal growth in itself, the control over the functional properties (such as electrical conductivity, optical absorption, magnetic properties, etc.) is a fruitful area to obtain functional materials for use in a larger fabrication chain leading to functional devices (i.e., as bulk materials or substrates). Understanding and controlling defects that contribute to changes in material properties is important and will be discussed in the following section.

## 5. Generation of Point Defects and Their Impact on Crystal Properties

This section focuses on the effects of point defects (both native and impurity-related) on the properties of the ammonothermally grown crystals. More specifically, Section 5.1 and Section 5.2 refer to unintentional point defects/dopants. Section 5.3 refers to intentional point defects/dopants and their interaction with unintentional point defects/dopants, which in turn influence semiconductor properties.

Currently, it is possible to crystallize both n- (O as a dopant) and p-type (Mg as a dopant) conductive, as well as semi-insulating (SI) (Mg or Mn as a dopant) ammonothermal GaN. The mentioned dopant elements represent examples of the most commonly used ones for ammonothermal GaN.

The discussion in the respective sections is based exclusively on the data from ammonothermally grown GaN crystals, as the available information is insufficient for all other materials grown or synthesized ammonothermally thus far. Nevertheless, considering the unique characteristics of the ammonothermal growth environment, it can be assumed that some of the effects observed for GaN will also occur for some other nitrides grown with this method. These characteristics are the supercritical ammonia solution containing mineralizers, the growth temperature up to 600 °C, and pressure up to 450 MPa.

### 5.1. Native Point Defects and Related Defect Complexes

The most predominant native point defects in GaN are Ga–vacancies and their related complexes [105,240,241,242]. For GaN-growth using the basic ammonothermal environment, Ga–vacancies are found to be in the range of 10^18^–10^19^ cm^−3^ [240,241]. Recently, in 2024, M. Zając et al. [243] showed for the first time that N–vacancies form in basic ammonothermal GaN as well (specifically, in Mg-doped GaN), albeit only in small amounts in the order of 10^17^ cm^−3^. For GaN growth using the acidic ammonothermal environment, mainly Ga–vacancies (with few N–vacancies) are stated to be in the range of 3 × 10^16^–9 × 10^16^ cm^−3^, according to the recent publication by K. Shima et al. [242]. These Ga–vacancies and their related complexes are of interest for several reasons; they act as electron acceptors and hence compensate n-type doping and limit the carrier mobility, they contribute to sub-bandgap optical absorption via formation of near-bandedge states, and they even contribute to device degradation [105,240].

In the ammonothermal system, the Ga–vacancy complexes with H atoms (V_Ga_–H complexes) are the predominant complexes [240,244]. Hence, we focus on these defects in this section, which is primarily based on the reviews by S. Suihkonen et al. [87] and M. Zając et al. [105], but includes more recent advances as well. For considerations on the formation of defects, note that the ammonothermal system represents N-rich conditions. The charges state of the point defects will be stated using the “modified” Kröger–Vink notation [245].

Of all bulk crystal growth techniques for GaN, the ammonothermal technique yields GaN with the highest Ga–vacancy concentration. This is speculated to be due to the relatively low growth temperature [240]. According to F. Tuomisto [240], the formation mechanism of Ga–vacancies and their related complexes depends strongly on temperature, pressure, and chemistry of the different crystal growth processes. However, the amount of oxygen impurities has been identified as one of the main variables controlling Ga–vacancy formation; for all bulk GaN crystal growth techniques, the concentration of Ga–vacancies increases with increasing oxygen concentration [240].

Removal of a Ga atom from a Ga site in the GaN crystal lattice takes away three valence electrons and leaves behind four nitrogen dangling bonds, comprised mainly of the 2p orbitals of nitrogen [246]. This vacancy can have four different charge states from $V_{Ga}^{-3}$ till $V_{Ga}^{+1}$ [246]. All these charge states have very high formation energies and are unlikely to be formed ($V_{Ga}^{-3}$ has the lowest formation energy at the conduction band minimum (CBM) near 2 eV at N-rich conditions [247]). With a donor impurity complex, the formation energy of a Ga–vacancy can be considerably lowered [246]. For such a Ga–vacancy complex generation to happen, however, a donor impurity must be present in a high amount. In ammonothermal growth, hydrogen is such an impurity which can act as a donor, and it likely occurs in high quantities due to the ubiquity of hydrogen in the solvent ammonia. The lowered formation energy in combination with the hydrogen oversupply results in the mentioned formation of the predominant Ga–vacancy complexes with multiple H atoms [246,248]. It is hypothesized that hydrogenated Ga–vacancy complexes form by an incomplete dehydrogenation of ${NH}_{3}{,\;\mathrm{NH}}_{2}^{-1}$, or, ${\mathrm{NH}}_{4}^{+1}$ molecules [87]. This assumption is preceded by the assumption that N from NH_3_, ${\mathrm{NH}}_{2}^{-1}$, or, ${\mathrm{NH}}_{4}^{+1}$ is the nitrogen source of GaN [87].

According to the four nitrogen dangling bonds, four V_Ga_–H complexes are: (V_Ga_H_1_), (V_Ga_H_2_), (V_Ga_H_3_), and (V_Ga_H_4_). These V_Ga_–H complexes can have the following charge states: (V_Ga_H_1_)^0,−1,−2^, (V_Ga_H_2_)^0,−1^, (V_Ga_H_3_)^0^, and (V_Ga_H_4_)^+1^. According to first-principles calculations, V_Ga_H_1–2_ forms a deep acceptor about 0.8 eV above the valence band maximum (VBM) [87], and V_Ga_H_3_ is neutral and forms a shallow defect-state close to the VBM [87]. V_Ga_H_4_ would be the only complex that forms a donor defect-state in the valence band [87], but this was not detected so far.

**Figure 12:**
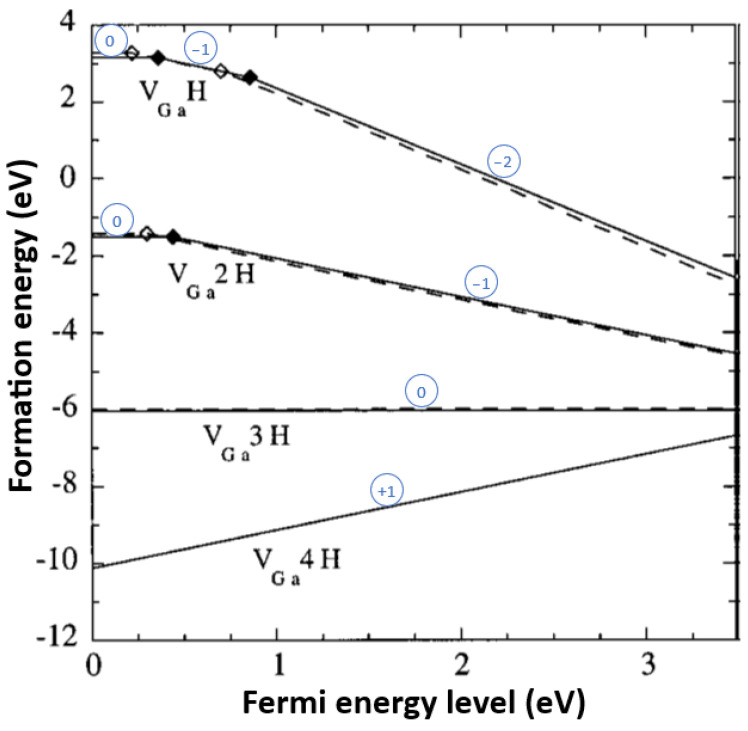
Formation energy as a function of FEL for hydrogenated Ga–vacancies for Ga-rich conditions of wurtzite–GaN, calculated via DFT. The FEL is defined to be 0 eV at the VBM and 3.48 eV at the CBM. Legend: blue circles the oxidation states of the complexes, diamond symbols: transitions of the oxidation stages, solid line: H atoms have a bonding to the N atom located along the c-axis, dashed line: H atoms have no bonding to the N atom located along the c-axis. Reprinted and modified from A. F. Wright 2001 [249] with permission, Copyright (2001) by the American Physical Society.

Which of the four V_Ga_–H complexes will be formed depends on the likelihood of multiple H atoms being available and the formation energy at the time of incorporation [248]. The formation energy depends on the Fermi energy level (FEL). Figure 12 shows the dependence of formation energy on the FEL for wurtzite–GaN at Ga-rich conditions, calculated from A. F. Wright [249]. The difference between Ga-rich and N-rich conditions is typically in the range of <1 eV, accordingly, the order of the formation energies of the V_Ga_–H complexes is not influenced. The order is: V_Ga_H_1_ > V_Ga_H_2_ > V_Ga_H_3_ > V_Ga_H_4_. But it is necessary to mention that according to the used functional and framework approximations, the formation energy order can change. C. E. Dreyer et al. [250] calculated the formation energies of V_Ga_H_1_, V_Ga_H_2_, V_Ga_H_3_ dependent on the FEL, also via DFT at N-rich conditions in the wurtzite structure, but they used other framework approximation. They have calculated for V_Ga_H_1_, V_Ga_H_2_, V_Ga_H_3_ the same formation energy order for an FEL near the VBM, but not near the CBM. For an FEL near the CBM, they have the following order: V_Ga_H_1_ > V_Ga_H_3_ > V_Ga_H_2_. M. A. Reshchikov et al. [251] calculated the formation energies of V_Ga_H_1_, V_Ga_H_2_, V_Ga_H_3,_ V_Ga_H_4_ via the Heyd–Scuseria–Ernzerhof hybrid density functional (HSE) at N-rich conditions, probably in the wurtzite structure and for an FEL near the CBM. Under these conditions, they have obtained the following order of formation energies: V_Ga_H_4_ > V_Ga_H_1_ > V_Ga_H_3_ > V_Ga_H_2_. How many H atoms (1–4) are available at the time of incorporation is difficult to determine, but if the complex formation takes place due to an incomplete dehydrogenation of NH_3_ (like that mentioned above), (V_Ga_H_1_), (V_Ga_H_2_) and (V_Ga_H_3_) can be expected to be formed.

To detect the type and quantity of complexes in GaN, the following two measurement techniques are used: Fourier transform infrared (FTIR) spectroscopy [241,252] and positron annihilation spectroscopy (PAS) [183,253,254,255]. Photoluminescence (PL) can also be used, but the technique needs more background information for the interpretation of the spectra [242,243,244,256]. According to the FTIR data compiled by M. Zając et al. [105] for the basic environment, the (V_Ga_H_3_) complex was always predominantly present, given an oxygen content ranging from 1 × 10^18^ cm^−3^ till 1 × 10^19^ cm^−3^. In addition to the (V_Ga_H_3_) complex, the (V_Ga_H_1,2_) were also present at oxygen concentrations > 10^18^ cm^−3^ (V_Ga_H_1,2_). The amount of (V_Ga_H_1,2_) increases with the oxygen amount in the GaN crystal [105]. Further, the (V_Ga_H_4_) complex was not observed and instead a complex containing O–H bonds was detected. The amount of this O–H bond complex also increased with an increasing amount of oxygen. Using FTIR, W. Jiang et al. [252] investigated GaN samples grown via an ammonoacidic process with varying oxygen content ranging from 1.6 × 10^18^ cm^−3^ to 5.1 × 10^18^ cm^−3^. In contrast to the basic environment, the (V_Ga_H_1,2_) complex was predominant and not the (V_Ga_H_3_) complex (interpretation of W. Jiang et al. [252] spectra with M. Zając et al. [105] Ga–vacancy absorption edges). Further, the amount of (V_Ga_H_1,2_) increased with an increasing oxygen concentration, like in the ammonobasic environment. Using PAS data from ammonoacidic GaN, S. F. Chichibu et al. [244] exclude the presence of V_Ga_–H complexes with multiple H atoms. According to the measured positron diffusion length, the (V_Ga_H_1_) complex appears to be the only possible V_Ga_–H complex.

These FTIR and PAS observations of the V_Ga_–H complexes in both the acidic and the basic environments has the following implications. The detection of the complexes (V_Ga_H_1_), (V_Ga_H_2_), and (V_Ga_H_3_), but not (V_Ga_H_4_), supports the hypothesis that Ga–vacancies are formed due to an incomplete dehydrogenation of NH_3_. In the ammonoacidic environment, the (V_Ga_H_1,2_) complexes are the dominant complexes. In the ammonobasic environment, the (V_Ga_H_3_) complex with more H atoms is the dominant complex. According to Figure 12, (V_Ga_H_3_) is more likely to be formed than (V_Ga_H_1,2_), provided that enough H atoms are available. Accordingly, in the ammonoacidic system, a lower amount of hydrogen is available at the time of incorporation, contrary to the ammonobasic system, assuming the process takes place near the thermodynamic equilibrium.

From the literature, it is not evident whether crystals grown in the acidic or the basic ammonothermal environment differ systematically in their Ga–vacancy concentrations. According to the following consideration, though, it can be speculated that acidic growth leads to a lower concentration of Ga–vacancies. According to F. Tuomisto [240], higher crystal growth temperatures lead to a lower number of Ga–vacancies, like it is typical for the acidic, in comparison to the basic, ammonothermal growth of GaN. Furthermore, higher oxygen concentrations lead to higher Ga–vacancy concentrations [240]. The mean oxygen concentration in the ammonoacidic system is lower than in the ammonobasic system, according to S. Suihkonen et al. [87]. In addition to that, recent publications indicate a similar trend [240,241,242] (see also the first paragraph of this chapter).

The concentration of V_Ga_–H complexes can be reduced with different effectiveness by post-growth annealing. Hydrogen is mobile in high-resistivity/p-type GaN at an annealing temperature > 500 °C [251]. The mobility depends on the charge state of hydrogen, $H_{i}^{-}$ and $H_{i}^{0}$ were found to be less mobile than $H_{i}^{+}$ [251,257,258]. This difference explains why the conditions that are effective for the removal of hydrogen differ by conductivity type as follows: 900–1000 °C at 1 atm under N_2_ atmosphere for p-type GaN, and at 1400 °C at 1 GPa under N_2_ atmosphere for n-type, respectively [251].

Before bonded hydrogen in a complex can diffuse, it must break free from the complex. 2016, in n-type (oxygen-doped) GaN, A. Uedono et al. [255] could not destroy Ga–vacancy complexes containing O or H by annealing at 1000 °C under N_2_ atmosphere, at an annealing time of 24 h. 2022 M. A. Reshchikov et al. [251] were able to reduce the amount of (V_Ga_H_3_) at an annealing temperature > 1000 °C, at a nitrogen pressure of 1 GPa and an annealing time of 1 h. Moreover, they observed that higher annealing temperatures result in a more efficient reduction of the (V_Ga_H_3_) concentration.

Ga–vacancies are known to be mobile at> 227–327 °C [259]. K. Horibuchi et al. [260] observed a “helical” deformation of a threading dislocation and the generation of voids in the GaN bulk material via heat treatment (1100 °C and atmospheric pressure). On the GaN substrate, a GaN layer was epitaxially deposited via MOCVD. This layer underwent the same thermal treatment and showed no “helical dislocations” and voids. They explained the generation of voids by the aggregation of vacancies, which deform the threading dislocation into a helical shape. F. Tuomisto et al. [261] showed that ammonothermal GaN has a higher number of Ga–vacancies than MOCVD GaN. This result supports the assumption of K. Horibuchi et al. [260] that the observed voids and the deformation of the threading dislocations are related to vacancies.

The current state of annealing of ammonothermal GaN is summarized in a paper by M. A. Reshchikov et al. [251].

Doping GaN using Mg has been found to strongly suppress the formation of Ga–vacancies [183]. It is possible to eliminate all the V_Ga_–H complexes in an Mg-doped oxygen-containing GaN crystal, due to an annealing process at 1100 °C under N_2_ atmosphere [105,183]. This effect is only detectable at Mg concentrations above 1 × 10^18^ cm^−3^ [105,183].

### 5.2. Impurities

The most cited measurement technique for impurities for ammonothermal GaN is SIMS (secondary ion mass spectrometry) [86,87,100,105].

One of the most successful measures implemented thus far for the reduction of impurity concentrations in ammonothermal GaN is the usage of noble corrosion-resistant metal (Ag/Pt) liners, which decreased the amount of most transition metals in the crystals to below the detection limit. For more details on this advancement, see Section 6.2. In this section, we focus mostly on discussing impurities that are incorporated in measurable quantities even with the use of a liner in the basic and/or acidic environment:

- H, O (major impurities) [87];
- C, Na, Mg, Al, Si, Mn, Fe, Zn (minor impurities) [87,105].

Zn is an exception, this element was not found in GaN crystals grown in an autoclave with a liner, but it was found in ammonobasic GaN crystals grown without the use of a liner [105].

This section discusses their concentration, contamination source, potential countermeasures, defect behavior, and their likelihood to form. Additionally, we investigate the effect of the crystallographic orientation of growing surfaces (Facet) and the growth time-dependent incorporation of impurities. Table 5 shows all previously mentioned impurities with their growth environment, concentration, and potential sources.

Note that the ammonothermal system represents N-rich conditions. Impurities that are incorporated at the Ga site are stabilized at N-rich conditions [264], whereas impurities which go on the N site are more stable at Ga-rich conditions. Accordingly, ammonothermal conditions favor the incorporation of those impurities that are incorporated on the Ga site. The lower the formation energy of an impurity, the more stable and likely it is to be incorporated in GaN. Note, the formation energies are calculated and mainly given in the publications in diagrams like Figure 12. The electronic state of impurities will be given according to the “modified” Kröger–Vink notation [245].

**Silicon**: Independently of the FEL, Si is stable on the Ga site at the charge state ${Si}_{Ga}^{+}$ [65,247] and forms a shallow donor 12–27 meV below the CBM [265]. All Ga–vacancy complexes have a higher formation energy than Si on the Ga site at the charge state ${Si}_{Ga}^{+}$ [247]. Only the neutral charge state of the Si_Ga_–C_N_ complex has a lower formation energy near the CBM [266].

**Aluminum**: Al forms a solid solution with Ga in Al*_x_*Ga_1-*x*_N (Section 4.1.3). Accordingly, Al has a good solubility in GaN and goes on the Ga site at the neutral charge state. J. H. Lee [267] grew Al-doped GaN via MOCVD and ascribed the reduced concentrations of electron scattering centers and non-radiative recombination centers to the Al doping. That also decreased the compensating acceptors, Ga–vacancies, and their related complexes [267].

**Magnesium**: Mg is stable on the Ga site and forms an acceptor level at 200 meV above the VBM [246,268]. According to J. L. Lyons et al. [268], Mg is an “accidentally” shallow acceptor, with a ${Mg}_{Ga}^{0}+e^{-}$ to ${Mg}_{Ga}^{-}$ transition for an FEL close to the VBM. At the neutral charge state, the hole is highly localized, contrary to a “normal” shallow acceptor where the hole is delocalized and loosely bonded [268]. In the immediate vicinity of the FEL to the VBM, the charge state ${Mg}_{Ga}^{+}$ is more stable than the neutral charge state at a formation energy of ca. 2 eV [268].

There are three theoretical studies which deal with different Mg complexes. The first study from J. Neugebauer and C. G. Van de Walle, published in 1995 [269], calculated the formation energy for Mg_Ga_–H complexes which had a very low formation energy and a neutral charge sate at <−3 eV. In the publication, there is no information whether they calculated it for Ga- or N-rich conditions. The second study published by J. L. Lyons and C. G. Van de Walle in 2017 [270] calculated the formation energy for Mg_Ga_–V_N_ complex. The complex had the lowest formation energy at the VBM near 0 eV and the charge state (Mg_Ga_–V_N_)^+2^ under N-rich conditions. D. Lee et al. [271] calculated the formation energy for M_Ga_–H_i_–V_N_ complexes, which had the lowest formation energy at −2 eV near the VBM and the charge state (Mg_Ga_–H_i_–V_N_)^+3^. In the publication, there is no information whether they calculated it for Ga- or N-rich conditions. Due to the oversupply of hydrogen in the ammonothermal process and the lowest formation energy of the complex Mg_Ga_–H, this complex will be the predominant form. However, every mentioned Mg complex introducing a state for an FEL near the VBM has a lower formation energy than Mg_Ga_. The Mg_Ga_–H complex is the dominant defect formed in GaN when growing in a hydrogen-containing environment. This defect is neutral with a deep level at 1.02 eV above the VBM [268]. As is now well known, this Mg_Ga_–H complex can be separated into H_i_ and Mg_Ga_ by annealing (Section 5.1).

**Si, Al, Mg group:** This group presents all metal impurities which are not transition metals and occur in GaN crystals despite the use of a liner. All of them, in their pure form, do not act as a deep level carrier trap [246,265,268]. Prospective methods for the reduction of their concentration will be discussed in the paragraph on oxygen (for contamination via polycrystalline GaN or mineralizer) and in the paragraph on the Mn, Fe, Zn group (for contamination via the inner autoclave wall), respectively.

**Iron:** Fe is stable on the Ga site at the charge states ${Fe}_{Ga}^{0}$, ${Fe}_{Ga}^{+}$, or ${Fe}_{Ga}^{-}$ and forms a deep acceptor 0.5 eV below the CBM [246]. The charge state ${Fe}_{Ga}^{0}$ is stable for an FEL in the middle of the bandgap [272]. For an FEL near the CBM, the charge state ${Fe}_{Ga}^{-}$ gets more stable and near the VBM, the charge state ${Fe}_{Ga}^{+}$ gets more stable [272]. With about 3 eV under N-rich conditions, the formation energy is relatively high [272]. For an FEL near the VBM, the complexes Fe_Ga_–V_N_, Fe_Ga_–H_i_, and Fe_Ga_–O_N_ are more stable than Fe_Ga_ [272]. Near the CBM, only Fe_Ga_–O_N_ is more stable [272]. All mentioned complexes act as deep defects in the bandgap [272]. Due to the high presence of all the complex partners H_i_ and O_N_ in the ammonothermal system, the formation of the complexes Fe_Ga_–H_i_ and Fe_Ga_–O_N_ is likely.

**Zinc:** Zn is stable on the Ga site at the charge states ${Zn}_{Ga}^{0}$, ${Zn}_{Ga}^{+}$, or ${Zn}_{Ga}^{-}$, and forms a deep acceptor 0.40–0.46 eV above the VBM [273,274]. The formation energy for an FEL near the VBM is relatively high at ca. 2 eV and decreases to −1 eV near the CBM under N-rich conditions [274].

**Manganese:** Mn is stable on the Ga site at the charge states ${Mn}_{Ga}^{0}$, ${Mn}_{Ga}^{+}$ or ${Mn}_{Ga}^{-}$ and forms a deep acceptor 1.5–1.8 eV above the VBM [105]. The charge state behavior is similar to Fe; the charge state ${Mn}_{Ga}^{0}$ is stable for an FEL, in the middle of the bandgap. For an FEL near the CBM, the charge state ${Mn}_{Ga}^{-}$ is more stable, whereas the charge state ${Mn}_{Ga}^{+}$ is more stable for an FEL near the VBM [275]. The formation energies are relatively high, around 2 eV at N-rich conditions [275].

**Mn, Fe, Zn group:** This group represents all transition metal impurities in GaN crystals that can be reduced in concentration by using a liner. Typically, electron-rich transition metals form deep level carrier traps, so they are of the greatest concern [86]. The solubility limit of Mn in GaN crystals is <1%, 0.03% for Fe, and > 1% for Zn [205], in the basic growth atmosphere. The incorporation risk of these impurities in GaN decreases with a decreasing solubility and an increasing formation energy. Accordingly, the contamination risk by Fe should be lower than by Zn (at least for an FEL near the CBM). The contamination of Mn, Fe, and Zn has its origin from the inner autoclave wall. A significant improvement should be reached via the usage of a hermetically sealed liner instead of a pressure-balanced liner. The effect of the liner will be discussed in more detail in the Section 6.1.

**Carbon:** C goes predominately to the N site [246]. Experiments showed that the Ga site becomes only relevant at high C contamination concentrations [246]. The neutral complex C_N_–H_i_ has a lower formation energy for an FEL near the VBM than C_N_ [266] at N-rich and Ga-rich conditions. This C_N_–H_i_ complex will likely form in the ammonothermal system at an FEL near the VBM, where the formation energy is lower than the formation energy of C_N_. At the N site, C acts as a deep acceptor at 1 eV above the VBM [246]. The formation energy of the C_N_ state decreases for an FEL near the CBM. For an FEL, near the VBM, the neutral charge state, and nearest to the VBM, a donor charge state gets more stable at 0.4 eV above the VBM [246]. A further cleaning alongside a refined pre-growth etching process is expected to lead to the complete elimination of the C impurity [87].

**Sodium:** Na goes predominantly on the interstitial site as a donor, with a lower likelihood on the Ga site as an acceptor [264]. Both sites are stable, so they are self-compensating. Other mineralizers like Li and Ka can potentially show the same behavior, however, the formation energy of K at the Ga site is dramatically high, causing it to act predominantly as a donor [264]. The incorporation of sodium in GaN is non-uniform from run-to-run [87], and it differs according to the growth facet [105]. Other mineralizers in the basic (alkali metals) and acidic (halogenides) environment have been reported to behave similarly [87]. So far, a mineralizer is needed for the ammonothermal growth of GaN and cannot be omitted. However, the growth conditions can be changed to increase the formation energy of sodium on the interstitial-site in GaN. Another way is the usage of other mineralizers that are not as easily incorporated, for example potassium in GaN.

**Hydrogen:** H incorporates in GaN as an interstitial or forms complexes (for more details, see Section 5.1).

**Oxygen:** Independently of the FEL, O is stable on the N site at the $O_{N}^{+}$-state [65] and forms a shallow donor at 4–29 meV below the CBM [276].For an FEL near the CBM, the complexes V_Ga_–O_N_–2H, V_Ga_–O_N_–H, and V_Ga_–O_N_ are predicted to form more stable states as O_N_ at Ga-rich conditions [250,266]. The formation energy of O_N_ varies by only about 0.5 eV between Ga-rich and N-rich conditions [266]. Accordingly, the formation energy relationship between the complexes and O_N_ should not change much. The mentioned relationship should also be valid for N-rich conditions. Due to the high amount of H and V_Ga_ in the ammonothermal system, it is likely that V_Ga_–O_N_–2H, V_Ga_–O_N_–H, and V_Ga_–O_N_ will be formed. Experiments showed that the mentioned complexes are formed, but are not dominant. Instead, the V_Ga_–H_x_ complexes are predominant in ammonothermal GaN (see Section 5.1). The complexes V_Ga_–O_N_–2H, V_Ga_–O_N_–H form deep acceptor states [250]. V_Ga_–O_N_–2H has the lowest formation energy and an activation energy of ca. 1 eV above the VBM [277]. As mentioned in Table 5, there are three known oxygen sources.

Reduction of oxygen impurities originating from adsorbates on surfaces is likely to be achieved via improved cleaning, dehydration, and pre-growth etching process refinements [87].

For the basic ammonothermal system, a reduction in oxygen contamination from the mineralizer can be achieved via selection of precursors with lower oxygen and moisture sensitivity. For example, in sodium, it decreases in the following order: sodium amide, sodium azide, and metallic sodium [263]. The experiments showed a clear tendency that the growth rate and crystal quality increases with a decreasing sensitivity to oxygen and moisture of the Na mineralizers, which implies a lower oxygen/contamination content of the GaN crystal [263]. This observed behavior for a Na mineralizer should be transferable to other basic mineralizers.

For the acidic ammonothermal system, the purity can be improved via two methods. The first one is to use NH_4_F as the mineralizer and perform crystal growth in the observed retrograde solubility regime, instead of NH_4_Cl, NH_4_Br, and NH_4_I in the normal solubility regime. That has been observed to lead to a reduction of oxygen from 1–1.5 × 10^20^ cm^−3^ to 8 × 10^18^ cm^−3^ [103]. This improvement can be attributed to the resulting higher growth temperature and/or the different chemical characteristics of NH_4_F (the smallest radius/charge ratio) [103]. The second method is the sequential introduction of purified NH_3_ and HCl gasses instead of highly hygroscopic NH_4_Cl powder, which enables the reduction of the oxygen concentration from 1–1.5 × 10^20^ cm^−3^ till 3–5 × 10^19^ cm^−3^ [262]. According to Tomida et al. [262], that technique should also be transferable to other halides.

All these mineralizer purification steps lead to a reduction of the concentrations of the elements from the Si, Al, Mg group as well.

The last contamination source is the GaN nutrient, which is usually polycrystalline GaN. To improve the purity of the polycrystalline GaN source material, S. Suihkonen et al. [87] point out that the purification processes of starting materials need to be developed further. No publications were found that further pursued this path.

**Impurity getters**: If introduction of oxygen into the autoclave is unavoidable, the use of getters is a remaining means of reducing the oxygen concentrations in the crystals. Getters have the primary focus of chemically interacting with the impurity element in the fluid phase and binding them in an insoluble compound, thereby effectively removing them from the growth environment.

Some earlier reports on impurity getters were published in 1997 by R. Dwiliński et al. [278] and in 2007 by B. T. Adekore et al. [170]. R. Dwiliński et al. [278] used erbium (Er) as the getter material with a molar Er to GaN ratio of 1:10. Grown GaN crystals exhibited a decreased intensity of the yellow band and a sharper PL-emission peak for GaN in the PL spectrum. That indicates a lower concentration of free electrons [278], likely due to reduced presence of oxygen as a donor. B. T. Adekore et al. [170] also used Er as an oxygen getter with an Er to GaN weight ratio of 1:3–4. With these conditions, Er became unintentionally doped into the bulk GaN single crystal [170]. They mentioned oxygen was gettered, as they measured an O concentration of 1 × 10^19^ cm^3^ and 7 × 10^19^ cm^3^ at the Ga-polar and N-polar sites, respectively (measured via SIMS). However, Er was unintentionally incorporated into the bulk GaN single crystals [170], with a concentration of 10^18^ cm^3^ to 10^17^ cm^3^. In 2018, D. Tomida et al. [158] found that Al works well as an oxygen getter material in the acidic environment. They explained their results with the low standard Gibbs’ formation energies of Al_2_O_3_ at 600 °C.

**Facet-dependency**: Incorporation mechanisms of impurities depend on the atomical scale morphology and the crystallographic orientation. That the incorporation of impurities depends on the growth crystallographic orientation is well known and is found in other growth techniques, like the Czochralski method by Si [279]. In the ammonothermal growth method, six growth faces are typically observed, though only four of these are stable [100,105,145,280]:
(0001)/Ga-face/+c-plane; (000-1)/N-face/−c-plane;{10-10}/m-plane; {11-20}/a-plane;{10-1-1};{11-22}.

The stable growth faces in the basic system are the +c-plane, −c-plane, and m-plane [100]. In the acidic system the stable growth faces are the same, but the {10-1-1}-plane may terminate the habitus of the crystal instead of the −c-plane [100].

S. Sintonen et al. [145] observed in basic ammonothermal GaN a higher oxygen incorporation in wing regions (which is mainly formed by a-plane growth) and a lower incorporation in the c-direction, measured via SIMS. For MOCVD, the same behavior was explained with the nitrogen surface saturation of a-plane, which defines the oxygen incorporation efficiency [145]. Likewise, for the ammonobasic environment, S. Pimputkar et al. [140] observed the highest oxygen incorporation in the a-plane, measured via SIMS. The oxygen concentration they measured decreased in the following order, depending on the growth direction: a-plane, −c-plane, +c-plane. M. Zając et al. [105] measured a lower oxygen concentration at the m-plane and a higher oxygen concentration at the −c-plane, via SIMS. However, S. Sintonen et al. [145] mentioned that the wing regions (such as the m-plane) show an inhomogeneous incorporation of impurities. The differences between the values for the +c-plane and the m-plane are, however, not high enough to explain the differences by these inhomogeneities. L. Wenhao et al. [280] measured, via SIMS, a higher oxygen incorporation in the {11-22}-plane and a lower oxygen concentration in the a-plane in ammonobasic GaN crystals.

Accordingly, for the ammonobasic crystal growth, the following oxygen impurity incorporation tendency can be given: {11-22}-plane > a-plane >> −c-plane and m-plane > +c-plane.

Considering that the growth rates in the acidic ammonothermal system are higher on the m-plane and +c-plane in relation to the basic ammonothermal system [103,105], the facet-dependent dopant incorporation behavior will likely be different for the two environments. K. Shima et al. [242] measured the oxygen concentration in ammonoacidic GaN, via SIMS, for GaN grown on different crystallographic planes; their measurements show that in the −c-plane, oxygen incorporation was enhanced by a factor of 10 or more, in relation to the +c-plane. For the ammonoacidic crystal growth, the following oxygen impurity incorporation tendency can therefore be stated: −c-plane >> +c-plane.

**Growth time-dependency:** S. Sintonen et al. [145] measured no significant time-dependent impurity changes of an ammonobasic grown GaN crystal. 

**Figure 13:**
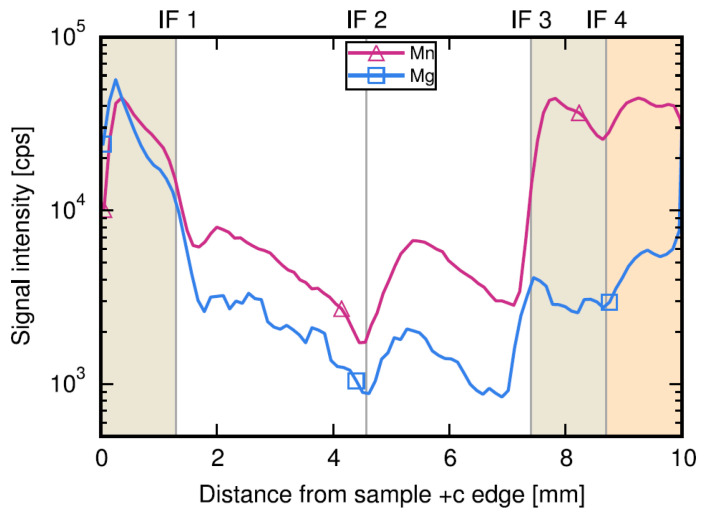
LA-ICP-MS spectrogram for Mn and Mg as function of the distance from the seed in growth direction (+c-edge of the sample). IF1-IF4 indicating the start of a new lamellae, the colored regions in the graph give the visual impression of the crystal. Reprinted from S. Sintonen et al. [146], copyright (2016), with permission from Elsevier.

A different report by S. Sintonen et al. 2016 [146] on a GaN crystal grown from a different group found that the impurity incorporation increases at the beginning of the growth and slowly decreases after reaching a maximum. This behavior was clearly observable by laser ablation inductively coupled plasma mass spectrometry (LA-ICP-MS) measurements at Mn and Mg (Figure 13). In their experiments, S. Sintonen et al. [146] grew one crystal in five growth steps and in three different autoclaves, which results in five layers/lamellae in one crystal. They explained this behavior with the effect of a decreasing Ga concentration in the supercritical NH_3_ via the reduction of the feedstock amount during the growth. This reduced Ga concentration in the solution leads to a lowering of the viscosity of the solution and hence, to a reduced convective transport of impurity species from the autoclave wall to the sample.

The results of B. Wang et al. [281] showed crystal growth time-dependent impurity changes in the ammonobasic system, as did S. Sintonen et al. [146]. B. Wang et al. [281] measured a decay of the yellow and blue luminescence band (measured via cathodoluminescence) along the growth direction of this crystal. The measurements indicated a time-dependent decay of the impurity concentration and/or the defect concentration along the growth direction [281].

It could be that the GaN feedstock of S. Sintonen et al. [145] was so large that they could not measure significant changes. The enlargement of the growth period with a lower incorporation rate could be suitable measure for a further reduction of the impurity incorporation rate in ammonothermal GaN crystals.

### 5.3. Effect of Point Defects on Electrical, Oprical and Structural Properties

Intentional and unintentional doping significantly influence crystal properties. The better the intentional dopability of a crystal, the higher the range of crystal properties that can be reached (in a controlled way).

In this section, we discuss the effect of all point defects and their effects on the electrical, optical, and structural properties. The formation of unintentional point defects, both native defects and impurities, is discussed in Section 5.1 and Section 5.2.

#### 5.3.1. Electrical Properties

In this first section, we concentrate on dopability, because doping influences not only electrical properties, but also the other crystal properties, which will be discussed in the subsequent sections.

Currently, is it possible to crystallize n- and p-type conductive and semi-insulating ammonothermal GaN. These conductivity types are dependent on further development of the reduction of various impurities and native point defects, above all, oxygen. Oxygen as an impurity enormously hinders the use of other dopants in GaN and hinders the oxygen dopability in a low carrier concentration/high carrier mobility range. Currently, it is not possible to dope ammonothermal GaN without oxygen as a counter-dopant (compensating donor) or co-dopant. Accordingly, oxygen is the mostly used dopant in GaN [86]. To achieve lower doping levels, better cleaning procedures (Section 5.2) or oxygen getters are seen as prospective measures to reduce oxygen concentrations, which cannot currently be removed in advance. H and Fe (for example) can be additionally present in ammonothermal GaN, with these elements leading to a degradation of electrical devices. H can be mobile at elevated temperatures or high electron fluxes during device operation, leading to unintended or uncontrolled passivation of p-type GaN layers [87]. Fe can form effective SRH centers, leading to device degradation for optoelectronic emitters or current collapse in AlGaN/GaN high electron mobility transistors (HEMTs) [87].

Table 6 shows the currently used dopant elements yielding conductive or SI ammonothermal GaN, along with their growth environment and electrical properties. We discuss the dopability in the sense of doping efficiency: Which fraction of the incorporated atoms acts as a dopant. The doping efficiency values in Table 6 were calculated by dividing the charge carrier concentration by the dopant concentration. The doping efficiency can be max. 100%, like for oxygen doping in the HVPE method [252]. Only in the presence of co-dopants, the value can be higher than 100%.

In an ammonothermally grown n-type oxygen-doped GaN crystal, the doping efficiency is 10% to 88%, compared to 100% by GaN crystals from the HVPE process. The doping efficiency of p-type magnesium-doped GaN is two orders of magnitude lower than for oxygen-doped ammonothermal GaN.

In the following, we discuss Table 6 according to the conductivity types. 

**p-type:** The low doping efficiency for p-type Mg-doped GaN can be explained by three contributions. The first one is unintentional compensating impurities. In the ammonothermal method, specifically, high oxygen concentrations, are currently unavoidable. The oxygen acts as a compensating donor that reduces the concentration of free holes. For example, the oxygen concentration of the Mg-doped GaN listed in Table 6 is 1.5 × 10^18^ cm^−3^ [105]. The second contribution is the relatively large activation energy of the acceptor Mg_Ga_ at 200 meV above the VBM [105]. The third contribution is the high likelihood of Mg_Ga_ to form the neutral Mg_Ga_–H complex (see Section 5.2) [105]. The third contribution can be addressed by the removal of H from the Mg–H complex via annealing (Table 6), thereby increasing the charge carrier concentration and decreasing the resistivity from 10^6^ to 30 Ωcm [105].

**n-type:** The doping efficiency < 1 of oxygen in ammonothermal GaN crystals can be explained by the formation of V_Ga_–H complexes, which can act as acceptors (Section 5.2). W. Jiang et al. [252] compared their Hall mobility measurements of oxygen-doped ammonoacidic GaN with HVPE GaN in the same carrier concentration range. The charge carrier mobility values were similar in their charge carrier concentration ranges of 1.5–27 × 10^18^ cm^−3^ and 0.66–15 × 10^18^ cm^−3^, respectively. Y. Mikawa et al. [284] observed, with their Hall mobility measurements of oxygen-doped ammonoacidic GaN, the same similarity of the carrier mobility from ammonoacidic GaN and HVPE GaN.

The doping efficiency of the oxygen-doped basic ammonothermal GaN boules reported by S. Pimputkar et al. [282] is the lowest listed in Table 6. Their crystals also had the highest doping level and lowest charge carrier mobility, as can be seen in Table 6. At such high oxygen concentrations, with an FEL close to the CBM, the previously mentioned behavior does not seem to be valid. Given the high concentration of oxygen, it is possible that a large number of hydrogenated Ga–vacancies formed and acted as acceptors, due to an FEL closer to the CBM. This, in turn, would lead to reduced doping efficiency and a high concentration of additional point defects, further reducing mobility.

Accordingly, via the growth of oxygen-doped basic ammonothermal GaN, two doping efficiency ranges exist. Below an oxygen doping level of 27 × 10^18^ cm^−3^, there are no predominant acceptors and the doping efficiency is high. Above an oxygen doping level of 50 × 10^18^ cm^−3^, predominant acceptors exist, and the doping efficiency is low.

**SI:** Mg and Mn are used as p-type dopants to compensate the process-related unavoidable oxygen n-type doping. The oxygen concentration of the SI Mg-doped GaN listed in Table 6 is 1–2 × 10^18^ cm^−3^, whereas the oxygen concentration of the SI Mn-doped GaN is 5 × 10^18^ [105].

As can be seen from Table 6, for Mg, the concentrations of Mg-doped SI GaN are lower than those of Mg-doped conductive p-type GaN, to realize a lower resistivity. Contrary to the conductive Mg-doped GaN, the annealing (N_2_ atmosphere/1100 °C/4 h) of the SI Mg-doped GaN leads not to a resistivity reduction [105]. Conductivity of the SI Mg-doped GaN could only be measured above 400 °C. At this temperature, the GaN is n-type conductive with a carrier concentration of 10^12^–10^13^ cm^−3^, due to an activation energy of 1.46 eV above the VBM [105]. FTIR analysis shows no evidence of Mg–H bonds before and after the annealing [105]. Accordingly, no Mg–H bonds were broken, which would result in a lower resistivity.

SI Mn-doped GaN shows the highest resistivity, as shown in Table 6. This results from two circumstances: firstly, the charge carrier compensation effect and secondly, the characteristic of transition metals to form deep levels that trap charge carriers. A high-temperature-annealing (N_2_ atmosphere/1100 °C/4 h) does not yield a resistivity reduction, like for SI Mg-doped GaN [105]. The energy level of Mn in this SI Mn-doped GaN was determined to be 1.5 eV above the VBM [105].

#### 5.3.2. Optical Properties

A first assessment of the presence of optically active point defects can be made via a visual inspection by the naked eye. The ammonoacidic and ammonobasic systems have different amounts of specific point defects, which influence the coloration [100]. Accordingly, crystals have a growth environment dependent coloring:

- Ammonobasic: grey, yellow, green, or orange-brown to red [100,105,280];
- Ammonoacidic: grey, yellow, or green [100,285]

Independently of the growth environment, crystal greying depends on n-type doping [100]. As discussed in Section 5.3.1., the n-type doping is currently only represented by oxygen doping. Accordingly, the greying of the crystal gives a first indication of the oxygen doping level of the crystal. In the ammonobasic and -acidic system, the yellow coloring is connected to the presence of deep level defects. These defects are primarily Ga–vacancies and their related complexes, according to their predominant amount (Section 5.1) [105,242]. The green coloring of ammonoacidic GaN and p-type Mg-doped GaN is predicted to be linked to N-vacancies [244,256]. The color of SI Mn-doped GaN crystals ranges from orange-brown to red [105,280].

**Figure 14:**
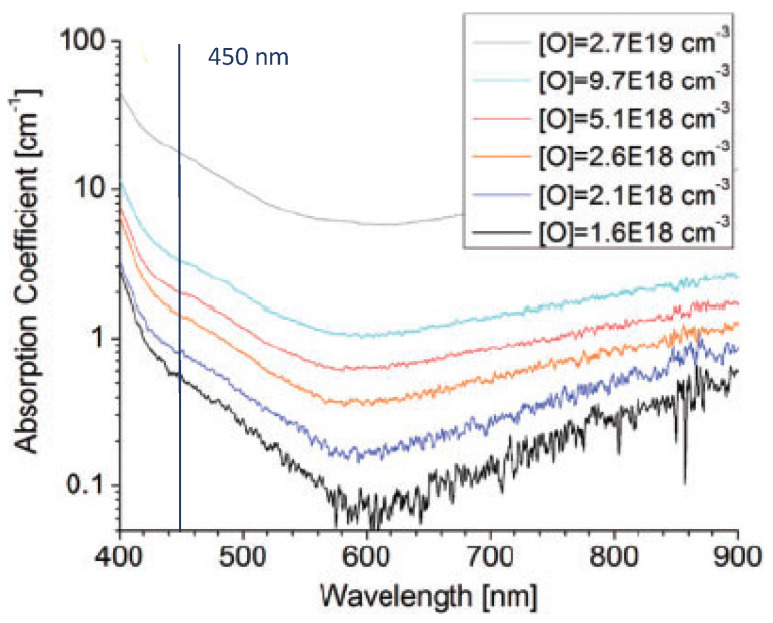
Dependency of the optical absorption coefficient (*α*) on the wavelength for six oxygen-doped ammonoacidic GaN crystals, with a marking of blue LEDs relevant 450 nm wavelength (in blue). Reprinted and modified from [252] with permission, © 2017 The Japan Society of Applied Physics.

The visually observable greying of the crystals can be described via the increase of free charge carriers. In the ammonobasic and ammonoacidic systems, an increase of the absorption coefficient can be observed with increasing amounts of free electron/oxygen concentration, likely due to phonon-assisted free carrier absorption [252,282,286]. Figure 14 shows the absorption spectra for six crystals grown in an ammonoacidic environment, with an oxygen concentration from 1.6 × 10^18^ to 2.7 × 10^19^ [252]. According to W. Jiang et al. [252], the optical absorption at wavelengths > 600 nm (<2 eV) is similar to that of HVPE GaN. In the region between 500 nm (2.5 eV) and 400 nm (3.1 eV), closer to the absorption band edge, the absorption coefficient of ammonothermal GaN increases in comparison to the HVPE GaN. S. Pimputkar et al. [282] observed a comparable behavior in GaN grown in an ammonobasic environment. They observed this sharp increase of the absorption coefficient for ammonothermal GaN at 2.9 eV (427 nm) and for HVPE GaN at 3.2 eV (387 nm). They measured their ammonothermal and HVPE samples via FTIR and could detect hydrogenated Ga–vacancies in the ammonothermal GaN, but not in HVPE GaN. Additionally, they measured the refractive indexes at the same samples, the indexes suggest defect states in the ammonothermal GaN close to the VBM, but not in HVPE GaN. Accordingly, it can be suggested that hydrogenated Ga–vacancies are the primary cause of the increase of the absorption in the region of 2.8–3.3 eV (442–375 nm). In the FTIR spectra of ammonoacidic GaN reported by W. Jiang et al. [252], absorption peaks that correspond to V_Ga_–H complexes were likewise detected. In ammonobasic GaN, E. Letts et al. [286] also verified an oxygen concentration-dependent increase of the absorption coefficient. In conclusion, the absorption behavior of the ammonoacidic and ammonobasic GaN seems comparable.

According to Section 5.3.1, the oxygen doping efficiency in ammonothermal GaN ranges from 10% to 90%. Due to that, it can be assumed that the reduction of the phonon-assisted free carrier absorption ranges in ammonothermal GaN from 90% to 10% in relation to the case if every incorporated atom would act as a dopant.

The yellow luminescence band (YL) measured via PL can be observed in ammonothermal GaN, as well as in HVPE GaN, and is linked to deep level defects [273,287], like the visually observable yellowing of GaN crystals. M. Reshchikov [273] detected an ammonothermal GaN-specific yellow luminescence band with a maximum at 2.2–2.3 eV [244,273], which he attributed to the V_Ga_H_3_ complex. In addition to the YL, the green luminescence band (GL) at 2.33–2.4 eV (maximum), red luminescence band (RL) at 1.72–1.82 eV (maximum), and blue luminescence band (BL) 2.7–3.0 eV (maximum) can be observed in ammonothermal GaN [244,273]. The GL is mainly linked to N–vacancies, the RL is mainly linked to N–vacancy complexes, and the BL is mainly linked to impurities [244,273].

W. Jiang et al. [288] reported the growth of a highly transparent yellow-tinted GaN crystal in an ammonoacidic environment. That crystal had an absorption coefficient of 0.75 cm^−1^ at the emission wavelength of blue LEDs (450 nm), with a carrier concentration around 1–3 × 10^18^ cm^−3^. This absorption coefficient is one of the lowest published absorption coefficients in that oxygen concentration level. Point defects decrease the active region efficiency of LASER diodes (LDs) [216,289] and are linked to increase the threshold current of LDs [290], so they are of great concern and thus have to be reduced.

Further, L. Wenhao [280] showed the possibility to shift the longitudinal optical mode E1(LO) of ammonobasic GaN towards a lower frequency via Mn doping. That opens the possibility to use Mn-doped GaN for infrared emission devices.

#### 5.3.3. Structural Properties

To avoid stress generation during the crystal growth of bulk GaN [110], or during the epitaxial deposition of crystalline layers [291], the lattice parameters have to be as similar as possible. Too high a lattice mismatch, in combination with layer thicknesses over 30–40 µm, lead to large bending and an increase of dislocation density [292]. Even the use of HVPE seeds for the ammonothermal growth of GaN, which results in a relatively small lattice mismatch, leads to multiple negative effects [110,293], including (1) a reduction of the radius of curvature (as a measure of the bending/plastic deformation), (2) an increase of the rocking curve width (as a measure for the mosaicity), (3) an increase of the etch pit density (as a measure of the amount of threading dislocations), and (4) the generation of cracks. E. Letts et al. [293] observed cracking of their crystals grown on HVPE seeds only above a crystal layer thickness of 1 mm. They explained this behavior by a flipping of the sign of the radius of curvature at a critical length where the radius of curvature becomes infinite. They assumed that the differences of the radius of curvature originate from the differences of the lattice spacing due to the different level of contamination of the two growth methods.

Four main factors influence the lattice parameters of GaN: free electron concentration, dopant concentration, native defects, and lattice mismatch to the substrate [291]. The first three points are linked to each other and can be summarized as two effects of point defects on the GaN lattice [294]. Firstly, the lattice deformation, due to a size effect, related to the difference in size of the impurity atom and the substituted lattice atom. Secondly, the lattice deformation due to the electronic effect related to a change of the conduction or valence bands by the excitation of free carriers into the bands and the associated volume change.

Free carriers (holes and electrons) expand the GaN lattice [292,294]. According to C. Van de Walle’s [294] calculations, electrons expand the lattice three times more than holes. The size effect can expand or contract the lattice, depending on the size of the impurity atom in relation to the substituted lattice atom [294]. A general weighting relationship between the size effect and the electrical effect does not exist. For example, the size effect-related lattice deformation is 33% lower for oxygen at the N-site and 76% higher for Si at the Ga site compared to the electrical effect for electrons in GaN. The size effect can mainly be treated as isotropic, and the electrical effect is likewise assumed to be isotropic [294]. The element in which charge state occupies which lattice site is described in Section 5.2 and Section 5.3.

Calculations of the lattice parameter of GaN show a linear dependency on the dopant concentrations [291,292,294]. M. Leszynski et al. [291] showed experimentally the increase of the lattice parameters *a* and *c* with the free electron concentration of GaN for homo- and heteroepitaxial, as well as bulk crystal growth.

A difference of the lattice parameters of 0.02% can already lead to technical implications [292]. Depending on the theory for the calculation of the lattice parameters, the values differ, for example by 3% [270]. At the moment, it is not possible to grow GaN without any lattice deformation. For example, the lattice-deforming oxygen impurity is currently unavoidable in ammonothermal GaN. Accordingly, the determination of precise undistorted lattice parameters of GaN is currently not possible. According to C. Van de Walle [294], an oxygen concentration of 10^20^ cm^−3^ leads to the lattice parameters *a* = 3.19798 Å and *c* = 5.18293 Å, assuming every O atom occupies a N site and leads to a free carrier, and the lattice parameters for pure GaN are *a* = 3.19 Å and *c* = 5.17 Å [270] (modeled lattice parameters). For the lattice parameter *a* and *c*, that means an increase of 0.25%. For an ammonothermal GaN crystal, R. Kucharski et al. [110] measured two values for the lattice parameter *a*: 3.1881(5) Å and 3.1887(5) Å, and one value for the lattice parameter *c*: 5.1854(5) Å. The crystal was grown on an HVPE seed (radius of curvature of the seed ~100 m) with an oxygen concentration of 5 × 10^17^ cm^−3^. Due to the uncertainty of undisturbed lattice parameter values of GaN, no quantitative interpretation of any measured lattice parameter of ammonothermal GaN is possible, regarding the influence of a point defect on the lattice parameter. However, the statement can be made that the oxygen concentration in ammonothermal GaN crystals plays a huge role in the lattice parameter.

Besides their influence on the lattice parameters, it should be noted that point defects are needed for the dislocation climbing process and thus, they influence the propagation of dislocations, as reported for V-shaped dislocations in epitaxial GaN layers grown by MOVPE [295].

In summary, a wide range of point defects and associated complexes have been identified to exist in GaN and likely, in similarly grown ammonothermal crystals. Control over these defects is critical to maintaining desired crystal properties, though the persevering oxygen contamination of the system can make the pursuit of certain material properties (such as p-type doped semiconductors) a challenge that requires further targeted research efforts. Nonetheless, significant demonstrations have been made, yielding controlled changes to electron conductivities in materials along with changes to optical transparency, both of which are critical to control for substrates, especially for optoelectronic devices.

Significant efforts were made to explore these material properties and how they relate to the growth environment and growth processes. These experiments are generally considered tedious and time consuming, as numerous in situ technologies developed for lower pressure system cannot readily be applied to these high-pressure systems. Combining knowledge of the reactivity and solubility of species with the evolving needs for demonstrating high quality crystal growth has led to continuous development of the growth systems themselves. The following section discusses the various aspects associated with development of reactor technologies suitable for ammonothermal environments, including systems with improved materials leading to higher purity, and/or higher temperature systems, or via integration of windows or feedthroughs to permit the development of in situ technologies.

## 6. Advances in Reactor Technology

The reactor in the ammonothermal process has to primarily meet two mechanical requirements: high strength and creep resistance permitting high pressure retention (up to ~500 MPa [96]) at elevated temperatures (until~600 °C [84], or beyond), and high corrosion resistance under basic or acidic ammonothermal growth environments.

For the load-bearing components that are exposed to high temperatures, nickel–base superalloys such as Inconel 718 or Rene41 are commonly used, though a molybdenum–base alloy has also been employed [164].

**Figure 15:**
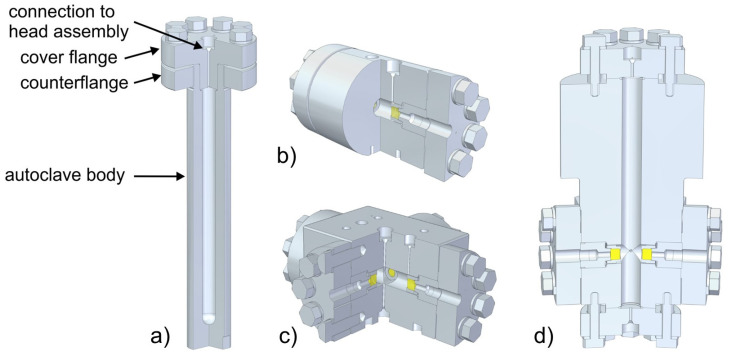
Visualization of the geometries of different types of autoclaves: (**a**) autoclave as used for crystal growth using a temperature gradient, as well as for exploratory synthesis or studies on intermediates using ex situ characterization methods, (**b**) uniaxial optical cell, (**c**) biaxial optical cell, (**d**) optical cell designed for in situ diffraction. Reproduced from [299], with minor adjustments to collect only the autoclave types relevant to this review in one figure.

Initially, ammonothermal reactors were developed for crystal growth or the synthesis of new materials, resulting in a long tubular geometry that is both comparatively simple to manufacture and permits the use of relatively thin autoclave walls. The moderate wall thickness prevents excessive heat conduction via the autoclave walls from one temperature zone to another. Thereby, it facilitates the implementation of thermal gradients inside the autoclave, which is an important requirement of crystal growth exploiting the temperature dependency of solubility [296]. An example of such a tubular autoclave geometry can be seen in Figure 15a. The inner and outer diameters of this autoclave are 21 mm and 50 mm, respectively.

More recently, specialized types have been developed for the purpose of implementing different in situ monitoring techniques, starting with an optical cell for video-optical and UV-Vis measurements developed by N. Alt and E. Schlücker [297,298]. Optical cells are defined as autoclaves that are designed to incorporate viewing windows. To achieve this, a material transparent to the radiation in question (such as optical or UV-light, or X-rays) is utilized. In addition to sufficient transparency, the window material needs to possess sufficient mechanical strength and chemical stability in the targeted ammonothermal environment [299]. For 2D imaging using optical light or X-rays, as well as for spectroscopic measurements, the same design of optical cells can be used. These optical cells can flexibly be equipped with the aforementioned window materials [300].

Examples of state-of-the-art optical cells are shown in Figure 15b–d. A defining characteristic of uniaxial optical cells (Figure 15b) is the single, straight beam path with windows at both ends. A biaxial optical cell Figure 15c, on the other hand, comprises a cuboid-shaped viewing cell with two beam paths running perpendicular to each other. Moreover, a specialized autoclave with windows set at the diffraction angle was developed specifically for in situ X-ray diffraction studies (Figure 15d). Unlike the other optical cells, the optical cell in Figure 15d is designed to allow for crystal growth via a thermal gradient and fluid convection.

The simplest (uniaxial) optical cells of the construction depicted in Figure 15b–d have an outer diameter of 108 mm. The thicker walls of the autoclave can impact the thermal field within the system significantly. Firstly, the greater thermal inertia of the thicker walls can lead to slower responses to changes in heating or cooling conditions, affecting the overall thermal responses of the system. Secondly, the enhanced heat conduction along the autoclave walls causes a very uniform temperature distribution inside the autoclave [299]. Such a uniform temperature distribution is intended in the case of studying solubility or dissolution kinetics for a distinct temperature condition, however, measures to mitigate this effect need to be taken if combining optical cells with crystal growth experiments via a thermal gradient [299]. Such a design, with a thinned autoclave wall in-between nutrient and crystal growth zone, is depicted in Figure 15d.

Another important design aspect is the orientation of the tubular inner volume in relation to gravity and, if a thermal gradient is imposed, in relation to the thermal field. The horizontal orientation of the reaction chamber in uniaxial and biaxial optical cells serves multiple functions. Firstly, it enables the convenient alignment of optical components along a single axis or multiple axes, depending on the configuration of the cell. This alignment is critical for accurately measuring light interactions with samples and ensuring precise spectroscopic analysis. Importantly, the horizontal orientation with regard to gravity suppresses fluid convection (optical cells in Figure 15b,c), whereas a vertical orientation with respect to gravity can be applied to enhance fluid convection (optical cell designed for crystal growth, Figure 15d [299].

Current topics in ammonothermal reactor technology are driven by the following factors: general design considerations and the availability of alloys with high mechanical strength at high temperatures, improving both reactor lifetime and crystal purity by mitigating corrosion issues, and advancing the fundamental understanding by gaining experimental access to the physical and chemical processes during the growth. The following sections are dedicated to these aspects.

### 6.1. General Design Considerations

The most important challenges for the load-bearing parts of ammonothermal autoclaves will be delineated in the following. We aim to shine a light on specific aspects that are and have been important to past and future development of the field, rather than providing comprehensive information. Readers who require more comprehensive information are referred to books covering the design of high-pressure vessels in general, and for ammonothermal experimentation in particular [301,302].

**Figure 16:**
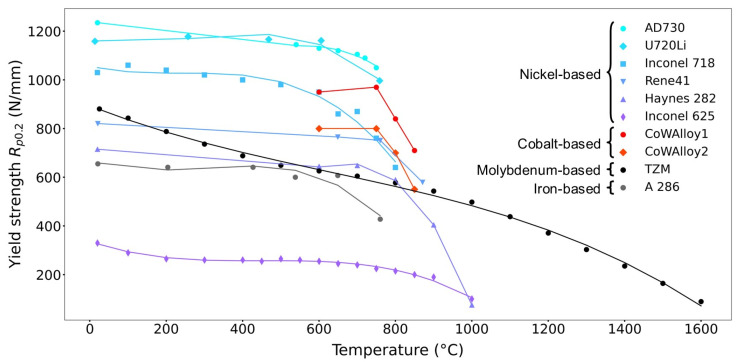
Decline of yield strength with increasing temperatures for several alloys used or considered for use as load-bearing material for hydrothermal and ammonothermal reactors, respectively. Data taken from the same references as in [304], while the data for stainless steel (A 286) were taken from [307]. The fit lines model the yield strength as a third degree polynomial and serve as a guide to the eye.

Given the need to simultaneously withstand high temperature and high mechanical stress due to the inner pressure over extended periods of time, the alloy is the main limiting factor preventing experiments at simultaneously higher temperatures and higher pressures [166]. An important aspect is that yield strength $R_{p0.2}$ generally decreases with increasing temperature, as is evident from the data shown in Figure 16. Nickel–base alloys are the state-of-the-art materials, primarily due to their excellent mechanical strength at elevated temperature, achieved via precipitation hardening [166]. An added layer of complexity originates from the limited chemical stability of most prospective load-bearing materials in ammonothermal solutions, which depends strongly on the mineralizer and conditions used [147,166]. While nickel–base alloys generally exhibit good corrosion resistance, as well as oxidation resistance at high temperature, this is not necessarily the case for ammonothermal conditions, and stress corrosion cracking is a major concern in contact with ammonoacidic solutions [165,166,303]. More recently, cobalt-based alloys have been considered [166,304], as the system Co-Al-W provides the opportunity to harden the alloys by the precipitation of the ordered phase (γ′) in the Co solid solution matrix phase (γ), and the higher melting point of Co results in high liquidus and solidus temperatures [166]. Aiming for higher temperatures at the expense of maximum operating pressure, the molybdenum-based alloy titanium–zirconium–molybdenum (TZM) [164] and the nickel-base alloy Haynes 282 [177,186,301,305,306] have been used for ammonothermal autoclaves. For comparison, an iron-based alloy with high mechanical strength at elevated temperatures is also shown in Figure 16. The high temperature strength of iron-based alloys (stainless steels) is insufficient for most ammonothermal applications, as their mechanical properties are insufficient at temperatures above 500 °C [301]. In this context, the significantly different temperature range of 345–360 °C for hydrothermal growth of quartz in comparison to 400–800 °C ammonothermal growth of GaN (see Table 1) should be noted.

Another important requirement is sufficient creep resistance [166,301], which is due to the combination of high temperatures (in relation to the melting points of the alloys) and the long exposure to these elevated temperatures (often several weeks or even months per experiment). To predict the creep life of components, parametric models such as the Larson–Miller relation [308] are commonly used [166,309]. In the high temperature range (at temperatures above half the melting temperature), the creep rate is proportional to the diffusion coefficient of atoms and consequently to temperature, as well as to the applied stress [310,311]. Emerging superalloys appear to be promising also in terms of better creep resistance: Specifically, the cobalt–base alloys CoWAlloy1 and CoWAlloy2 were reported to have significantly higher creep resistance than the nickel-based alloys 718 and 282 [166].

Ductility (often evaluated by fracture toughness) is also an important consideration, especially from a safety point of view. This is a particular concern in the case of the molybdenum-based alloy TZM, for which the brittle-to-ductile transition occurs above room temperature, specifically at 100 to 150 °C [164] or even 400 °C depending on the strain rate [312].

The permittable internal pressure *p* depends on the yield strength $R_{p0.2}$, the inner diameter *d_in_*, the outer diameter *d_out_* as follows [302]:

(1) $$p=R_{p0.2}\cdot \frac{1}{\sqrt{3}}\cdot ln\left(\frac{d_{out}}{d_{in}}\right)$$

To increase the size and number of crystals grown in one run, the inner diameter of the autoclaves needs to be increased. The difference between the stress at the inside and the outside increases with the square of the diameter ratio, with the stress being substantially higher at the inside [302]. Consequently, if the ratio of outer and inner diameter decreases, the permittable internal pressure decreases. A proportional increase of both, however, increases both the outer diameter and the wall thickness. Depending on the alloy, the former can be a challenge in terms of availability and the latter can likely be a challenge in terms of maintaining a suitable thermal gradient between the two zones. With the hydrothermal growth of quartz, a similar method for the simultaneous growth of large numbers of single crystals has been long demonstrated to have impressive scalability [122]. To enable the use of a steel in place of the commonly employed nickel–base superalloys as the pressure-bearing material, M. D’Evelyn and coworkers from Soraa Inc. (now KYOCERA SLD Laser, Inc., Goleta, CA, USA) developed a new design for an ammonothermal reactor using internal heating [90]. By placing the heater inside the pressure vessel (protected against corrosive attack by a hermetically sealed capsule) and by using a ceramic shell as thermal insulation between the heater and the pressure-bearing reactor walls, they drastically reduced the temperature at which the pressure-bearing parts are held [90,313]. For a heater temperature of 750 °C, the temperature drops to 200 °C within the ceramic shell [90]. This allows this autoclave design to achieve pressures as high as 600 MPa, which has demonstrated benefits such as higher growth rates [90]. Even without an internal heating system, a considerable amount of scaling has been achieved, amongst others, for a pressure variant working at much lower pressures around 100 MPa and likewise, with acidic mineralizers, as reported by K. Kurimoto et al. [84], but also for ammonobasic growth [98].

### 6.2. Reactor Lifetime and Crystal Purity

The reactor lifetime and the purity of the crystals grown in autoclaves, depends strongly on the chemical stability of the material which the inner surface of the autoclave is made of. Many materials which are stable in the acidic system do not maintain their stability in the basic system, like noble metals and Ni, Co, and Ni–Co alloys vice versa [147,314]. But Ag and Mo are stable in the Na-basic and NH_4_F-acidic environments [147,314]. S. Pimputkar et al. [147] and A.-C. L. Kimmel and E. Schlücker [314] published a good overview of the material solubility according to the used ammonia solution.

In general, three approaches for corrosion-resistant reactor designs can be distinguished: manufacturing the entire autoclave from a corrosion-resistant material, protecting the inner wall with a hermetically sealed liner (or coating), or using a pressure-balanced liner (capsule) [314]. The first approach faces the challenge of finding a corrosion-resistant material that is simultaneously able to fulfil all other requirements for a load-bearing pressure vessel material (outlined in Section 6.1 and the references therein). The second approach requires careful design and manufacturing considerations to realize a hermetic seal and avoid a variety of potential issues that originate from the liner being exposed to the internal pressure and the dimensional changes that the load-bearing parts undergo during operation, but hardly any detailed information is mentioned in the literature. The pressure-balanced liner or capsule concept as the third approach avoids these complications by balancing the pressure on the inside and outside of the liner. As a consequence, it is easier to implement and comes with the advantage of easy exchangeability of the liner, at the cost of less effective protection due to the possibility of fluid exchange between the interior of the liner and the gap between liner and inner autoclave wall. Consequently, pressure-balanced liners are often used if the liner material is ceramic (usually resulting in a large mismatch in thermal expansion coefficients between the load-bearing material and the liner, which also is a limitation for the applicability of coatings) [315,316], and if easy exchangeability is desired (as for exploratory synthesis of new materials or prospective intermediates). In spite of its limitations, the pressure-balanced concept is sufficient to prevent, for example, the rapid corrosion of nickel–base alloys by indium [200].

The further discussions in this section are based exclusively on the data from ammonothermal crystal growth of GaN, because for the other nitrides, no or little information is available. Besides the mineralizer, the source material used has an influence on the corrosive behavior of the solution as well [314]. However, as a first approximation, it can be assumed that the mineralizer (alkali metals and halides) has a stronger impact on the corrosive behavior of the solution than the source material. Accordingly, the following insights about the reactor technology should be transferable to other nitrides as well.

To improve on the corrosion resistance of the autoclave, some autoclaves are lined with an inert liner (commonly used in acidic systems) to prevent corrosion of the load-bearing material. Additionally, liners have been used to reduce contamination by elements released from the autoclave wall [314]. Extensive investigations have been performed on the stability of materials in ammonothermal environments, aiding in the development of liners and autoclave technology.

**Figure 17:**
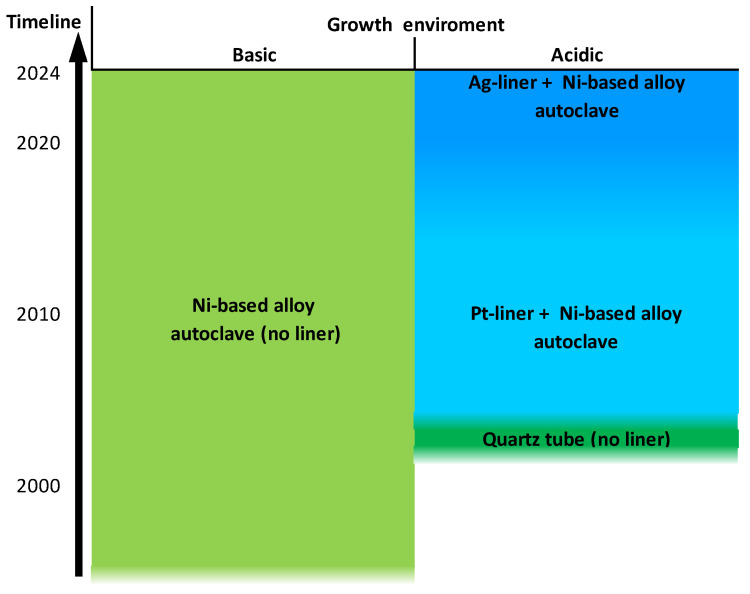
Chronological development of the autoclave material and liner technology, since the first publication of the basic and acidic growth environment by four selected groups that contributed to the development of ammonothermal growth over an extended period of time. For the ammonobasic growth environment, the groups IHPP/NL-3 and SixPoint/UCSB were selected and for the ammonoacidic growth environment, the groups Tohoku/MCC and NRL were selected. Every color is a chronological step. The greenish fields are chronological steps without a liner, the bluish with a liner: Ni-based alloy autoclave (no liner) [95,98,101,293,317], quartz tube (no liner) [320], Pt liner + Ni-based alloy autoclave [89,102,103,113,153,318,319], Ag liner + Ni-based alloy autoclave [84,156,242].

Generally, nickel–chromium alloys are used due to their high strength at high temperature, along with their excellent toughness properties. Most of these alloys are resistant to corrosion by ammonia under basic but not under acidic conditions [101,147,314]. Figure 17 offers an overview of the published autoclave technologies over time, based on four selected groups. The chronological development of the basic ammonothermal field is based on the publications of the group from Poland (IHPP/NL-3 [95,98,101]) and USA (SixPoint/UCSB [293,317]). The chronological development of the acidic ammonothermal field in Figure 17 is based on the publications of the group from Japan (Tohoku/MCC [84,89,102,103,113,153,156,158,242,318,319]) and the first publication on the acidic ammonothermal system from the USA (NRL [320]). We use these groups to show the chronological autoclave material and liner technology development, because:

- They more likely mentioned in their publications which materials they used, contrary to other groups;
- Their publication record is wide, from the first publication on the topic for the basic environment and nearly on the first publication from the acidic environment, until now (contrary to other groups).

**Autoclaves for ammonoacidic experiments:** The development of adapted autoclave materials for the ammonoacidic GaN crystal growth started in 2004 with the covering of the inner autoclave wall with Pt [153]. A. Yoshikawa et al. [153] mentioned the important role of the Pt liner for the prevention of possible contamination (Figure 17). The autoclave was likely a Ni-based alloy, according to the group’s later publications [153,319].

In 2014, B. Hertweck et al. demonstrated Ag as a coating for acidic ammonothermal conditions [321]. In their studies, Ag showed good corrosion resistance in the NH_4_F environment [321]. They pointed out the potential cost reduction with the usage of an Ag liner instead of a Pt liner [321]. Additionally, they also proposed that an Ag coating on a Ni-based alloy has good elastic properties and relaxation behavior, which results in a low stress condition between the two materials [321]. As mentioned by Bao et al. [103], the usage of NH_4_F instead of the other halides is beneficial for growth characteristics like purity and growth speed. According to A.-C. L. Kimmel and E. Schlücker [314], Pt is not stable in NH_4_F, at least not as stable as Ag. The use of an Ag liner for a Ni-based autoclave in combination with the mineralizer NH_4_F was first reported by D. Tomida et al. [156]. In their latest publication on ammonothermal GaN growth from early 2024, K. Shima et al. from the Tohoku/MCC group [242] still used the same combination of mineralizer and reactor materials. The combination of the beneficial mineralizer NH_4_F with the cost-reductive and soluble stable Ag liner in a Ni-based autoclave shows the advantage and reasonableness of this latest development step of the Tohoku/MCC group.

Parallel to the liner development of the Tohoku/MCC group, T. F. Malkowski et al. published two articles in 2016 and 2018 [164,322] regarding the usage of an unlined titanium–zirconium–molybdenum alloy (TZM) in the ammonoacidic environment. The advantages of Mo-based alloy autoclaves are the price reduction in comparison to the usage of a Pt-lined Ni-based autoclave [156], the technological simplification (due to the absence of a liner), and the potential of higher growth temperatures (>600 °C) [322]. The main disadvantage of these materials is their brittleness at low temperatures, which poses an increased safety risk [164]. Additionally, these materials have high yield strength so, accordingly, a higher load must be attended for sealing, contrary to Ni-based autoclaves [164].

In addition, the unique approach by Soraa Inc. (now KYOCERA SLD Laser, Inc.) [90] to autoclave technology, which deviates from the traditional sealed cylindrical tube geometry, should be mentioned. Their approach is based on the utilization of internal heaters located in-between the load-bearing autoclave walls and a sealed volume containing the ammonothermal solution. This approach is discussed in greater detail in Section 6.1.

**Autoclaves for ammonobasic experiments**: The development of the autoclave material for the ammonobasic GaN crystal growth is not clearly mentioned in the literature for the IHPP/NL-3 group. Usually, they only state that it is a Ni-based autoclave [95,98,101]. According to the publication by S. Sintonen et al. [146], the IHPP/NL-3 group developed a so called “next generation autoclave”. In their experiments, S. Sintonen et al. [146] grew one crystal in five growth steps and in three different autoclaves, which results in five layers/lamellae in one crystal (Figure 13). They performed the first growth run in a small older autoclave, grew the next two layers in a larger “next generation autoclave”, and finally, grew the last two layers in a third autoclave of the older generation [146]. For the two growth runs using the next generation autoclave, they observed an impurity concentration reduction in the order of one magnitude for impurities originating from the inner autoclave wall, contrary to their observations with an autoclave of the older generation [146].

Because Ni alloy autoclaves are more stable in basic ammonothermal fluids, compared to acidic ammonothermal fluids, fewer incentives exist to further refine the technology and include liners. In 2014 and 2016, a notable advance in the reduction of impurities originating from the inner autoclave wall by the usage of a Ag liner in an ammonobasic experiment was reported by S. Pimputkar et al. [140,323]. Nevertheless, even in the crystals grown in the ammonobasic system with a liner, some impurities originating from the autoclave wall were detected, specifically Mn, Fe, and Zn. This can be explained by the lack of a hermetic seal of the capsule. The use of a hermetically sealed capsule would likely have further reduced these concentrations [89,140,323].

### 6.3. In Situ Monitoring

Autoclave designs made for in situ monitoring purposes require modifications. These modifications include feedthroughs for thermocouples, window assemblies for incorporating optically transparent or X-ray transparent windows (in different locations as indicated earlier in Figure 15b–d), an autoclave designed as a rolling ball viscosimeter, and a rotatable feedthrough. Those technical developments will be described in the following paragraphs.

**Feedthroughs for thermocouples:** Thermocouples have been introduced either individually [127], or as a group of up to two [127,324]. For sealing, a blind made of a nickel–base alloy with a tight fit bore was used [127]. The space in-between the thermocouple sheath (likewise made from a nickel–base superalloy) and the bore wall was filled either by soldering or by welding, simultaneously providing a firm connection between the thermocouple sheath and the blind [127].

**Figure 18:**
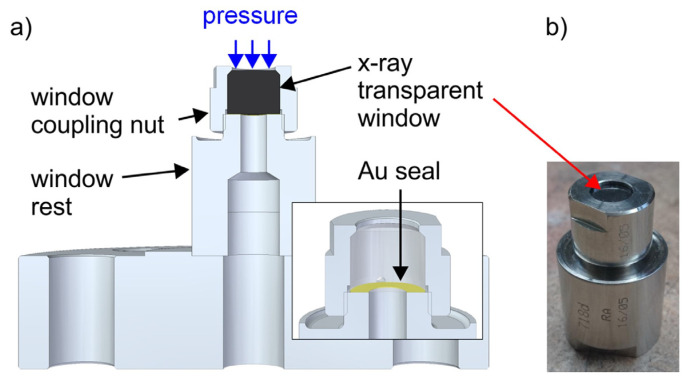
Window assembly with the flange for mounting it to an autoclave, as used for experimental access using optical light or X-rays of moderate photon energy (typically in the 40 to 100 keV range). (**a**) Computer-aided design (CAD) model in cross-section view, the inset shows a close-up of the sealing region and the exchangeable ceramic window displayed as transparent. (**b**) Photograph of window assembly equipped with a boron carbide window. Reproduced from [299].

**Window assemblies:** The autoclave windows represent optical windows of the Poulter type, distinguished by enhanced contact force under internal pressure and relatively low susceptibility to stress distribution irregularities caused by tolerances [297]. The seal between the window and the window rest is achieved by plastic deformation of a gold layer. Since most measurement methods require two optical windows, these autoclaves must include a cross hole for connecting peripheral devices (such as a hand valve, pressure transducer, thermocouple, and burst disc). Both the cross hole itself and the mechanical stresses induced in the autoclave body during peripheral screwing necessitate an increase in wall thickness [299]. To mount the window in the optical cell, specialized components made from Inconel 718 are employed, instead of a conventional cover flange, as illustrated in Figure 18. The process of assembling the window involves applying a compressive force of 30 kN, which remains constant during the phase of contact pressure. This sustained pressure enables the gold layer to deform plastically, effectively filling any surface irregularities and ensuring a secure seal [299].

While considering suitable materials for the windows of autoclaves employed in ammonothermal processes, the primary choices can be narrowed down to single-crystalline sapphire and ceramic boron carbide (B_4_C).

Sapphire, particularly when oriented along the a-axis, is favored for its mechanical robustness, making it less susceptible to structural failures under high-pressure conditions. Moreover, sapphire exhibits supreme optical transparency, facilitating the transmission of light across a wide spectral range, which is advantageous for optical measurements and spectroscopic analyses. However, the mechanical strength of sapphire windows can depend on crystal orientation and temperature. Sapphire windows typically undergo extensive factory polishing procedures to attain the requisite surface smoothness and parallelism necessary for optimal functionality in high-pressure environments.

Although B_4_C does not exhibit the same degree of optical transparency as sapphire, B_4_C showcases exceptional transparency to X-rays, rendering it well-suited for applications involving X-ray analysis or imaging. Furthermore, B_4_C represents a polycrystalline ceramic material, potentially conferring advantages in terms of cost-effectiveness and manufacturability, compared to single-crystalline sapphire. Nonetheless, the polycrystalline nature of B_4_C may lead to marginally lower mechanical strength and optical quality in comparison to sapphire windows.

Additionally, the chemical stability of both materials in diverse ammonothermal environments needs careful examination. While sapphire demonstrates remarkable resistance to chemical corrosion and degradation across various conditions, the chemical stability of B_4_C may exhibit variability with respect to the specific chemical composition, and the pH value of the ammonothermal solution. Hence, thorough evaluation of the intended application and environmental parameters is required when selecting the appropriate window material for optical cells used in ammonothermal processes [299].

Avoiding notches, particularly in the central portion of the window, is crucial to prevent the notch effect, which can lead to window breakage upon insertion into the sealing gold layer, or during operation. Additionally, maintaining low surface roughness in the sealing area is vital to ensure that the plastically flowing gold layer adequately fills any remaining irregularities during sealing. While the precise impact of shape and position tolerances of the upper bevel of the viewing windows on window longevity or assembly success is not fully understood, visible deviations from the target geometry and the occurrence of cracks, often originating from the contact area between the window holder and the bevel, suggest that these tolerances may contribute to localized stress concentrations. Thus, attention to detail in the manufacturing process and adherence to specified tolerances are critical to ensure the integrity and longevity of the window assembly [299].

**Figure 19:**
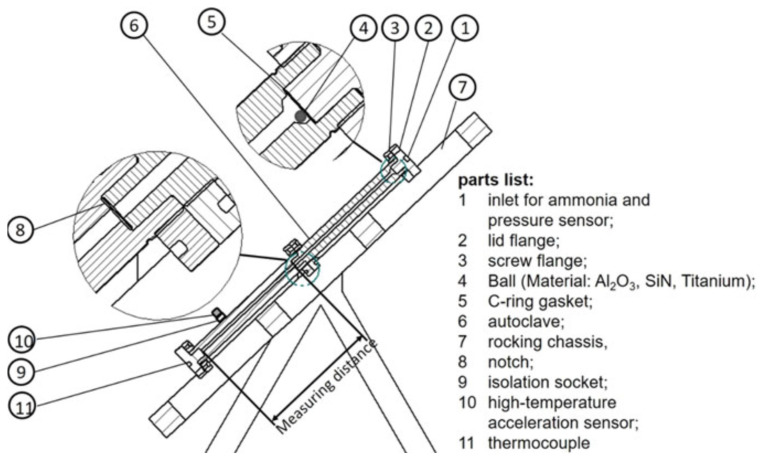
The illustration of the rolling ball viscometer engineered for accuracy in measuring viscosity across different fluid conditions. Reproduced from T.G. Steigerwald et al. [325] with permission, © Springer Nature Switzerland AG 2021.

**Rolling ball viscosimeter:** A rolling ball viscometer, shown in Figure 19, was fabricated from a nickel-based superalloy, Inconel 718 [325]. It was designed and rigorously tested for a maximum temperature and pressure of 600 °C and 300 MPa, respectively. Design features specific to viscometry were the aspect ratio of the inner volume (realized by connecting two autoclaves), and a special conical shape at both ends to ensure that the ball only starts to roll at a certain angle of inclination [325]. A specific technical challenge lies in the ball itself, which should be as light as possible to maximize rolling time, but should also be resistant to corrosion and be available in spherical geometry [325].

**Figure 20:**
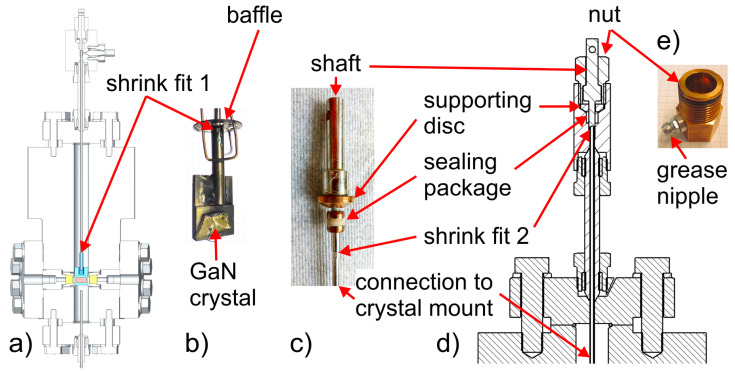
Rotatable feedthrough for ammonothermal autoclaves: (**a**) implementation in an autoclave designed for in situ diffraction experiments (same type of autoclave as in Figure 15d), (**b**) photo of a GaN crystal fixed to a crystal mount, which in turn is connected to the rotatable feedthrough; (**c**) photo of the shaft with supporting disc, sealing package, and shrink fit connection to the drive shaft passing on the torque to the crystal mount, (**d**) drawing of the feedthrough mounted to the cover flange, (**e**) photo of nut with grease nipple to reduce friction to a manageable level. Reproduced from [299].

**Rotatable feedthrough:** For realizing X-ray diffraction measurements (with monochromatic radiation) on GaN crystal during ammonothermal growth, a rotatable feedthrough for ammonothermal process conditions was also developed [299,326], which was used in conjunction with the autoclave shown in Figure 15d. A CAD model of the respective autoclave, with windows implemented at the diffraction angle to be used, is shown in Figure 20a, while a photograph of the part holding and rotating the crystal is depicted in Figure 20b. A first challenge lies in the high demands on the gasket to withstand the pressure of 300 MPa, while allowing for the movement (grinding seal). To avoid the simultaneous requirement of withstanding temperatures of up to 600 °C, the grinding seal was positioned outside the actively heated region so that PTFE could be used for the sealing package (see Figure 20c). A second challenge lies in transferring the torque from the shaft to the remotely located GaN crystal, which required a shrink fit connection to join the separately manufactured shaft to a long thin nickel–base alloy tube, and a second shrink fit connection between the tube and the crystal mount. The overall assembly, except for the crystal mount, is depicted in Figure 20d.

**Windowless autoclaves with improved X-ray transparency:** In addition to the above developments, which have been tested experimentally under ammonothermal process conditions, the use of adapted reactor designs in conjunction with computed tomography with high photon energies has been estimated to be a feasible means of enabling in situ monitoring of crystal growth, even in windowless autoclaves [304]. An encouraging factor beyond that published feasibility study is that other application fields of superalloys would likewise benefit from superalloys with lower density [327], suggesting that the availability of suitable alloys with improved X-ray transparency may improve further in the future.

## 7. Emerging Technologies for In Situ Monitoring

For several reasons, ammonothermal reactors are challenging systems to obtain information from during operation. Firstly, the high pressures necessitate thick metal reactor walls, which lack optical transparency. Secondly, the corrosiveness of most ammonothermal reaction media towards most construction materials [147,148,315] strongly limits the choice of materials for windows with improved transparency for either optical light or X-rays [148]. Consequently, insights via in situ monitoring or numerical simulations are highly desirable.

Despite the above-outlined challenges, in situ monitoring techniques have been developed and have proved insightful for gathering information on the physical properties of the fluid and crystal growth/dissolution characteristics [328]. Understanding the progression of the reaction is vital for enhancing crystal quality in a targeted way. Real-time observation of the reaction offers invaluable insights into the system, surpassing post-reaction analyses. For instance, it can be difficult to unambiguously link distinguishable layers to process steps post-run [329].

In the field of ammonothermal crystal growth, numerical models have only been developed to a limited extent, and discrepancies often arise between simulation outcomes and actual experimental findings. One significant challenge lies in the difficulty of directly validating simulation results against experimental data. This difficulty stems from the intricate nature of ammonothermal experiments, which makes it challenging to measure crucial parameters. For instance, internal temperatures can only be measured for a limited number of measurement locations per reactor, and flow velocities have so far only been observed indirectly via their impact on fluid temperatures [127]. Unlike hydrothermal crystal growth, which benefits from well-established connections with geological research, the ammonothermal growth of nitrides lacks such a robust foundation [128]. Consequently, there remains a considerable gap in our understanding of the underlying thermodynamics, kinetics, and fluid properties specific to ammonothermal systems. As a result, the assumptions made in simulations carry a heightened risk of inadequacy due to the lack of validation and lack of accurate thermophysical data [128].

For instance, fluid properties play a pivotal role in ammonothermal processes, particularly considering the use of supercritical ammonia as a solvent. Data on the properties of pure ammonia are available [131], but the introduction of decomposition products and solutes can significantly alter these properties [330]. Experimental investigations focusing on thermophysical properties of ammonothermal fluids would provide invaluable insights into the intricacies of ammonothermal systems [128].

Viscosity, for example, is a critical factor in processes like ammonothermal synthesis. Its prediction is complicated by variables such as pressure, temperature, and dissolved substances altering fluid properties. While familiar fluids like water and oils typically exhibit Newtonian behavior, more complex ones like polymer melts behave non-Newtonian. In the case of supercritical ammonia used in ammonothermal processes, initial assumptions lean towards linear viscous flow resembling gases according to kinetic gas theory. However, this simplification overlooks the departure from ideal gas behavior under high pressures. To estimate viscosity in such conditions, Sutherland’s semi-empirical formula is employed, although it has limitations, especially concerning high pressures and mixtures of substances [331,332]. Understanding viscosity in solutions adds another layer of complexity. The widely used Jones–Dole equation, applied to describe viscosity in aqueous solutions, considers factors such as concentration and ion interactions [333]. However, predicting behavior becomes more intricate with solutions containing multiple components. For example, the impact of ammonium chloride in water can vary depending on factors like the presence of other substances, such as urea or ethanol [334]. Such complexities underscore the difficulty in making broad predictions for solution viscosity. Consequently, in the realm of ammonothermal synthesis, assessing viscosity in situ becomes crucial due to the intricate interplay of factors affecting fluid behavior [335].

Furthermore, the decomposition of ammonia during crystal growth [136] introduces additional complexities, such as variations in ammonia mole fractions and the influence of catalysts on decomposition kinetics. Understanding these factors is essential for modeling the ammonothermal growth process with sufficient confidence in model accuracy. Moreover, uncertainties persist regarding the optical characteristics of the solution and the possible presence of nanoparticles, both of which can affect heat transfer and convection within the system [128]. Additionally, the impact of solutes on heat capacity remains unclear, as does the accurate determination of solubility data under varying conditions [128,150,151,152]. The establishment of comprehensive databases containing solubility information across different parameters would greatly enhance the reliability of numerical simulations, while also supporting experimentalists in designing their experiments. Furthermore, while some data exist on kinetics, particularly dissolution [152] and growth kinetics [324], they remain limited. Addressing these detailed uncertainties, together with improved model validation, is crucial for advancing the accuracy and reliability of simulation outcomes [128]. Thus far, mainly X-ray imaging [150], optical methods like UV/Vis or Raman spectroscopy [336], and to some extent, ultrasonic viscometry [337], have been applied for in situ monitoring of chemical or physical processes occurring in ammonothermal reactors under conditions of ammonothermal growth processes. In addition, the feasibility of X-ray computed tomography has been evaluated [304]. These techniques are listed in Table 7, alongside the types of information that they are able to provide.

Given that each technique has different strengths and limitations, a synergistic application of several techniques is often beneficial for an unambiguous interpretation of the results. In the following, key aspects of the respective techniques in the context of their application to the ammonothermal method will be delineated, and an overview of the state of the art will be given for each technique.

### 7.1. Internal Temperature Measurements

Thus far, thermocouples have been the device of choice for measuring temperatures inside ammonothermal reactors during operation, due to their comparatively convenient integration into ammonothermal autoclaves (see Section 6.3). Initial experiments concerned with investigating the effects of fluid temperatures on crystal growth kinetics in supercritical ammonia–sodium (NH_3_–Na) solutions were conducted by S. Griffiths and coworkers [324], who evaluated the mass change of source material and seeds in combination with the fluid temperatures to better understand the crystal growth process. The primary focus of the experiment was to delve into the intricate interplay between internal fluid temperatures and crystal growth rate in supercritical NH_3_–Na solutions. By measuring temperature changes in both the dissolution and growth zones, the team aimed to understand the mechanisms behind crystal growth and find the best conditions for maximizing growth rates. Inconel-sheathed type K thermocouple probes were placed within the autoclave. One of them was positioned above the polycrystalline GaN source material in the dissolution zone and the other near the top-most seed crystal in the growth zone. The setup was designed to accommodate thermocouples without disrupting fluid flow, using smaller diameter probes, and including open areas in the baffled region. Furthermore, a comprehensive redesign of the loading procedure and hardware setup was undertaken to enable the lower thermocouple probe to reach the growth environment. This thermocouple was carefully positioned near the top-most seed crystal, just below the bottom-most baffle. The other thermocouple probe was situated in the dissolution zone, directly above the polycrystalline GaN source material. For both growth and dissolution zone, they evaluated not only the internal temperatures, but also the mass changes of GaN normalized by surface area and time (termed source loss flux and seed mass flux, respectively). In addition, they analyzed the fluid density difference between the dissolution and growth zone [324].

**Figure 21:**
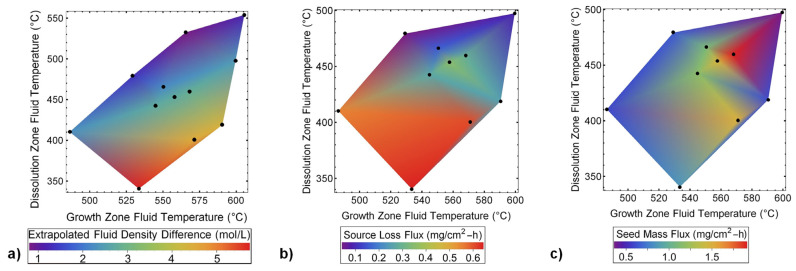
Quantities determined as a function of growth zone and dissolution zone temperatures by internal temperature measurements for investigating the role of surface kinetics and mass transport for crystal growth rates of GaN using Na mineralizer. (**a**) Extrapolated fluid density difference, (**b**) source loss flux, and (**c**) seed mass flux. Reprinted with permission, © 2018 Elsevier B.V. [324].

The aforementioned quantities are presented in Figure 21. From comparison of subfigures (a) and (b), one can see that there is a strong positive correlation between fluid density difference and source loss flux, particularly for low fluid density differences or low dissolution zone temperatures. S. Griffiths et al. determined a critical ammonia density difference of about 1.2 mol/L, above which transport from dissolution to growth zone, but also parasitic deposition occur [324]. Above this critical ammonia density difference, source mass flux increased with decreasing dissolution zone temperature, confirming retrograde solubility of GaN in NH_3_–Na solutions [324]. Considering the temperatures and pressure, the overall ammonia density can be estimated to be about 20 mol/L [136], thus, the determined critical density difference corresponds to about 6%.

A comparison of Figure 21b,c reveals that peak source loss flux does not correspond to peak seed mass flux, indicating that for most conditions, seeded GaN crystal growth is limited by surface reaction kinetics, rather than by the transport from dissolution to growth zone [324]. Accordingly, S. Griffiths et al. determined endothermic activation energies of 145, 136, and 191 kJ/mol for the (0001), (000-1), and {1 0-10} surfaces, respectively, for growth zone temperatures below 570 °C. Above 570 °C, a reduction in seed mass flux with increasing growth zone fluid temperature was observed, which S. Griffiths et al. speculate to be related to changes of the fluid composition at high temperatures [324].

Besides the absolute fluid temperatures, S. Griffith et al. also mentioned fluctuations of fluid temperatures, which were in the range of 1 to 10 K in the growth zone and in the range of 5 to 15 K in the dissolution zone [324]. Considering that they mention that these fluctuations primarily depended on the heater setpoints (for the growth zone at the bottom of the autoclave) and on the insulation of the autoclave head (for the dissolution zone in the upper part of the autoclave) [324], these fluctuations are very likely related to fluid flow, which is also strongly influenced by both of these factors [338].

**Figure 22:**
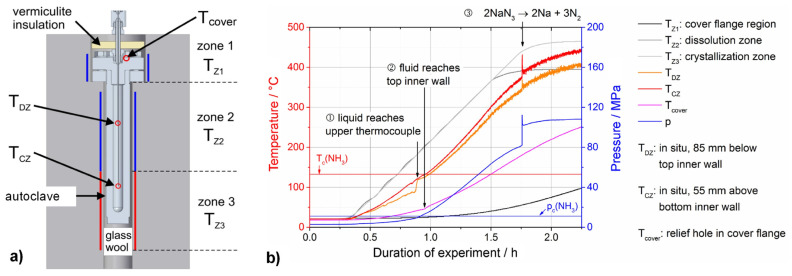
(**a**) Experimental setup consisting of a tube-shaped windowless autoclave equipped with two internal thermocouples for measurements in the dissolution zone (DZ) and the crystallization zone (CZ); (**b**) temperature and pressure measurements recorded in the process of temperature increase from room temperature to growth conditions (the critical temperature T_c_ and the critical pressure p_c_ of ammonia are also indicated, and events that can be observed via the internal temperatures are labeled). Reprinted from [127] under open access Creative Commons CC BY 4.0 license.

Following comparable observations, Schimmel et al. explored the potential of in situ fluid temperature measurements as indicators of flow dynamics [127]. The investigation involved carefully monitoring temperature fluctuations within the autoclave, particularly focusing on how these fluctuations can be applied as indicators of flow velocity changes. A schematic of one of the experimental setups is depicted in Figure 22a. Both experimental setups, which differed in the length of the autoclave, were equipped with one thermocouple in the upper and one in the lower zone. By analyzing temperature data collected under different experimental conditions, such as the presence of baffles and variations in autoclave geometry, they aimed to decipher the underlying convective heat transfer mechanisms. In Figure 22b, several types of information that can be obtained are denoted, using the first part of an experiment in which the temperature was increased from room temperature to elevated temperatures, with the higher temperatures being reached in the bottom part of the autoclave to facilitate natural convection. The fluid temperature measurements (T_DZ_ for the dissolution zone in the upper part of the autoclave and T_CZ_ for the growth zone in the lower part of the autoclave) gradually developed the before-mentioned short-term fluctuations (broadening of the respective lines in the graph). Furthermore, changes in heat transfer, due to the fluid reaching the thermocouple and subsequently the top inner wall, could be detected. Likewise, the decomposition of the mineralizer precursor sodium azide was detectable, both as a sudden temporary jump in fluid temperatures and as a pressure jump [127].

Numerical simulations were employed to complement experimental findings, shedding light on the response times of thermocouples and their impact on temperature measurements. Considering that flow velocities in the proximity of the growing crystals appear to have significant effects on crystal quality [138], this holistic approach promises not only to enhance our understanding of fluid flow dynamics in ammonothermal crystal growth, but also to provide valuable insights for optimizing growth conditions and ensuring consistent crystal quality [127]. From a methodological standpoint, a crucial consideration is how deeply an internal thermocouple must penetrate the fluid to accurately measure its temperature, without being unduly influenced by wall temperatures due to heat conduction within the thermocouple itself. Based on simulation results indicating a deviation of 15 K or less for a thermocouple inserted a few millimeters into the fluid, it becomes evident that significant thermal gradients between the autoclave wall and the fluid can affect temperature measurements [127].

In previous studies utilizing an optical cell for various measurements, including optical and X-ray absorption studies, thermocouples positioned near the inner wall of the autoclave did not typically exhibit temperature fluctuations, particularly during the main phases of the experiment [148,149,336]. This absence of fluctuations is primarily attributed to the designed isothermal conditions of the optical cell, minimizing convective heat transfer effects. However, it was possible to induce temperature fluctuations in the optical cell by removing the heating sleeve during cool-down, confirming that a distance of approximately 2 mm between the thermocouple tip and the autoclave wall is sufficient for detecting convection. This insight underscores the suitability of internal temperature measurements as probes for convective flow dynamics in experimental setups. Practicality states that the maximum deviation of 15 K is unlikely to manifest, given the typically small axial thermal gradients within the autoclave wall, which mitigate significant thermal differentials between the wall and the fluid. Therefore, the positioning of thermocouples within the fluid, even near the autoclave wall, remains a reliable method for monitoring convective heat transfer [127].

### 7.2. Imaging Techniques

In the area of imaging techniques, both the use of optical light and X-rays have been tested. As will be elaborated in the respective sections, these techniques differ in the range of ammonothermal process conditions in which they can be used, their requirements for autoclave window materials, and the information that can be obtained.

#### 7.2.1. Video Optical Measurements

**Figure 23:**
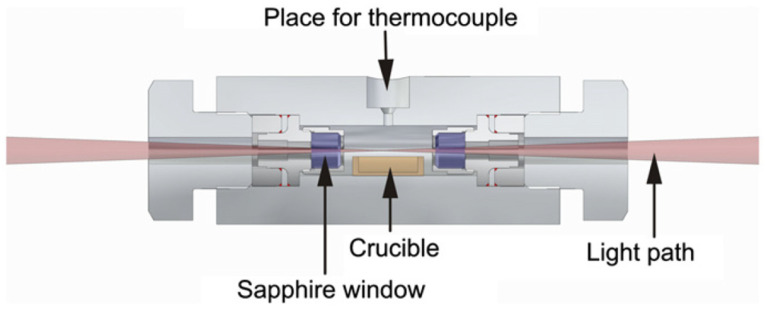
Sectional view of optical cell setup with positions for thermocouple and passage for light. Reproduced from [342] with permission, © 2011 Elsevier B.V.

With sapphire as the optically transparent material being a usual choice for pressure vessel windows [339,340,341], video optical measurements were among the first imaging techniques investigated for application to ammonothermal processes. The construction of the first optical cell developed for ammonothermal conditions [298] can be seen in Figure 23. This optical cell was made from Inconel 718 alloy (a nickel–base superalloy) with sapphire windows at both ends to provide a straight optical path through the cell. This optical cell was designed for pressures as high as 200 MPa and temperatures up to 650 °C.

**Figure 24:**
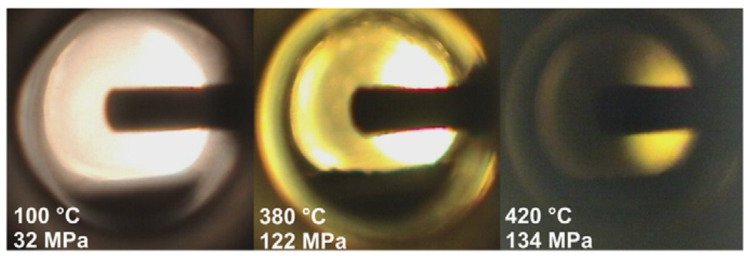
Video optical measurements of ammonoacidic GaN system at increasing temperatures showing decrease in visibility of the internal system [342]. The part extending into the area of view is the tip of the thermocouple. Reproduced from [342] with permission, © 2011 Elsevier B.V.

This optical cell was equipped with a type K thermocouple that extended from the side wall into the reaction chamber, so that fluid temperatures could simultaneously be monitored [298]. For the video optical measurements, a halogen light source was used in conjunction with a standard video camera for recording, and the thermocouple tip served as an object to focus on inside the reaction chamber [298,342]. Ammonia, ammonium chloride, and polycrystalline gallium nitride powder were used as starting materials. It is worth noting that the experimental setup used provides independent control of pressure and temperature, which is achieved utilizing two high-pressure pumps for fill level adjustments during operation [298]. This enables operational flexibility and adjustment of reaction parameters beyond the thermodynamic stability field [298]. Examples of obtained video-optical measurements are depicted in Figure 24. For temperatures up to 350 °C, the video-optical measurements allowed monitoring of the movement of GaN particles, including the velocity and direction of the fluid flow [298]. An axial circulatory flow was clearly detectable, with flow speeds in the order of millimeters per second [298]. As is expectable for the optical cell as a near-isothermal system, the observed order of velocity magnitude corresponds to that found in numerical simulations of ammonothermal autoclaves for crystal growth, if the simulations consider conditions of low driving force for convection during the etch-back process prior to growth (dissolution stage) [329]. At 370 °C, the fluid turned yellow and then, with increasing temperature, became more opaque, leading to barely any light passing through the system at 420 °C [342]. This demands the use of other in situ monitoring techniques for ammonothermal systems under conditions of crystal growth, particularly if high solute concentrations are present in the location of measurement [342]. In spite of this limitation, it is remarkable that video-optical measurements remain the only technique that has been reported to yield quantitative information of fluid flow velocities in ammonothermal autoclaves to date.

#### 7.2.2. X-ray Imaging and Computed Tomography

Contrary to optical light, X-ray absorption is not as heavily affected by the presence of solutes that are formed upon the dissolution of GaN. The remaining X-ray intensity after penetration of an object *I* depends on the initial intensity *I*_0_, the length *t* of the radiographed path through the object, the linear absorption coefficient *µ* of the radiographed material, and the density *ρ* of the radiographed material (or alternatively the concentration, if the absorbing element is a solute). This is evident from Lambert–Beer’s law (Equation (2)) [343]:

(2) $$I=I_{0}\cdot e^{-\frac{µ}{\rho }\cdot \rho \cdot t}$$

For objects to be distinguishable in an X-ray image, the objects need to differ sufficiently in their X-ray attenuation to yield a visible contrast in the image. As a measure of contrast, the Michelson contrast *C_m_* is simple and sufficient for our purposes [344], though it overestimates the contrast of dark pairs of points compared with bright pairs with the same level of gray [345]. The Michelson contrast is defined as follows (assuming that *I_max_* represents the maximum X-ray intensity reaching the detector and *I_min_* represents the minimum X-ray intensity reaching the detector [300]):

(3) $$C_{m}=\frac{I_{max}-I_{min}}{I_{max}+I_{min}}$$

**Figure 25:**
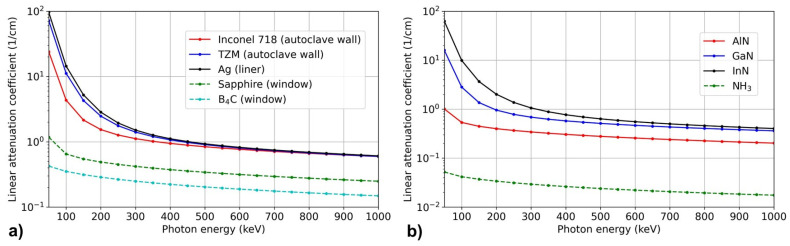
Linear attenuation coefficients plotted against photon energy for (**a**) construction materials of the autoclave and (**b**) educts involved in the ammonothermal synthesis. Mass attenuation coefficients for calculating the linear attenuation coefficients were taken from the Photon Cross Sections Database XCOM of the National Institute of Standards and Technology (NIST) [346]. For ammonia, a density of 0.23395 g/cm^3^ was considered.

Consequently, for a crystal to be distinguishable from the reaction medium, their linear attenuation coefficient or radiographed path must be sufficiently different, for which the linear attenuation coefficient usually plays a dominating role. In Figure 25, the linear attenuation coefficients of different materials relevant to ammonothermal experiments are shown as a function of photon energy. Note that the linear attenuation coefficient µ is the product of the density *ρ* and the mass attenuation coefficient *µ*/*ρ*, and therefore already accounts for the element- and density-specific contributions to X-ray absorption. The general trend is that the linear attenuation coefficients decrease with increasing X-ray energy, motivating the use of intermediate to high X-ray energies if the intensity that reaches the detector is a main limitation. An extreme example of this is the prospective application of X-ray computed tomography with acceleration voltages of several hundred volts to penetrate windowless autoclaves designed for crystal growth [304].

In the left subfigure of Figure 25, different construction materials typically used for ammonothermal reactors are compared with regard to their linear attenuation coefficients. It can be seen that regardless of the photon energy, the use of sapphire or boron carbide represents a major improvement in linear attenuation coefficient, compared to the nickel–base superalloy Inconel 718, the molybdenum–base alloy TZM, or the liner material silver. Moreover, B_4_C yields an even lower linear attenuation coefficient than sapphire. More generally, materials that consist of light elements and have low density are the best choices for window materials if X-ray absorption is to be minimized, including materials like vitreous carbon, diamond, or silicon carbide [148]. However, the additional requirements of chemical stability under ammonothermal conditions and availability of a sufficient thickness to withstand the pressure need to be considered as well. Vitreous carbon exhibited significant damage in solutions containing sodium azide, though only exhibited superficial damage when tried for ammonoacidic conditions. Silicon carbide remained unaltered under ammonobasic conditions, but noticeable corrosion damage occurred under ammonoacidic conditions. From a chemical stability point of view, diamond is the ideal window material, as it withstands various ammonothermal environments, however, it has not yet been applied as a load-bearing window material due to the very limited availability and high cost of thick diamond materials [148].

**Figure 26:**
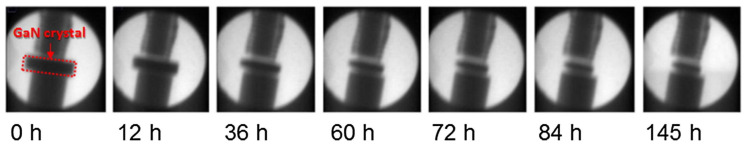
X-ray images showing the dissolution of a GaN crystal during an ammonothermal experiment conducted using an optical cell. Below and above the GaN sample, the Inconel 718 pins of the crystal mount are visible. Reproduced from [149] with permission, © 2017 Elsevier B.V.

In the right subfigure of Figure 25, linear attenuation coefficients of different binary nitrides, as well as pure supercritical ammonia, are provided. It is evident that the linear attenuation coefficient of ammonia is much lower than that of any of the binary nitrides, particularly for InN and GaN. This explains the good contrast between ammonia and GaN that is generally observed. An exemplary series of X-ray images obtained during an ammonothermal dissolution experiment with GaN is depicted in Figure 26.

**Figure 27:**
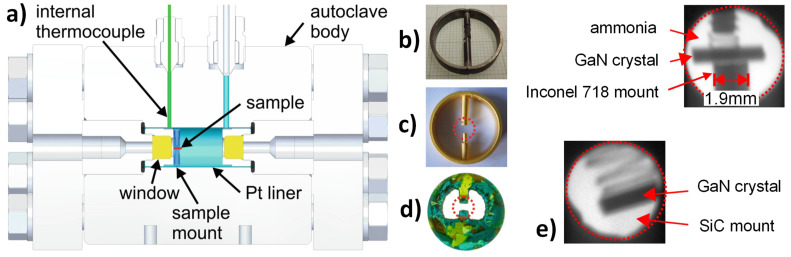
(**a**) Optical cell fitted with transparent windows for X-rays, (**b**) sample mount from Inconel 718 (uncoated), (**c**) sample mount from Inconel 718 (gold-coated), (**d**) sample mount from polycrystalline SiC grown via physical vapor transport, and (**e**) X-ray images obtained with crystal mounts made of Inconel 718 (**top**) and SiC (**bottom**). Regardless of the mount material, an Inconel 718 spring is used in addition, to hold the sample in place (visible above the samples in subfigure (**e**)). The dashed red circles in subfigures (**c**–**e**) indicate the area of view that can be monitored by X-ray imaging. Adapted from [299].

For a better understanding of the images shown in Figure 26, imagery depicting a uniaxial optical cell, as used for obtaining the X-ray images, is shown in Figure 27. One can see that the area of view is somewhat small in relation to the inner diameter of the autoclave. Sample holders (Figure 27b–d) are needed to properly position the sample in the volume so that it can be observed. To mitigate corrosion, a gold coating was used for ammonoacidic (Figure 27b), whereas uncoated Inconel 718 for ammonobasic conditions (Figure 27c,d) shows a mount made of SiC, which yields good X-ray contrast to GaN, as can be seen by comparing the top and bottom images shown in Figure 27e [299]. This experimental setup was used for the majority of investigations on solubility and dissolution kinetics of nitrides via in situ X-ray imaging thus far.

By equipping optical cells with sapphire or boron carbide windows, 2D X-ray imaging becomes feasible with photon energies in the range of 40–100 eV [149,150,330]. Note that beam hardening plays a nonnegligible role for the mean energy of photons that reach the detector and therefore contribute to the image: the white X-ray spectrum (consisting of characteristic radiation and bremsstrahlung) undergoes a shift to higher photon energies as the lower energy photons more likely to be absorbed by the setup. For an acceleration voltage of 100 keV and two sapphire windows of 10 mm thickness each, the mean effective photon energy has been estimated to be about 50 keV [330]. Using as low photon energies as possible is particularly advantageous if contrast is a critical limitation, as lower X-ray energies provide better contrast between different materials. This is due to the fact that the linear attenuation coefficients of materials differ more significantly for low photon energies, which can be seen in both subfigures of Figure 25.

Given the high linear attenuation coefficients of the alloys from which the autoclaves are made (examples shown in Figure 25 are the nickel–base superalloy Inconel 718 and the molybdenum–base alloy TZM), much higher X-ray energies are required if the use of autoclave windows shall be avoided (for instance for thermal field and thermal inertia engineering or to overcome their limitations, such as the small area of view). To penetrate the walls of an Inconel 718 autoclave designed for up to 300 MPa operating pressure at up to 600 °C, high X-ray energies above about 300 keV are required [304,344], and even higher X-ray energies were found to further improve quality of the X-ray images [304].

**Figure 28:**
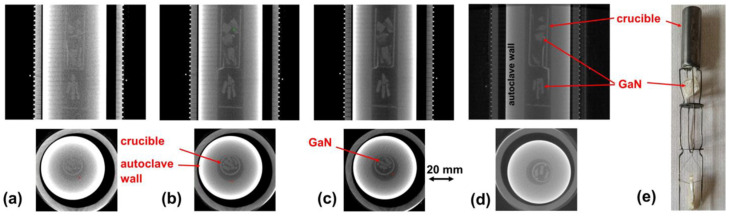
Computed tomography images of an autoclave interior. The (**a**–**c**) images are Inconel 718 autoclaves while (**d**) is Haynes alloy. X-ray energies are (**a**) 300 kV, (**b**) 550 kV, (**c**) 600 kV, (**d**) 590 kV. The photograph in image (**e**) shows the internal furniture including the GaN crystals. The internal furniture is made of Inconel. Reproduced from [304] under open access Creative Commons CC BY 4.0 license.

In Figure 28, exemplary images obtained using X-ray energies in the range of 300 to 600 keV are depicted. Note that these are computed tomography (CT) images, and these were obtained by capturing X-ray projections from multiple angles around the autoclave, in contrast to 2D X-ray imaging, which relies on a single projection. The use of many projections and projections obtained by radiographing the object in various directions yields significantly better image quality (after reconstruction) [344]. Consequently, S. Schimmel et al. have confirmed the feasibility of applying high-energy computed tomography for tracking the ammonothermal growth process of GaN and other materials of comparable X-ray absorption [304].

**Figure 29:**
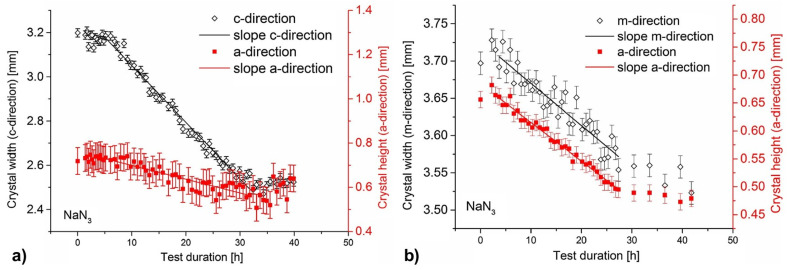
GaN crystal–dissolution kinetics with sodium azide mineralizer for (**a**) polar and non-polar orientations (c- and a-directions), (**b**) non-polar orientations (m- and a-directions). Reproduced from [149] with permission, © 2017 Elsevier B.V.

Up to now, 2D in situ X-ray imaging has primarily been used for studying both kinetic and thermodynamic aspects of GaN dissolution. Dimensional changes in a series of images like the one depicted in Figure 26 directly reveal the dissolution velocities for the crystallographic directions aligned perpendicular to the path of rays. Consequently, information on dissolution kinetics is straight forward to obtain for two crystallographic directions at a time [149,150]. From the left subfigure of Figure 29, it can be seen that the dissolution velocity (slope in the decrease of crystal width over time) depends on the crystallographic direction or, more specifically, the dissolution velocity in the c-direction is different from that of the a-direction. From the right subfigure of Figure 29, one can see that the dissolution velocity differences between the two non-polar directions (m and a) are negligible. The near-identical dissolution velocities in two crystallographic directions open a simple and therefore robust means of estimating the dimension in the third direction, which is not observable as a contour in 2D imaging. Provided that the third dimension represents one of the non-polar directions, it can be assumed to behave in the same way as in the directly observable direction [149,150]. While the dimensional change of the crystal in the direction of the path of rays can, in theory, be obtained from the changes in grayscale values in the respective region of the image, the outlined approach avoids errors that can occur, amongst others, due to changes in fluid absorption before or behind the crystal.

**Figure 30:**
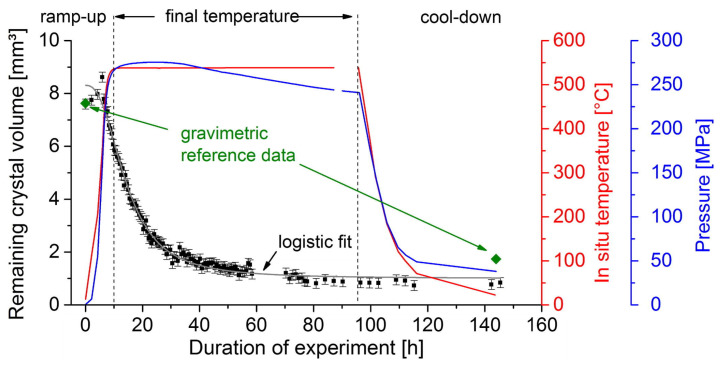
Change of GaN crystal volume over time shown alongside internal temperature and pressure. This ammonobasic experiment was conducted with 3 mol% NaN_3_ mineralizer, corresponding to a molar concentration of 0.72 mmol/mL. Reproduced from [149] with the permission, © 2017 Elsevier B.V.

Using the outlined methodology, information on the sample volume as a function of time can be obtained (an exemplary such dataset is shown in Figure 30), which is necessary for investigating solubility. A second prerequisite is to observe the saturation of the solution. As can be seen from the remaining crystal volume in Figure 30, the dissolution slows down and eventually no further dissolution is observed (unless unintended factors like thermal gradients lead to deposition of GaN elsewhere in the reactor, therefore the geometry of the uniaxial and biaxial cells is ideal for the purpose of solubility measurements, see Section 6.3).

Such studies of dissolution kinetics and solubility were carried out for selected ammonobasic (NaN_3_) and ammonoacidic conditions (NH_4_F, NH_4_Cl), with similar observations conditions [149,150,330]. Solubility data for GaN under different ammonothermal conditions, obtained by different methods of measurement, remain controversial and incomplete. Data have been obtained for some mineralizers, but temperature-dependent data for constant ammonia density are partially lacking, and the absolute magnitudes of solubilities remain inconclusive. A summary of all published data, as of 2021, can be found in [128].

The spatial resolution (both lateral and in the third, axial dimension), as well as the time resolution of 2D X-ray imaging of GaN in optical cells, has also been evaluated [299]. Since several subsequently taken X-ray measurements were commonly averaged for noise reduction and the air-cooled X-ray source allows only for a limited number of exposures shortly after each other [150], the achievable temporal resolution depends on the requirements for the quality of the images. Common time intervals between different series of measurements are in the range of 20 to 60 min [299,330]. While absolute positions are difficult to measure, changes of positions and dimensions can be determined rather accurately, with a lateral resolution of about 15 µm [299]. Regarding the axial resolution, changes in linear attenuation coefficient as small as about 0.12 cm^−1^ are detectable, which corresponds to Ga concentration changes of 0.13 mmol/mL [330].

Besides dimensional changes of GaN crystals, changes in X-ray absorption of the fluid can also yield valuable information. An observed increase in absorption of the solution revealed an increase in the concentration of dissolved gallium-containing species, and a local inhomogeneity in concentrations was observed [330]. A sphere-like region appeared around the crystal that slowly grew and diminished over time, suggesting slow dissolution and diffusional transport of Ga away from the crystal via formation and diffusion of intermediates [330]. The observed slow diffusion hinted towards an unexpectedly low diffusion constant of dissolved Ga complexes, which was at least partially explained by to the diffusion of larger [Ga_x_F_y_]^3x−y^ aggregates, on the grounds of molecular dynamics simulations [330].

**Figure 31:**
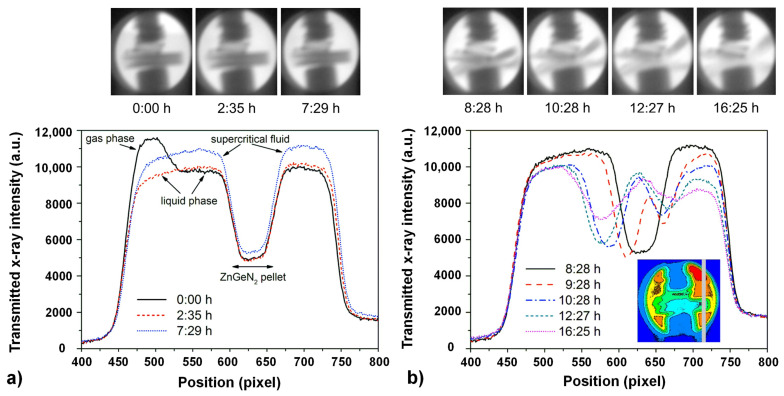
Dissolution of ZnGeN_2_ pellet (pressed powder) over the time, as reported by Häusler et al., analyzed via X-ray absorption imaging [42]. (**a**) First part of the experiment (top: X-ray images, bottom: profile lines through the X-ray image along the line indicated in the inset of subfigure (**b**)), (**b**) first part of the experiment (top: X-ray images, bottom: profile lines through the X-ray image along the line indicated in the inset of subfigure (**b**)). In both subfigures, the X-ray images are reprinted and slightly modified with permission from [38], © 2017 Wiley-VCHVerlag GmbH&Co. KGaA, Weinheim, Germany, and the profile line graphs are reprinted from [299]. The colors in the inset of subfigure (**b**) represent different grayscale values in the X-ray image.

Several binary and ternary nitride materials have linear attenuation coefficients comparable to GaN, as is evident from the right subfigure in Figure 25 of this work (linear attenuation coefficients of additional nitrides can be found in Figure 7 in [304]). Consequently, 2D in situ X-ray imaging has been applied to nitride materials other than GaN, specifically to ZnGeN_2_ [42], which has a linear attenuation coefficient very similar to that of GaN [304] and Zn_3_N_2_ [299]. A specific challenge for investigations on such novel nitrides can be a lack of available bulk material. To some extent, this can be circumvented by using pressed powder samples (microcrystalline) [42]. X-ray images of the dissolution and disintegration of a ZnGeN_2_ pressed powder sample in an ammonobasic experiment with NaN_3_ mineralizer are shown in the upper part of Figure 31. For quantitative analysis of the X-ray images, plotting the grayscale values along a line of interest is helpful. Such profile lines are shown in the bottom part of Figure 31. The Figure 31a,b arbitrarily divide the experiment into two parts for better readability of the profile line graphs. The gas phase, liquid phase, and supercritical phase of ammonia can be distinguished, as indicated in Figure 31a. Importantly, a decrease in transmitted intensity was observed in the fluid regions, in conjunction with the dissolution and disintegration of the pressed powder sample. This indicates that while a mechanical disintegration, with powder particles falling out of the area of view, may also have occurred, part of the increasing X-ray transmission in the region of the sample can confidently be ascribed to at least one element with high X-ray absorption going into the solution from there [42]. Considering that prospective intermediates for an ammonothermal solution of Zn have been reported for several ammonobasic mineralizers (amongst others) [188,347,348], it is expected that Zn at least did go into solution. A remaining limitation of X-ray imaging as a stand-alone method is that contributions of different elements in the solution (such as Zn and Ge in the described case) cannot be distinguished. Combining X-ray imaging with simultaneously conducted spectroscopic techniques (see Section 7.3) is considered to be a promising approach to improving the interpretability of fluid absorption changes in dissolution experiments with ternary and multinary nitrides.

### 7.3. Spectroscopic Techniques

A broad range of spectroscopic techniques has been applied to high pressure systems [349] and more specifically, supercritical fluids [341]. However, only UV/Vis and Raman spectroscopy have been applied to ammonothermal solutions thus far, and we will therefore focus solely on spectroscopic techniques. The fundamental advantage of spectroscopic techniques is that the contributions of different photon energies (wavelengths) to the measurement signal can be distinguished. Unlike the imaging techniques, they can therefore provide insight into chemical bonding and hereby help to identify, for instance, intermediates present in the solution.

#### 7.3.1. UV/Vis Spectroscopy

**Figure 32:**
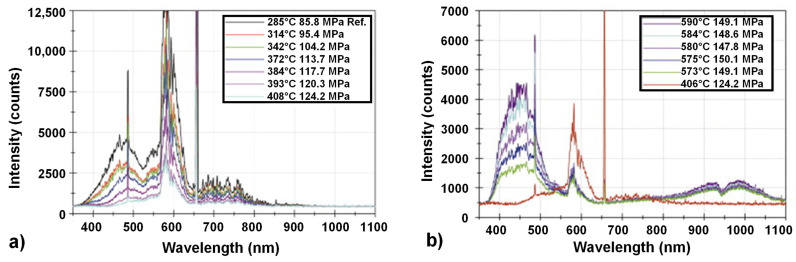
UV/Vis transmission spectra of the GaN/NH_4_Cl/NH_3_ ammonothermal system were examined across a wide range of temperatures, revealing changes in the transmitted intensity across various wavelength regions. Subfigure (**a**) shows the lower, whereas subfigure (**b**) shows the higher temperature range. Reprinted and slightly modified/corrected from [342] with the permission, © 2011 Elsevier B.V.

The feasibility of applying UV/Vis spectroscopy for investigating ammonothermal solutions under process conditions of crystal growth has been demonstrated using a transmission geometry, with a deuterium lamp as the light source to provide sufficient emission in the UV range [298,328]. Changes in the UV/Vis transmission spectra (as well as the reduced transmission observed by the video optical measurements) are thought to be mostly due to changes in fluid absorption, with UV/Vis being the more sensitive technique for detecting these changes [328]. Exemplary UV/Vis transmission spectra are shown in Figure 32, which were obtained by N. Alt and colleagues during their experimentation with GaN in an ammonoacidic environment with an NH_4_Cl mineralizer [342].

Notably, temperatures exceeding 300 °C prompted a consistent decrease in overall light intensity, particularly prominent in the shorter wavelength range. This trend closely paralleled observations from video optical analyses, indicating a concurrent reduction in total light transmission up to approximately 540 °C, beyond which minimal transmission was observed. The observed decline in transmission corresponds well with the documented solubility behavior of GaN in supercritical ammonia solutions containing NH_4_Cl, implying a rapid increase in solubility beyond 300 °C. Moreover, intriguing alterations in the transmitted UV/Vis spectrum were discerned beyond 560 °C, characterized by notable intensity increases within the 380–550 nm range and reductions within the 550 nm to 800 nm range. These changes suggest the potential formation of intermediary compounds arising from reactions between the fluid and GaN powder, hinting at complex chemical transformations occurring at higher temperatures. The changes in different wavelength ranges point towards more than one chemical species contributing to the changes in fluid absorption [342].

However, an unambiguous assignment of specific absorption changes to specific solutes is yet to be demonstrated. A complication for interpretation lies in the possibility of further elements being present in the solution, with corrosion of reactor materials being a main point of concern. N. Alt and colleagues have therefore employed energy-dispersive X-ray spectroscopy and found that indeed several elements such as iron, chromium, or nickel were present in reaction products investigated after the ammonothermal experiment [342]. While exact information about the intermediate species formed has not yet been obtained, UV-Vis spectroscopy has already proven useful in monitoring solubility by spectroscopic means. Care must be taken when interpreting signals in the infrared range, as such signals could potentially be caused by an emission of a chemical compound formed during the experiment, but infrared emission can also be due to the thermal emission of reactor materials. Thus far, infrared emission has only been observed due to thermal emission of reactor components, given that the observed infrared emission did not show any dependency on the amounts of reactants introduced [342].

UV/Vis spectroscopy was also used in investigating the decomposition of ammonia, both with and without additives, experimentally simulating conventional ammonothermal syntheses performed in standard autoclaves [336]. A range of media was utilized, encompassing pure ammonia (NH_3_), ammonia with a ruthenium catalyst, ammonia with sodium azide (NaN_3_), and ammonia with sodium azide and gallium nitride (GaN). A type K thermocouple was fitted for internal temperature monitoring, allowing direct observation of fluid temperature during experimentation. An additional thermocouple was affixed externally to one of the windows. The experimental setup further incorporated heating jackets, high-pressure valves, rupture discs, and connection pipes, enabling meticulous control and monitoring of experimental conditions. Optical measurements were completed using a xenon lamp and spectrometer, also shedding light on the transmittance properties of sapphire windows utilized in the setup [336].

#### 7.3.2. Raman Spectroscopy

Fundamentally, Raman spectroscopy relies on the inelastic scattering of light by matter, that is, the energy of the scattered photon is different from the energy of the incident photon (Raman effect) [350,351]. Due to its sensitivity with respect to crystalline, amorphous, and molecular species, Raman spectroscopy allows the monitoring of phase transitions and chemical reactions [341], and can be used for retrieving information on molecular structures [352]. It has been widely applied to a plethora of subject areas and a variety of variants have been developed to address specific challenges, such as the weak signal of spontaneous Raman scattering [350]. While a number of studies at high temperatures up to 1000 K have been reported, studies at high pressures, or under supercritical conditions, are less common [341]. Nevertheless, some applications to supercritical fluids have been reported, besides those on ammonothermal systems, including studies of phase equilibria in fluid mixtures [353] and contributions to revealing structural organization in binary mixtures, with notable effects on diffusion processes [352].

In the context of ammonia decomposition by T.G. Steigerwald and coworkers [336], Raman spectroscopy emerged as a robust tool for investigating high-pressure systems, thanks to the dense molecular environment involved, as elucidated in Braeuer’s book on spectroscopic techniques for in situ monitoring at high pressure [349]. The adoption of spectroscopic techniques, particularly Raman spectroscopy, holds significant potential for deepening our understanding of ammonothermal synthesis [336,354].

T.G. Steigerwald et al. [336] applied Raman spectroscopy to ammonothermal experiments, utilizing a continuous wave laser emitting green light with a wavelength of 532 nm and adjustable power up to 2 W. The experimental setup involved backscattering, where the laser beam was directed onto the sample in the optical cell, and the backscattered light was collected at a 0° angle using the same optics. To enhance signal clarity, a dichroic mirror was used to separate the redshifted Raman signal from laser reflections and Rayleigh scattered light, while a long-pass filter further refined the sample signal before reaching the spectrometer. Despite these measures, interference from elastically scattered light was observed below a Raman shift of 100 cm^−1^ [336].

**Figure 33:**
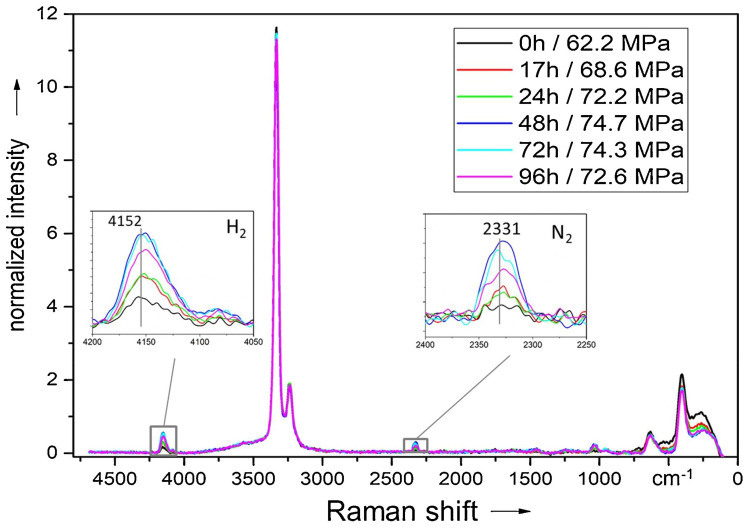
Raman spectra of ammonia under specific experimental conditions; a filling density of ammonia at 8.69 mol/L, a maximum temperature of 563 °C, and a maximum pressure of 74.7 MPa, over an integration time of 100 ms, and a single measurement. Reproduced from [336] with the permission, © 2017 Elsevier B.V.

As part of their study, they employed Raman spectroscopy to analyze pure ammonia, in order to better understand ammonia decomposition kinetics. All the spectra pertaining to this experiment are shown in Figure 33. With a low density of 8.69 mol/L, the experiment aimed to explore how pressure influences the equilibrium of NH_3_ decomposition. Spectra were normalized to the ammonia signal to facilitate comparison. At 563 °C and 70.0 MPa, characteristic peaks for ammonia, nitrogen, and hydrogen were observed, albeit with initially low signal-to-noise ratios for hydrogen and nitrogen peaks due to short integration times. Subsequent measurements showed increased peak intensities over time, reaching their maximum after 48 h [336].

Another focus of the study was to investigate whether the decomposition of NaN_3_ and the formation of NaNH_2_ occur concurrently or independently. Raman spectroscopy was utilized to analyze changes in the electron shell polarizability of nitrogen and hydrogen. Spectra were collected over a duration of 21 h at a consistent temperature of 325 °C, revealing the emergence of N_2_ signals after 3.5 h, coinciding with a progressive increase in intensity alongside a corresponding elevation in pressure from 719 bar to 843 bar [336].

Further experimentation across varying temperatures provided deeper insights into the kinetics of ammonia (NH_3_) decomposition and the catalytic influence of ruthenium. Raman spectroscopy played a crucial role in observing clear signals for ammonia, nitrogen, and hydrogen, facilitating the tracking of their changes over time, and enabling the assessment of the ruthenium catalyst’s influence. Through comparisons of different experiments involving various mineralizers and catalysts, the team identified their respective effects on the speed of ammonia breakdown and the production of nitrogen and hydrogen. These investigations unveiled the complexity of ammonia decomposition and highlighted the significant impact of ruthenium, challenging established notions in autoclave-based ammonothermal synthesis. While Raman spectroscopy provided valuable insights into reaction kinetics and catalyst-mediated processes, there is still a need to unravel the underlying mechanisms governing ammonia decomposition and optimize synthesis protocols, especially concerning gallium nitride production [336].

### 7.4. Ultrasonic Velocity Measurements

In general, ultrasonic techniques can provide information on various properties of materials and local changes of these in an investigated sample, and different ultrasonic parameters such as attenuation, velocity, and acoustic non-linearity can be analyzed and are applied, amongst others, in non-destructive testing [355]. In the field of the ammonothermal method, the application of ultrasonic techniques for in situ monitoring has so far focused on ultrasonic velocity measurements, which were applied for determining the ammonia fill level prior to the ammonothermal experiment [356] and measurements of sodium azide solubility [337]. In addition, a concept for using ultrasonic velocity measurements for determining the viscosity of ammonothermal fluids has been elaborated and is considered to be a particularly promising concept for viscosity measurements under ammonothermal conditions. Both standard tube-shaped autoclaves (fill level, sodium azide solubility) and optical cells (viscometry) have been used for ultrasonic velocity measurements on ammonothermal systems, depending on the measurement goals [325].

Ultrasonic viscometers provide real-time measurement of fluid density and viscosity using acoustic- and shear-impedance analysis, with practical implementations often employing normal incidence configurations for ease of use [357,358,359], which is also the case in the concept that was elaborated for its application to ammonothermal optical cells.

Ultrasonic viscometers utilize either the pulse-echo or through-beam method, both sharing similar construction. The pulse-echo method, which is widely employed, enables easy determination of medium viscosity and density by emitting and capturing sound waves’ reflections [337]. It is adaptable for different mediums and temperatures, exemplified by the Cohen-Tenoudji high-temperature viscometer. However, its reliance on medium and frequency can complicate viscosity determination [360]. Meanwhile, the through-beam method employs two sensors to emit and receive ultrasonic waves, ensuring accurate measurements, even near surfaces. While the pulse-echo method has been preferred for ammonothermal synthesis, through-beam offers potential advantages, particularly considering the need for high-temperature adaptability and application in non-Newtonian fluids [361,362,363].

Additionally, advancements in measurement accuracy and temperature range, up to 1500 °C with cooling measures, highlight the suitability of ultrasonic viscometers for diverse applications, including ammonothermal synthesis, offering insights into fluid behavior at extreme temperatures [364,365,366].

**Figure 34:**
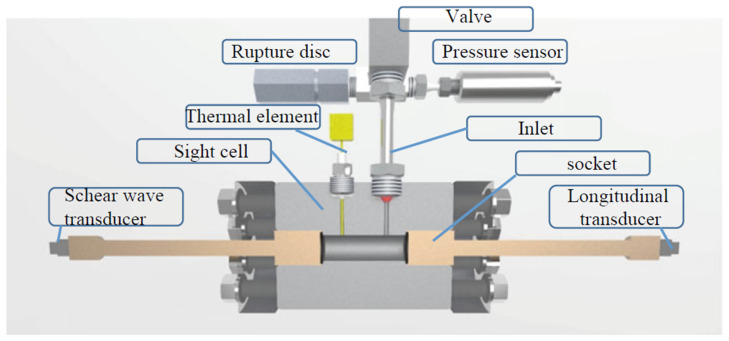
A high-pressure optical cell featuring schematically depicted elements for implementing the ultrasonic pulse-echo measurement technique. Reproduced from [325] with permission, © Springer Nature Switzerland AG 2021.

The fundamental structure of the ultrasonic viscometer, as well as its signal processing methodology, draws upon the groundwork laid by S. H. Sheen et al. [363]. Notably, in the foundational work, only transducers for longitudinal wave measurement had been utilized, limiting accessibility to density information alone [337]. However, the latest design, as depicted in Figure 34, incorporates adjustments to address this limitation. Specifically, two extension bases are affixed to the optical cell, enabling direct opposite positioning. While the simplest configuration involves bases positioned directly opposite to each other, there is consideration for potential angled autoclaves, pending initial measurements to evaluate the added manufacturing complexity against the benefits in terms of accuracy. Additionally, the exact geometries of these bases remain subject to future research, with potential designs exploring staggered surfaces to ensure the requisite reference reflection for accurate measurements. Furthermore, to concurrently determine viscosity, a sensor for shear waves must be integrated, with an emphasis on optimizing space utilization by installing transducers in pulse-echo mode opposite each other [325].

Incorporating optical cells into the viscometer setup, as illustrated in Figure 34, offers several additional advantages. Optical cells can enhance the precision and versatility of viscosity measurements by providing real-time visual feedback on fluid behavior. By leveraging optical techniques such as laser doppler velocimetry or particle image velocimetry, researchers can prospectively gain insights into flow patterns, turbulence, and shear rates, complementing the data obtained from ultrasonic measurements. This integration of optical cells expands the capabilities of the viscometer, allowing for a more comprehensive understanding of fluid dynamics in ammonothermal systems, especially at elevated pressures and temperatures [325].

### 7.5. Rolling Ball Viscosimeter

Rolling ball viscometers, known for their simplicity and robustness, can be highly precise with proper calibration and fabrication, and have been used by L. T. Carmichael and B. H. Sage to measure the viscosity of liquid ammonia across a wide temperature range [367]. Rolling ball viscometers have been adapted for high-pressure and high-temperature applications [368,369], K. Funakoshi and A. Nozawa achieved viscosity measurements even under extreme conditions using X-ray detection [370]. Adjustments to the standard working equation are necessary, as viscosity changes with temperature and is influenced by factors like diameter ratio and the Reynolds number. Despite the challenges, such as returning the ball to its starting position and determining its velocity, rolling ball viscometers meet the requirements for ammonothermal processes. To address these challenges, a high-temperature borne-noise sensor is proposed, utilizing acoustic signals from the rolling ball for viscosity measurement without significantly affecting the temperature profile within the autoclave [371].

Experimental measurements were conducted by T.G. Steigerwald and coworkers [325,371] using the rolling ball viscometer depicted in Figure 19, within a temperature range spanning from room temperature to 588 °C, coupled with pressures reaching up to 100 MPa, validating the operational integrity of the viscometer. Heating was uniformly distributed along the viscometer via three heating sleeves. Diverse materials were employed for the balls to facilitate density determinations, and calibration ensued by employing fluids with precisely known properties. The setup encompassed high-pressure pumps and sensors, alongside a high-temperature borne-noise sensor meticulously crafted for acoustic logging. To refine accuracy, the reproducibility of the viscometer and meticulous calibration with known fluids were ensured, with more detailed description of the setup found in [325,371]. The rolling ball viscometer evinced accuracy and consistency in viscosity measurement up to 588 °C. The inquiry underscores the imperative of compiling fluid properties as a requisite for the ammonothermal process, with the rolling ball viscometer proffering a precise methodology for viscosity and density determination. The feasibility study underscored the promise of a novel high-temperature borne-noise sensor, giving insights into the autoclave, even at higher temperatures. Subsequent investigations are needed to explore variations in ball density to ascertain the viscosity and density of the fluid system within the ammonothermal process, potentially extending the temperature threshold beyond 588 °C [371].

### 7.6. X-ray Diffraction

In recent years, a few researchers have developed technologies for an in situ characterization of crystals during their ammonothermal growth. S. Schimmel and coworkers have developed a setup capable of observing the spatial distribution of intensity within the (0002) reflection of GaN (similarly to a rocking curve) [299,372].

## 8. Summary and Outlook

The ammonothermal method is a promising technique for the synthesis of novel nitrides in crystalline form, along with the development of large scale, single-crystal boules of technologically relevant materials, such as GaN. The method has seen significant advances since its early incarnation for the preparation of amides and imides in the 1960s, to the more rigorous scaling of GaN boules in the 1990s. To date, the method has been adopted worldwide by a few research labs and companies. As we look to the next phase of this method, the synthesis of emerging nitrides, or the experimental realization of theoretically predicted nitrides is becoming a greater focus. There are numerous interesting nitrides that have been identified and actively investigated for single crystal synthesis, including II–IV–N_2_, Mn–IV–N_2_, Al_1−*x*_TM*_x_*N (TM = transition metal), and III–N. These materials have the promise of exhibiting interesting material properties, including strong ferroelectricity, piezoelectricity, non-linear optical properties, spin polarization, and strong magnetization capabilities.

The primary driver for the larger scale adoption and advancement of the technology was the desire to synthesize large boules of GaN for use as substrates in wide bandgap III-N devices. This feat has successfully demonstrated the growth of GaN boules/wafers of up to nearly 4 inches in diameter, with autoclaves capable of growing more than 50 wafers simultaneously, and wafers exhibiting > 1 km wafer curvature, though typically containing relatively high concentrations of oxygen/impurities (typically < 10^19^ cm^−3^) and hydrogenated gallium vacancies (ammonobasic 10^18^–10^19^ cm^−3^/ammonoacidic up to 3 × 10^16^–9 × 10^16^ cm^−3^).

An important feat for enabling the growth of novel nitrides, or advancing growth conditions, is the development of a rigorous understanding of the solubility of species in the solution. Ammonobasic systems heavily rely on alkali (primarily) and alkaline earth (secondarily) metals, while acidic counterparts use halogens. Both systems have successfully demonstrated dissolution of a range of metal cations, with some of them having been investigated to also demonstrate a temperature-dependent solubility, as required for crystal growth. Some important examples include the solubility of Be, Mg, and Si as dopants for III-nitrides, to the transition metals Sc, Ti, Y, Zr, Nb, Hf, and Ta for piezoelectric Al_1−*x*_TM*_x_*N, to Mg, Si, Mn, and Zn as constituents of II–IV–N_2_ semiconductors, and to Ti, V, Zr, Nb, Mo, Hf, Ta, and W as constituents of nitride superconductors. Only for some of these, and for a limited set of ammonothermal conditions, is qualitative information on reactivity and/or solubility available to date.

Acidic ammonothermal systems generally afford higher solubilities and more aggressive dissolution, though this comes at the cost of the autoclave potentially suffering severe corrosive attack. The use of expensive liners (particularly platinum) enabled the use of the ammonoacidic method, although this added a barrier to entry, particularly for applications that require high purity. More recently, less expensive alternatives have emerged, including liner-free autoclaves based on Mo alloy autoclaves and the use of silver as opposed to platinum.

In the realm of III–N materials, AlN and AlGaN are promising materials to explore using this method. Few demonstrations on their growth exist to date, though solubility and deposition have been demonstrated, albeit at a small scale (~few mm in polycrystalline form). The viability of this method to demonstrate growth of the full AlGaN solid solution range has been successfully performed, though further refinements of the method are needed to develop single crystal boules which may one day be useful for applications involving UV light emitters or power electronics.

Most recently, some efforts have started to explore this method for the growth of BN. Demonstrations of its solubility and synthesis, at least for hexagonal and rhombohedral BN, have already been made.

InN is another intriguing nitride that, thus far, has only been synthesized in bulk, single-crystal form using the ammonothermal method. Initial demonstrations suggest a different growth mechanism at play, requiring the use of ammononeutral conditions, through the presence of both an acidic and basic element. Further refinement of this approach has the potential to yield InN substrates, which could permit development of high frequency/speed electronics based on InN, along with infrared emitters/absorbers based on an all-nitride platform.

With regard to emerging materials, ternary nitrides are of great interest. A relatively small set of materials has been demonstrated thus far (including a range of ternary tantalum, silicon, and germanium nitrides and phosphonitrides), partly due to the lack of large-scale investigations. These materials are of current interest, as they can offer an avenue to earth-abundant materials with desired electronic, optical, magnetic, or structural properties.

The ammonothermal method has clearly demonstrated the ability to grow materials with a range of impurities—some desired, such as dopants (O, Si, Mg, Mn), and some less desired, such as metals (Fe, Zn, Na, Al) or atmospheric contaminants (H, O, C), along with the ability to form hydrogenated point defects, such as the observed hydrogenated gallium vacancy (V_Ga_–H_x_). These defects inevitably cause a change in material properties, ranging from changes in p- and n-type doping, along with introduction of defect levels within the band gap, or formation of point defects with intriguing properties. While advantageous at times, this does pose a challenge for the synthesis of a high purity material using this method. Some demonstrations of the reduction of transition metal incorporation and reduction of oxygen (in part via the use of getters, e.g., Er and Al) exist, although these typically come at the price of more complexity, cost, time, or a combination thereof. No uniformly applicable solution to this challenge is on the horizon.

In accordance with impurity incorporation, a wide range of electronic properties have been demonstrated, at least in the case of GaN, ranging for high free electron concentrations (>2 × 10^19^ cm^−3^) and relatively low resistivity (10^−3^ Ω cm). Due to the large number of dopants and defects, mobility in these samples was relatively low. Higher purity material has been demonstrated, yielding higher mobilities up to ~565 cm^2^/(V·s). By doping with higher amounts of Mg in GaN crystals than the background oxygen levels, p-type materials have been demonstrated. Additionally, insulating materials have been demonstrated via the addition of compensating defects, introducing defect levels in the middle of the bandgap. Given the demonstrated breadth of ranges of the incorporation of impurities, desired or otherwise, this suggests the ammonothermal method has sufficient flexibility to control a similar range of impurity incorporation for novel nitrides.

Beyond electronic properties, optical characteristics of impurity containing GaN are detailed. The commonly observed colorations of grey, yellow, green, or orange-brown/red are typically due to free electron concentrations, transition metals, hydrogenated gallium vacancies, or other currently unidentified impurities.

Structural properties of grown nitrides can be directly or indirectly influenced via the variation in free electron concentration, generation, or inclusion of impurities and other point defects, along with heteroepitaxial growth on a non-lattice matched substrate.

Autoclave technologies in general are critical to the successful growth of nitrides from a solution, given the requirement to obtain temperature gradients at high pressure. For the ammonothermal method, tube-shaped autoclaves are generally used for bulk crystal growth, as well as for most other purposes, such as exploratory syntheses. The ammonothermal method has demonstrated multiple co-loaded, multi-inch GaN boules. Refinement of these autoclaves has offered the possibility of new material investigations, and the development of techniques to aid in our understanding of the process. A key advancement is the enhanced corrosion resistance via improved material selection (such as Mo alloy or prospectively Co alloy-based autoclaves), or integration of liners (such as noble metals). The integration of in situ technologies is considered critical to better understanding the system, though it is challenging for these systems given the pressures and corrosive chemicals involved. Nonetheless, notable advances have been made.

Early-stage demonstrations were presented in the integration of a thermocouple into the environment allowing for determination of the actual fluid temperature—a value that was found to be significantly lower than the external wall temperature in smaller scale systems. This approach was then refined to include multiple thermocouple junctions and hence temperature gradients, permitting insights into the growth kinetics, e.g. of GaN, and a better understanding of the heat transfer and fluid flow dynamics in these systems. Fluid temperature measurements also offer valuable insights into convective heat transfer and fluid flow dynamics, complemented by observations of chemical reactions with enthalpy changes. Convective heat transfer significantly impacts temperatures at the outer autoclave wall, prompting questions about suitable boundary conditions for simulations. Clarifying fluid properties such as viscosity, possibly through the validation of simulations with experimental data or dedicated viscosity measurements, is crucial for refining simulations and accurately predicting internal temperature distribution and fluid flow dynamics in ammonothermal growth reactors [127].

Tedious efforts were put forth to identify suitable materials that can be used as windows for optical wavelengths up to high energy X-ray beams. Integration of these windows, including sapphire and B_4_C, has been successfully demonstrated on small scale systems, allowing for imaging of crystals or solutions in situ during various stages of the process, critically at synthesis pressures and temperatures (up to 300 MPa and 600 °C). These systems have successfully provided insights into the physical and chemical properties of the solution and the kinetics of crystal growth and dissolution. Additionally, the integration of X-rays into the system has allowed for the demonstration of X-ray diffraction of a growing GaN crystal, giving unprecedented insight into the growing material from a structural perspective. Furthermore, sealed, yet rotatable feedthroughs have also been demonstrated allowing for sample rotation.

An exciting advancement is on the horizon in the application of X-ray computed tomography during the growth of nitrides in ammonothermal systems. Demonstrations have already yielded novel insight into the growth environment, along with the ability to clearly distinguish and measure materials placed within the autoclave. While these proofs of concepts were made using GaN, they can apply to any material with a sufficiently high X-ray absorption, at energies to which the autoclave is sufficiently transparent.

UV/Vis spectroscopy has successfully measured changes in the chemical composition of the fluid, and was able to link the dissolution of GaN to the associated changes in optical properties of the fluid. These methods can be further refined to enable more rapid solubility measurements of the systems. They could also potentially be applied to growth systems permitting observations during the run and possibly even provide a growth feedback mechanism.

Lastly, ultrasonic velocity measurements have been demonstrated on ammonothermal autoclaves (from room temperature up to 200 °C), offering assessment of liquid fill levels and measurements of sodium azide solubility, while the prospect of its application on heated systems remains viable.

Overall, while there are technological challenges to the adoption of the ammonothermal method, the field has reached a stage of development with robust autoclaves for both ammonoacidic and ammonobasic environments. The method has evolved and is ready to be used for the exploration of nitrides beyond GaN, which has been the primary driver enabling all these advances.

## Figures and Tables

**Table 1:** Characteristics of the hydrothermal growth of quartz and ammonothermal growth of GaN in comparison, with a focus on pressure and temperature range and the properties of the respective fluid under those conditions. Fluid property data were taken from or calculated based on NIST database [131]. Note that the operation range for the ammonothermal method ranges significantly beyond the available data range, as data are only available up to 451.9 °C. Density data of the upper end of the range were linearly extrapolated, viscosity data were extrapolated using a polynomial fit. The data in the table represent the ends of the ranges obtained from NIST database and extrapolation thereof, based on all combinations of pressure and temperature given.

Fluid Property/Process Characteristic	Hydrothermal (Quartz)	Ammonothermal (GaN)
Solvent (critical parameters) [132]	Water (374.5 °C, 22.1 MPa)	Ammonia (132.1 °C, 11.3 MPa)
Temperature range (°C)	345–360 [122]	400–800 [86]
Reduced temperature range *T/T_c_*	0.92–0.96	3.03–6.06
Pressure range (MPa)	70–150 [122]	100–600 [86]
Reduced pressure range *p/p_c_*	3.17–6.79	8.85–53.10
Solvent density (mol/L) in *p*-, *T*-range	40.7–44.2	18.2–36.2
Solvent density (kg/m^3^) in *p*-, *T*-range	733.8–795.8	310.2–616.6
Dynamic viscosity (µPa·s)	90.3–103.2	49.6–284.3
Kinematic viscosity (m^2^/s)	1.230 × 10^−7^–1.296 × 10^−7^	1.597 × 10^−7^–6.228 × 10^−7^

**Table 2:** Overview of identified review and perspective papers and book chapters from 2015–2023 [81,86,87,98,99,100,105,128,142,213,214,215,216] about the ammonothermal growth, focusing exclusively on GaN. Legend/Abbreviations: a: acidic, b: basic, X: topic which was mentioned in the publication at least in some sentences, **F**: the publication focuses on this topic, R: review paper, P: perspective paper, BC: book subchapter, SCoRA: Scalable Compact Rapid Ammonothermal, NEAT: near-equilibrium ammonothermal, SCAAT: Super Critical Acidic Ammonothermal, LPAAT: low-pressure acidic ammonothermal.

Year		2023	2023	2022	2021	2021	2020	2020	2020	2018	2018	2017	2015	2015
Reference		[213]	[86]	[98]	[128]	[214]	[81]	[215]	[99]	[105]	[100]	[87]	[142]	[216]
Publication type		R	R	R	R	BC	P	BC	R	R	BC	R	BC	R
Ammonothermal scope of the article (pages)		4	14	2	25	7	4	5	3	11	13	15	19	3
Miscellaneous	Process	X	X	**F**	X	**F**	X	**F**	X	X	X	X		**F**
	Historical perspective	**F**	X		X					X			**F**	X
	Research group listing		X	X			X	X		X			X	
	Perspective view			X					X					
	Process		X		X		X	X			X	X	X	
Crystal properties	General	X		X	X	X	X			X	X	X	X	X
	Defects	X	**F**			X	X			X	X	**F**	X	
	Purity/doping		X				X		X	**F**	X	**F**	X	
	Device Applications		**F**		X					**F**		X		
Growth conditions	Environment	b & a	b & a	b	b & a	b	b & a	b & a	b	b	b & a	b	b & a	b & a
	Growth rates		X		X					X	X	X	X	
	Morphology	X						X		X	X		X	
	Chemistry	X										X	X	
	Solubility				X						**F**		X	
Growth technology	General	**F**	**F**				**F**		**F**					
	Autoclave	X	X		X						X	X	X	
	SCoRA	X											X	X
	SCAAT/LPAAT	X	X				X							
	NEAT		X											
	2-stage growth		X			X	X	X	X	**F**				
	Tiled seeds		X				X							
	Circular-cross section		X						X					
	Annealing									X				
	Simulations				**F**						X			
	In situ measurements		**F**		**F**									

**Table 3:** Process conditions under which Al dissolved and AlN synthesis was demonstrated. Ascending order according to the layer thickness/grainsize. The mole ratios of D. Peters [173] and B. T.Adekore [174] were numerically solved/calculated from their experimental data.

Temperature Nutrient-Zone (°C)	Temperature Growth-Zone (°C)	Pressure (MPa)	Mineralizer	Solubility	Mol Ratio Al:mineralizer:NH_3_	Growth Time (days)	Product	Layer Thickness-/ Grainsize	Reference
500	600	200	KNH_2_	retrograde	1:0.09:4.5	2.7–4.6	Dense crystalline layers	Some mm	[173]
525	550	246–286	KN_3_	retrograde	1:[1.9–2.6]:[23.5–24.5]	21	Crystalline layers	0.1–1.5 mm	[174]
500	600	100–200	KNH_2_	retrograde	1:0.1:[>4]	n/a	Dense crystalline layers	~1 mm	[160]
<500		400–500	Li, K and LiNH_2_	n/a	n/a	n/a	Crystalline compact grains	≤25 µm	[169]
<550		500	KNH_2_ and LiNH_2_	n/a	1:[n/a]:10	14	Crystalline compact grains as well as small and big needles	Few µm	[220]
400	600	50–200	NH_4_I	retrograde	1:0.05:[>2]	n/a	Crystalline powder	100 nm	[160]
450		ca. 197	NH_4_Cl	n/a	n/a	n/a	Crystalline powder	Ø 32 nm	[159]
350, 400, 450, 500, 550		80–120	NH_4_Cl	n/a	1:1:[n/a]	5-7	Crystalline powder	20–30 nm	[223]

**Table 4:** Nitride materials beyond the III-nitrides that have been synthesized via an ammonothermal synthesis process so far.

Material	Space Group	*p*/MPa	*T*/°C	Mineralizer	Year	Reference
NaTaN_2_	R$\bar{3}$m	100	600	NaNH_2_	1988	[235]
KTaN_2_	Pbca	100	600	KNH_2_	1988	[235]
RbTaN_2_	Pbca	100	600	RbNH_2_	1988	[235]
CsTaN_2_	Fd3m	100	600	CsNH_2_	1988	[235]
Li_2_Ta_3_N_5_	C2/m	600	550	Li/Li_3_N/LiNH_2_	1991	[236]
NaSi_2_N_3_	Cmc2_1_	600	575	NaNH_2_	1993	[237]
K_3_P_6_N_11_	P4_1_32	600	500	KNH_2_	1997	[238]
SrAlSiN_3_	Cmc2_1_	100	500	NaNH_2_	2012	[94]
MgSiN_2_	Pna2_1_	170	797	KN_3_	2017	[36]
MgGeN_2_	Pna2_1_	230	597	NaN_3_	2017	[36]
MnSiN_2_	Pna2_1_	170	797	KN_3_	2017	[36]
MnGeN_2_	Pna2_1_	170	597	NaN_3_	2017	[36]
LiSi_2_N_3_	Cmc2_1_	170	697	LiN_3_	2017	[36]
LiGe_2_N_3_	Cmc2_1_	230	627	LiN_3_	2017	[36]
ZnSiN_2_	Pna2_1_	230	797	KN_3_	2017	[42]
ZnGeN_2_	Pna2_1_	230	797	KN_3_	2017	[42]
Mg_2_PN_3_	Cmc2_1_	140	797	NaN_3_	2018	[58]
Zn_2_PN_3_	Cmc2_1_	200	527	KN_3_	2018	[58]
Mg_0.5_Mn_0.5_SiN_2_	Pna2_1_	150	797	KN_3_	2019	[9]
Mg_0.5_Zn_0.5_SiN_2_	Pna2_1_	150	797	KN_3_	2019	[9]
Mn_0.5_Zn_0.5_SiN_2_	Pna2_1_	150	797	KN_3_	2019	[9]
Mg_0.5_Mn_0.5_GeN_2_	Pna2_1_	200	597	NaN_3_	2019	[9]
Mg_0.5_Zn_0.5_GeN_2_	Pna2_1_	200	597	NaN_3_	2019	[9]
Mn_0.5_Zn_0.5_GeN_2_	Pna2_1_	200	597	NaN_3_	2019	[9]
NaTaN_2_	$R\bar{3}$m	170	800	NaN_3_/KN_3_/RbN_3_/CsN_3_	2019	[186]
KTaN_2_	Pbca	170	800	NaN_3_/KN_3_/RbN_3_/CsN_3_	2019	[186]
RbTaN_2_	Pbca	170	627	NaN_3_/KN_3_/RbN_3_/CsN_3_	2019	[186]
CsTaN_2_	$\mathrm{Fd}\bar{3}$m	170	350	NaN_3_/KN_3_/RbN_3_/CsN_3_	2019	[186]
Sr_3_P_3_N_7_	P2/c	140	797	NaN_3_	2020	[239]

**Table 5:** Current state of impurities in GaN grown via the ammonothermal method with a liner (Zn without a liner). All data which are not individually referenced are from [87,100,105] and were measured by SIMS in bulk GaN.

Impurity	Growth Environment	Impurity Type	Concentration		Potential Impurity Sources	
			Min.	Max.		
Si, Al, Mg	Basic	Minor	High 10^16^	Low 10^17^	GaN nutrient (impurities)	[100]
					Mineralizer (impurities)	[100]
					Autoclave	[146]
Mn, Fe, Zn	Basic	Minor	High 10^16^	Low 10^17^	Autoclave	[140,146]
C	Basic	Minor	High 10^16^	Low 10^17^	Surface contamination	[87,100]
Na	Basic	Minor	low 10^16^	High 10^18^	Mineralizer (solvent)	[87,100]
H	Basic	Major	Low 10^18^	Mid 10^20^	Ammonia (solvent)	[87,100]
	Acidic	Major	Mid 10^17^	High 10^19^	Mineralizer (solvent)	[103,262,263]
O	Basic Acidic	Major Major	Low 10^18^ High 10^17^	Mid 10^20^ High 10^19^	Surface contamination	[87,100]
					Mineralizer (impurities)	[103,262,263]
					GaN nutrient (impurities)	[100,140,252,262]

**Table 6:** Current state of electrical properties of conductive n- and p-type doped conductive and semi-insulating (SI) ammonothermal GaN. The addition (+an.) means that the GaN crystal was annealed under N_2_ atmosphere at 1100 °C for 4 h. The resistivity, charge carrier concentration, and mobility were measured at room temperature.

Process Variant	Dopant	Doping Concentration (cm^−3^)	Conductivity Type	Carrier Concentration (cm^−3^)	Doping Efficiency (%)	Resistivity (Ωcm)	Carrier Mobility (cm^2^/(V·s))	Ref.
basic	O	50–100 × 10^18^	n	6–10^18^	12–10	n/a	68	[282]
basic	O	3.5–10 × 10^18^	n	2.8–7.5 × 10^18^	80–75	1–10 × 10^−3^	ca. 250–200	[105]
acidic	O	1.5–27 × 10^18^	n	0.66–15 × 10^18^	44–56	n/a	565–155	[252]
acidic	O	1–10 × 10^18^	n	0.9–20 × 10^18^	90–50	2–20 × 10^−3^	300–100	[283]
basic	Mg	7–8 × 10^18^	p	n/a	--	10^6^	n/a	[105]
basic (+an.)	Mg	7–8 × 10^18^	p	3.8 × 10^16^	0.54–0.47	30	6	[105]
basic	Mg	1–2 × 10^18^	SI	<10^12^–10^13^	--	≥10^10^	n/a	[105]
basic	Mn	10 × 10^19^	SI	n/a	--	>10^12^	n/a	[105]

**Table 7:** In situ measurement techniques for the ammonothermal method and the derivable information from each of them.

Applicable Measurement Techniques	Required Information
Thermometry	Continuous temperature measurement
X-ray absorption imaging	Crystal dissolution kinetics, solubility, solute concentrations and solvent density changes, potentially crystal growth rates
Computed tomography	Time-dependent etch-back and crystal growth rates, crystal geometry, parasitic deposition, nutrient morphology changes (tracking of mass transport in 3D)
Optical spectroscopy (UV/Vis, Raman)	Monitoring reaction kinetics via composition of the formed compounds Prospectively contribute to the identification of solutes such as intermediates
Viscometry	Viscosity

## Data Availability

No new data were created in this study. So far as new plots of data have been created (involving the specified calculations of average values, standard deviations, and conversions to the in-plane lattice constants), data are available upon reasonable request from the corresponding author S.S.

## Appendix A. Ammonoacidic Conditions

**Table A1:** Available information on reactivity and solubility of elements and their compounds under ammonoacidic conditions. For explanations and interpretation, see the introduction of Section 3 and Section 3.1.

Element Symbol	IUPAC Group	CAS	Reactivity and Solubility Value	Mineralizer	Reference
Ag	11	IB	−1	NH_4_F	[156]
Ag	11	IB	2	NH_4_Cl	[147]
Ag	11	IB	6	NH_4_Br	[157]
Al	13	IIIA	4	NH_4_I	[162]
Al	13	IIIA	4	NH_4_F	[161]
Al	13	IIIA	4	NH_4_Cl	[159]
Al	13	IIIA	7	NH_4_Cl	[159,223]
Au	11	IB	−1	NH_4_Cl	[147]
Au	11	IB	6	NH_4_Br	[157]
B	13	IIIA	6	NH_4_F	[163]
Bi	15	VA	6	NH_4_Br	[157]
Br	17	VIIA	5	NH_4_Br	[152,154,373]
C	14	IVA	6	NH_4_Cl	[164]
Ca	2	IIA	4	NH_4_F	[158]
Cl	17	VIIA	5	NH_4_Cl	[103,285,374]
Co	9	VIIIB	2	NH_4_Cl	[147]
Co	9	VIIIB	-2	NH_4_Cl	[147]
Cr	6	VIB	6	NH_4_C	[164]
Cu	11	IB	6	NH_4_Br	[157]
F	17	VIIA	5	NH_4_F	[84,102,103]
Fe	8	VIIIB	6	NH_4_Cl	[87,164]
Ga	13	IIIA	4	NH_4_F	[133]
Ga	13	IIIA	7	NH_4_F	[84,102,103,149,150,330]
Ga	13	IIIA	7	NH_4_Cl	[103,150,285,319,374,375]
Ga	13	IIIA	7	NH_4_Br	[154]
Ga	13	IIIA	7	NH_4_I	[154]
Ga	13	IIIA	4	NH_4_I	[285,376]
Gd	3	IIIB	4	NH_4_I	[167]
Ge	14	IVA	6	NH_4_Br	[157]
H	1	IA	8	NH_4_F	[84,102,103]
H	1	IA	8	NH_4_Cl	[103,285,374]
H	1	IA	8	NH_4_Br	[152,154,373]
H	1	IA	8	NH_4_I	[103,155,377]
I	17	VIIA	5	NH_4_I	[103,155,377]
Mg	2	IIA	2	NH_4_Cl	[119,147]
Mg	2	IIA	1	NH_4_Cl	[147]
Mo	6	VIB	−1	NH_4_Cl	[147]
Mo	6	VIB	−2	NH_4_Cl	[147]
Nb	5	VB	−1	NH_4_Cl	[147]
Ni	10	VIIIB	2	NH_4_Cl	[147,165,166]
N	15	VA	8	NH_4_F	[84,102,103]
N	15	VA	8	NH_4_Cl	[103,285,374]
N	15	VA	8	NH_4_Br	[152,154,373]
N	15	VA	8	NH_4_I	[103,155,377]
O	16	VIA	6	NH_4_F	[87,103]
O	16	VIA	6	NH_4_Cl	[87,103]
O	16	VIA	6	NH_4_Br	[87,103]
O	16	VIA	6	NH_4_I	[87,103]
Pd	10	VIIIB	−1	NH_4_Cl	[147]
Pt	10	VIIIB	−1	NH_4_Cl	[147]
Pt	10	VIIIB	−2	NH_4_Cl	[147]
Sc	3	IIIB	4	NH_4_I	[167]
Si	14	IVA	−1	NH_4_Cl	[147]
Si	14	IVA	6	NH_4_F	[103]
Si	14	IVA	6	NH_4_Cl	[103]
Si	14	IVA	6	NH_4_Br	[103]
Si	14	IVA	6	NH_4_I	[103]
Sn	14	IVA	6	NH_4_Br	[157]
Ta	5	VB	2	NH_4_Cl	[147]
Ti	4	IVB	2	NH_4_Cl	[147]
Ti	4	IVB	6	NH_4_Cl	[164]
V	5	VB	2	NH_4_Cl	[147]
W	6	VIB	−1	NH_4_Cl	[147]
W	6	VIB	−2	NH_4_Cl	[147]
Y	3	IIB	2	NH_4_Cl	[147]
Y	3	IIIB	4	NH_4_I	[168,378]
Zr	4	IVB	2	NH_4_Cl	[147]
Zr	4	IVB	6	NH_4_Cl	[164]

## Appendix B. Ammonobasic Conditions

**Table A2:** Available information on reactivity and solubility of elements and their compounds under ammonobasic conditions. For explanations and interpretation, see the introduction of Section 3 and Section 3.2.

Element Symbol	IUPAC Group	CAS	Reactivity and Solubility Value	Mineralizer	Reference
Ag	11	IB	−1	NaNH_2_	[147]
Al	13	IIIA	7	NaNH_2_	[147]
Al	13	IIIA	7	KNH_2_	[173,220]
Al	13	IIIA	7	KN_3_	[174]
Al	13	IIIA	7	LiNH_2_	[169,220]
Au	11	IB	2	NaNH_2_	[147]
B	13	IIIA	7	NaNH_2_	[111]
Ba	2	IIA	5	Ba(NH_2_)_2_	[172]
Be	2	IIA	4	NaN_3_	[175]
C	14	IVA	6	NaNH_2_	[145]
C	14	IVA	6	KNH_2_	[176]
Ca	2	IIA	3	NaN_3_	[143,177]
Ca	2	IIA	3	LiNH_2_	[178]
Cd	12	IIB	3	KNH_2_	[119]
Co	9	VIIIB	−1	NaNH_2_	[147]
Co	9	VIIIB	−2	NaNH_2_	[147]
Cr	6	VIB	3	KNH_2_	[179]
Cs	1	IA	5	CsNH_2_	[171,186,348]
Fe	8	VIIIB	6	Na	[87,140]
Ga	13	IIIA	4	Ba(NH_2_)_2_	[172,379]
Ga	13	IIIA	4	LiNH_2_	[96,134,205]
Ga	13	IIIA	4	NaNH_2_	[134]
Ga	13	IIIA	6	NaNH_2_	[95,324,380]
Ga	13	IIIA	6	LiNH_2_	[95]
Ga	13	IIIA	6	KNH_2_	[170,381]
Ga	13	IIIA	7	NaNH_2_	[149,374,382]
Ga	13	IIIA	7	KNH_2_	[109,382]
Ga	13	IIIA	4	KNH_2_	[205,381]
Gd	3	IIIB	4	NaNH_2_	[167,192]
Ge	14	IVA	3	LiNH_2_	[42]
Ge	14	IVA	3	NaNH_2_	[42]
Ge	14	IVA	3	KNH_2_	[42]
Ge	14	IVA	2	NaNH_2_	[147]
H	1	IA	8	NaNH_2_	[84,102,103]
H	1	IA	8	KNH_2_	[170,173,381]
H	1	IA	8	RbNH_2_	[383]
H	1	IA	8	CsNH_2_	[171,186,348]
Ir	9	VIIIB	3	NaNH_2_	[147]
K	1	IA	5	KNH_2_	[170,173,381]
Li	1	IA	5	LiNH_2_	[169,220]
Mg	2	IIA	2	NaNH_2_	[119,147]
Mg	2	IIA	2	KNH_2_	[119,147]
Mg	2	IIA	1	NaNH_2_	[147]
Mg	2	IIA	4	NaNH_2_	[180,191]
Mg	2	IIA	4	NaN_3_	[181,191]
Mg	2	IIA	6	Na	[87,140]
Mn	7	VIIB	4	NaNH_2_	[182,384]
Mn	7	VIIB	6	NaNH_2_	[183]
Mo	6	VIB	−1	NaNH_2_	[147]
Mo	6	VIB	−2	NaNH_2_	[147]
Mo	6	VIB	6	Na	[87,140]
N	15	VA	8	NaNH_2_	[84,102,103]
N	15	VA	8	KNH_2_	[170,173,381]
N	15	VA	8	CsNH_2_	[171,186,348]
N	15	VA	8	RbNH_2_	[383]
Na	1	IA	5	NaNH_2_	[84,102,103]
Nb	5	VB	−1	NaNH_2_	[147]
Ni	10	VIIIB	−1	NaNH_2_	[147,165]
Ni	10	VIIIB	−2	NaNH_2_	[147]
Ni	10	VIIIB	4	NaNH_2_	[184]
O	16	VIA	6	NaNH_2_	[145,146]
O	16	VIA	6	KNH_2_	[176]
P	15	VA	3	NaN_3_	[185,305]
Pd	10	VIIIB	1	NaNH_2_	[147]
Pt	10	VIIIB	2	NaNH_2_	[147]
Pt	10	VIIIB	−2	NaNH_2_	[147]
Rb	1	IA	5	RbNH_2_	[383]
Si	14	IVA	4	KNH_2_	[119,385]
Si	14	IVA	1	NaNH_2_	[147]
Si	14	IVA	3	KNH_2_	[36,42]
Si	14	IVA	6	Na	[87,140]
Ta	5	VB	2	NaNH_2_	[147]
Ta	5	VB	3	NaN_3_	[186]
Ta	5	VB	3	KN_3_	[186]
Ta	5	VB	3	RbNH_2_	[186]
Ti	4	IVB	1	NaNH_2_	[147]
V	5	VB	2	NaNH_2_	[147]
W	6	VIB	−1	NaNH_2_	[147]
W	6	VIB	−2	NaNH_2_	[147]
Y	3	IIIB	2	NaNH_2_	[147]
Y	3	IIIB	3	KNH_2_	[187]
Zn	12	IIB	4	KNH_2_	[188]
Zn	12	IIB	6	KNH_2_	[189]
Zr	4	IVB	2	NaNH_2_	[147]

## Appendix C. Ammononeutral Conditions

**Table A3:** Available information on reactivity and solubility of elements and their compounds under ammononeutral conditions. For explanations and interpretation, see the introduction of Section 3 and Section 3.3.

Element Symbol	IUPAC Group	CAS	R and S Value	Mineralizer	Reference
Al	13	IIIA	4	None	[190,386,387]
Al	13	IIIA	4	None	[173]
Au	11	IB	−2	None	[147]
Ba	2	IIA	4	None	[181,194,195,388]
Be	2	IIA	4	None	[191]
Ca	2	IIA	4	None	[195,196,197,198,199,389]
Ce	3	IIIB	4	None	[192,390,391]
Co	9	VIIIB	−1	None	[147]
Co	9	VIIIB	−2	None	[147]
Cs	1	IA	4	None	[378,392]
Cs	1	IA	5	n(InI_3_):n(CsNH_2_) = 1:3	[200]
Cu	11	IB	4	None	[193,393]
Eu	3	IIIB	4	None	[187,195,199,201,210]
Fe	8	VIIIB	4	None	[202,394,395]
Ga	13	IIIA	4	None	[133,134,396]
Ge	14	IVA	4	None	[147]
Gd	3	IIIB	4	None	[192,203]
H	1	IA	8	None	[111,141,182,200,348,397]
H	1	IA	6	None	[241,254]
In	13	IIIA	6	n(InF_3_):n(KNH_2_) = 1:3.12	[144]
In	13	IIIA	7	n(InCl_3_):n(KNH_2_) = 1:3	[141,144,200]
In	13	IIIA	7	n(InI_3_):n(CsNH_2_) = 1:3	[200]
K	1	IA	5	n(InCl_3_):n(KNH_2_) = 1:3	[141,200]
K	1	IA	5	None	[193,397]
La	3	IIIB	4	None	[192,398,399,400,401]
La	3	IIIB	2	None	[147]
Li	1	IA	4	None	[206,402]
Mg	2	IIA	4	None	[180,207,208]
Mn	7	VIIB	4	None	[182,205,211,384,394]
Mo	6	VIB	−1	None	[147]
Mo	6	VIB	−2	None	[147]
N	15	VA	8	Neutral with InX_3_ and KNH_2_	[200,397]
N	15	VA	8	None	[111,182,348]
N	15	VA	6	None	[403,404]
Na	1	IA	4	None	[195,196]
Nb	5	VB	−1	None	[147]
Nd	3	IIIB	4	None	[192,391,400]
Ni	10	VIIIB	−2	None	[147]
Pd	10	VIIIB	−1	None	[147]
Pt	10	VIIIB	−2	None	[147]
Rb	1	IA	4	None	[195,198,208]
Sc	3	IIIB	−1	None	[147]
Si	14	IVA	−1	None	[147]
Si	14	IVA	−2	None	[147]
Sm	3	IIIB	4	None	[192,209,391]
Sr	2	IIA	4	None	[195,196,199]
Ta	5	VB	3	None	[147]
Th	3	IIIB	4	None	[181,405]
V	5	VB	3	None	[147]
W	6	VIB	−1	None	[147]
W	6	VIB	−2	None	[147]
Y	3	IIIB	4	None	[168,187,203,400,406]
Yb	3	IIIB	4	None	[168,187,201,203,210,400]
Zn	12	IIB	4	None	[211]
Zr	4	IVB	−1	None	[147]
Zr	4	IVB	4	None	[212,407]
